# Photocatalytic Hydrogenation of Alkenes Using Water as Both the Reductant and the Proton Source

**DOI:** 10.1002/advs.202406046

**Published:** 2024-10-09

**Authors:** Xinzhe Tian, Ming Qiu, Wankai An, Yun‐Lai Ren

**Affiliations:** ^1^ College of Science Henan Agricultural University Zhengzhou Henan 450002 P. R. China

**Keywords:** alkenes, hydrochloric acid, hydrogenation, photocatalysis, water

## Abstract

Utilization of clean and low‐cost water as the reductant to enable hydrogenation of alkenes is highly attractive in green chemistry. However, this research subject is considerably challenging due to the sluggish kinetics of the water oxidation half‐reaction. It is also very difficult to avoid the undesired oxidation of alkenes because that this oxidation is far easier to occur than the desired oxidation of water from thermodynamic standpoint. Herein, this challenge is overcome by applying a cooperative catalysis where HCl is used as the cocatalyst to accelerate Pt/g‐C_3_N_4_‐catalyzed water oxidation and suppress the undesired oxidation of the alkene. This provides an example for using water as the reductant and the proton source to enable the photocatalytic hydrogenation of alkenes. The present method exhibits broad substrate applicability, and allows various arylethenes and aliphatic alkenes to undergo the hydrogenation smoothly.

## Graphical abstract

An example is reported for using green and inexpensive water as the reductant and the proton source to enable photocatalytic hydrogenation of alkenes under an unprecedented cooperative catalysis between Pt/g‐C_3_N_4_ and HCl, in which HCl can accelerate the desired water oxidation half‐reaction and suppress the undesired oxidation of alkenes.

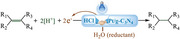

## 1 Introduction

**Scheme 1:**
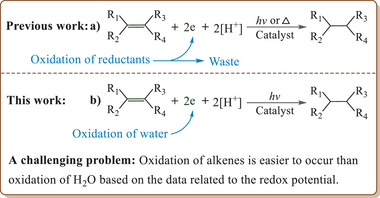
Photocatalytic hydrogenation of organic molecule using H_2_O as a hydrogen source.

Hydrogenation of alkenes is a class of important organic transformations because they often serve as an indispensable process in the production of fine chemicals, pharmaceuticals, and agrochemicals.^[^ ^1^ ^]^ As a result, a great deal of attention has been paid to this class of reactions,^[^ ^2^ ^]^ and several strategies have been developed.^[^ ^3^ ^]^ One is the catalytic strategy using dangerous hydrogen gas as the hydrogen donor under high temperatures and H_2_ pressures.^[^ ^4^ ^]^ Another strategy is the transfer hydrogenation reactions, which refers to the transfer of the hydrogen atom to the target molecule from hydrogen‐rich molecules including alcohols, amines, hydrazines and so on.^[^ ^5^ ^]^ Compared with the above‐mentioned hydrogen donors, water is a more ideal alternative owing to its inexpensive, readily available and environment‐friendly characters, which has prompted chemists to utilize water as the proton source to enable hydrogenation of alkenes.^[^ ^6^ ^]^ However, normal conditions do not allow the H_2_O molecule to be decomposed to O_2_ and the zero‐valent hydrogen due to the high Gibbs free energy (Δ*G* ^0^ = +237 kJ mol^−1^).^[^ ^7^ ^]^ Thus thermochemical methods for using water as the proton source have to rely on a use of reducing metals including zinc powder,^[^ ^8^ ^]^ magnesium powder,^[^ ^9^ ^]^ iron powder,^[^ ^10^ ^]^ aluminum powder^[^ ^11^ ^]^ and samarium diiodide^[^ ^12^ ^]^ to reduce H^+^ of H_2_O to the active hydrogen, or to reduce the substrate molecules (**Scheme** **1**a). Recently, Xu and co‐workers have reported a seminal work for using water as the proton source to enable photocatalytic hydrogenation reactions,^[^ ^7a^ ^]^ where Pt/g‐C_3_N_4_ and triethanolamine are used as the catalyst and the reductant, respectively. Subsequently, many photochemical methods for using water as the proton source in the photocatalytic hydrogenation have been developed (Scheme 1a),^[^ ^13^, ^14^, ^15^, ^16^, ^17^, ^18^ ^]^ and all of these methods require a use of pollutive reductants including triethanolamine,^[^ ^13^ ^]^ alcohols,^[^ ^14^ ^]^ Na_2_SO_3,_^[^ ^15^ ^]^ Mg^[^ ^16^ ^]^ or *N*,*N*‐diisopropylethylamine.^[^ ^17^ ^]^ In addition, it is worth mentioning that a photocatalytic phosphine‐mediated method has been reported with water as the proton source and triphenylphosphines as the reductant.^[^ ^18^ ^]^

Theoretically, the use of additional reductants can be avoided because water molecule has potential to serve as an electron donor via the half‐reaction related to oxidation of O^2−^ of H_2_O to O_2_. Thus the objective of this work was set to develop an additional reductant‐free procedure for hydrogenation of alkenes by using the released electron from the water oxidation to reduce the substrate or the H^+^. Considering that graphitic carbon nitride (g‐C_3_N_4_)‐based materials could serve as effective photocatalysts for the proton reduction,^[^ ^19^, ^20^ ^]^ the hydrogenation^[^ ^19^, ^20^ ^]^ and other reactions.^[^ ^19^, ^20^ ^]^ we selected transition metal/g‐C_3_N_4_ as the catalyst to make our idea come true. However, this subject is considerably challenging due to the following reasons: i) According to the data related to the redox potential,^[^ ^21^ ^]^ oxidation of alkenes is easier to occur than oxidation of H_2_O (Scheme 1b), thus it is very difficult to avoid oxidation of alkenes in the case of using H_2_O as the reductant. ii) The oxidative half‐reaction of H_2_O to O_2_ suffers from sluggish kinetics and slows down the overall process.^[^ ^21^, ^22^ ^]^ Fortunately, we have overcome these challenges by means of a cooperative catalysis between Pt/g‐C_3_N_4_ and HCl, and the results are herein reported (Scheme 1b).

## 2 Results and Discussion

**Figure 1:**
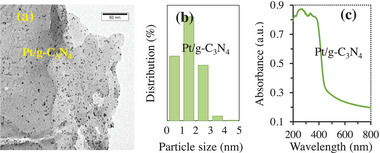
Samples of 0.9 wt% Pt/g‐C_3_N_4_: a) TEM images. b) Particle size distribution. c) UV–visible diffuse reflection spectra.

Pt/g‐C_3_N_4_ was prepared by a thermal reduction method with K_2_PtCl_6_ and g‐C_3_N_4_ nanosheets as the reaction materials based on previous procedures in literatures.^[^ ^20^, ^23^ ^]^ As seen from the transmission electron microscope (TEM) image (**Figure** **1**a), Pt nanoparticles are uniformly deposited on g‐C_3_N_4_, and have a narrow size distribution centered at 0–3 nm (Figure 1b). The light harvesting capability of 0.9 wt% Pt/g‐C_3_N_4_ was also examined, and Figure 1c shows that this material displays a stronger photoresponse at 200–380 nm. With increasing the wavelength from 380 to 490 nm, the photoresponse quickly becomes weaker. When the wavelength exceeds the photoabsorption edge (490 nm), this semiconductor still exhibits an evident optical absorption, which results possibly from the surface plasmon resonance effects of Pt loaded in g‐C_3_N_4_ structure.^[^ ^24^ ^]^

**Figure 2:**
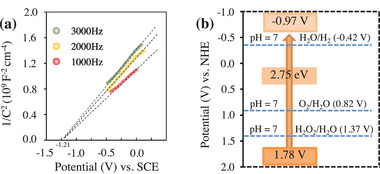
Samples of 0.9 wt% Pt/g‐C_3_N_4_: a) Electrochemical Mott‐Schottky plots. b) Energy‐band positions.

The bandgap value of this semiconductor was calculated to be 2.75 eV based on Tauc's equation (Figure S2b, Supporting Information). The conduction band position was identified to be −0.97 V versus normal hydrogen electrode (NHE) based on the results from electrochemical Mott‐Schottky experiments (**Figure** **2**a). According to the bandgap value and the conduction band position, the valence band position was estimated to be 1.78 V, ignoring the difference between the flat bandgap and the conduction band. As shown in Figure 2b, the reduction level for H_2_ is below the conduction band,^[^ ^21^, ^25^ ^]^ and the oxidation level for H_2_O to O_2_ is positioned above the valence band,^[^ ^21^, ^25^ ^]^ which reveals that Pt/g‐C_3_N_4_ has the potential to function as a photocatalyst for the overall water splitting.

**Figure 3:**
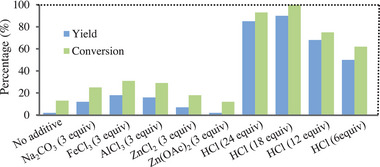
Pt/g‐C_3_N_4_‐catalyzed hydrogenation of 4‐chlorostyrene in the presence of various cocatalysts (for the reaction conditions, see Figure S3 in the Supporting Information).

Subsequently, the performance of Pt/g‐C_3_N_4_ was evaluated in the photocatalytic hydrogenation of 4‐chlorostyrene. As illustrated in **Figure** **3**, when Pt/g‐C_3_N_4_ was used, the targeted product was obtained in very low yield (only 2%), and the main product was the undesired 4‐methoxybenzaldehyde from oxidation of the alkene.^[^ ^26^ ^]^ These results revealed that Pt/g‐C_3_N_4_ was less effective as the photocatalyst for the hydrogenation. We attempted to improve the catalytic performance by changing various reaction conditions including temperature, solvent, wavelength of light, reaction time and Pt loading, but all these attempts were unsuccessful. As seen from Figure 3, the efficiency of NaCO_3_ or FeCl_3_ as the cocatalyst was unsatisfactory, while they had been demonstrated to have remarkable ability to improve the catalytic performance of transition metal/g‐C_3_N_4_ toward overall water splitting.^[^ ^20d,e^ ^]^ Similarly, the reaction was not significantly accelerated by adding AlCl_3_, ZnCl_2_ or Zn(OAc)_2_ as the cocatalyst. Fortunately, a use of HCl as the cocatalyst allowed the targeted product to be obtained in a high yield (92%). These results suggested that HCl was an effective cocatalyst to assist Pt/g‐C_3_N_4_ to catalyze the hydrogenation. The used amount of HCl was also optimized, and the results revealed that 18 equiv. HCl was the most effective.

**Figure 4:**
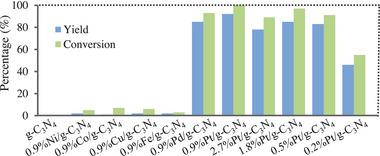
Hydrogenation of 4‐chlorostyrene with various catalysts (for the reaction conditions, see Figure S4 in the Supporting Information. x%M/g‐C_3_N_4_ means that the actual loading amount of the metal in M/g‐C_3_N_4_ is x wt%. M represents the metals).

Further studies were undertaken to search for effective photocatalysts in the case of using HCl as the cocatalyst (**Figure** **4**). When pure g‐C_3_N_4_ served as the photocatalyst, no targeted product was obtained, suggesting that transition metals on g‐C_3_N_4_ was indispensable for the present reaction. After several transition metals including Ni, Co, Cu, Fe, Pd, and Pt were screened, it was found that Pt was the most effective. Pt/g‐C_3_N_4_‐catalyzed reaction afforded the hydrogenation product as high as 92% yield. When Pt/g‐C_3_N_4_ was replaced by Pd/g‐C_3_N_4_, a high yield (85%) was also obtained, while the targeted product was obtained in very low yields in the case of using g‐C_3_N_4_‐supported Ni, Co, Cu or Fe as the photocatalyst. Afterward, an influence of the Pt loading amount on the hydrogenation was examined, and the results revealed that 0.9 wt% Pt loading was optimal (Figure 4). The yield gradually increased with increasing the Pt loading from 0.2 to 0.9 wt%. Then the yield gradually decreased with increasing the Pt loading from 0.9 to 2.7 wt%, which was possibly owing to that the aggregation of excess Pt nanoparticles would lead to the charge recombination.^[^ ^7a^ ^]^

**Figure 5:**
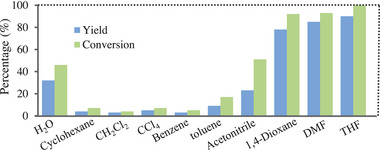
Hydrogenation of 4‐chlorostyrene with various solvents (for the reaction conditions, see Figure S5 in the Supporting Information).

Effects of the solvent change on the hydrogenation reaction were also investigated (**Figure** **5**). When water was used as the solvent, only 46% substrate was converted and 32% yield of the hydrogenation product was obtained. According to our observation, the photocatalyst could not be effectively dispersed in water, which was possibly the main reason why pure water was less effective as the solvent. Therefore, organic solvents had to be mixed with water to improve the dispersion of the photocatalyst in the solvent. When water/cyclohexane, water/CH_2_Cl_2_, water/CCl_4_, water/benzene and water/toluene were respectively used as the solvent, no effective dispersion of the photocatalyst in the reaction system was observed, and the hydrogenation reaction did not go well. On the contrary, the mixed solvents including water/1,4‐dioxane, water/DMF and water/tetrahydrofuran allowed the catalyst to be effectively dispersed, and allowed the targeted product to be obtained in 61–85% yields. It is worth mentioning that water/tetrahydrofuran is the most effective solvent for the present reaction based on these experimental results (Figure 5). In addition, the loading amount of water was optimized, and the obtained results revealed that 3 mL water was optimal (see Table S2, Supporting Information).

**Figure 6:**
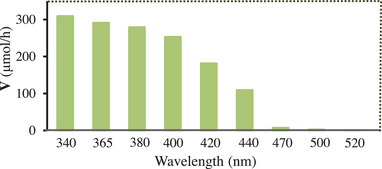
Effect of the wavelength variation on hydrogenation of 4‐chlorostyrene (for the reaction conditions, see Figure S6 in the Supporting Information). *V*: average rate regarding production of 4‐ethylchlorobenzene in 20 min.

Next, we examined the photocatalytic hydrogenation under different monochromatic light sources. As listed in **Figure** **6**, with increasing gradually the wavelength from 340 to 440 nm, the produced rate of 4‐ethylchlorobenzene gradually dropped. This variation tendency matches well with that regarding the photoabsorption of the catalyst (Figure 1c), revealing that the reaction rate is dependent on the photoresponse of the catalyst, and that the present hydrogenation is mainly driven by the photoexcitation of the catalyst.^[^ ^20d^ ^]^ When the wavelength exceeded the intrinsic photoabsorption edge (446 nm) of this semiconductor catalyst, the present hydrogenation was very sluggish, which suggests that the present hydrogenation is mainly triggered by the intrinsic photoabsorption of the semiconductor.^[^ ^20d^ ^]^ On the other hand, the irradiation of more than 446 nm light can result in a weak extrinsic optical absorption (Figure 1c).^[^ ^20d^ ^]^ Thus it is possible that the present reaction is driven by the non‐semiconductor photocatalysis that results from the extrinsic optical absorption, which is apparently the main reason why the reaction can proceed sluggishly at 470 nm wavelengths (Figure 6).^[^ ^20d^ ^]^

**Scheme 2:**
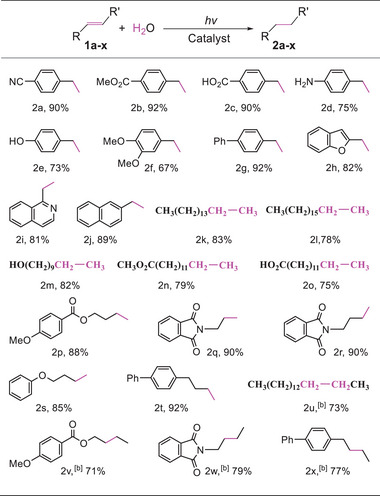
Photocatalytic hydrogenation of various alkenes. ^[a]^Standard condition: 0.2 mmol alkene, 15 mg Pt/g‐C3N4 (0.9 wt% Pt), 0.3 mL aqueous solution of HCl (37 wt%), 3 mL H2O, 5 mL THF, 1.5 h, light irradiation (75 W LED, 365 nm wavelength, actual incident light intensity: 0.175 W cm−2. [b] The reaction time was 2 h in the case of 2u‐2x.

Subsequently, we evaluated the reactivity of various representative alkenes under the optimized conditions, and the results were listed in **Scheme** **2**. In general, the present method showed broader substrate applicability, and allowed various arylethenes and aliphatic alkenes to be smoothly hydrogenated. Moreover, the present reaction exhibited good functional group tolerance. For example, cyano, ester, carboxyl, amino, phenolic hydroxyl, alkoxyl and hydroxyl groups were well‐tolerated. When electron‐withdrawing group (EWG)‐substituted styrenes were employed as the substrates, the hydrogenation reactions proceeded smoothly, and the targeted products were obtained in high yields (2a‐2c). The major byproducts were α‐phenylethanols and acetophenones,^[^ ^27^ ^]^ which resulted from the addition of water to the double bonds and the dehydrogenation of α‐phenylethanols, respectively. The present condition also allowed electron‐donating group (EDG)‐substituted styrenes to undergo complete transformation (see 2d‐2f), but the targeted products were obtained in lower yields due to the formation of larger amount of by‐products that were produced by the addition of water to the double bonds. In addition, EDG‐substituted styrenes gave larger amount of oxidation products of the alkenes, suggesting that these alkenes are easier to be oxidated than EWG‐substituted styrenes, which is possibly owing to that π‐bonds of the former have a higher electron cloud density.

Another kind of suitable substrates was heteroarylethenes. For example, both 2‐vinylbenzofuran and 2‐vinylisoquinoline were smoothly converted to the targeted products (2 h and 2i) in 82% and 81% yields, respectively. 2‐Vinylnaphthalene was also smoothly hydrogenated to 2‐ethylnaphthalene products (2j). The present method can be applied into the hydrogenation of aliphatic alkenes. As seen from Scheme 2 (2k‐2t), a variety of terminal aliphatic alkenes underwent smoothly the hydrogenation to give the targeted products in moderate to high yields. Compared with terminal alkenes, 1,2‐disubstituted alkenes had a lower reactivity, therefore the reaction time had to be prolonged to 2 h to complete the conversion (2u‐2x). These results suggested that the steric hindrance had a significant effect on the reaction. Indeed, trisubstituted and tetrasubstituted alkenes including 2‐methyl‐1‐phenylpropene, 2‐methyl‐3‐phenyl‐2‐butene, 1,1‐diphenyl‐1‐propene, 2‐methyl‐1,1‐diphenyl‐1‐propene and 4‐methyl‐1,2‐dihydronaphthalene that have a larger steric hindrance around the π‐bonds were less reactive.

**Scheme 3:**
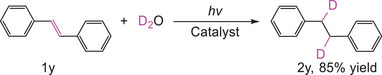
Photocatalytic deuterization of alkenes. For experimental procedures, see Unit 5 in the Supporting Information.

The present method could be applied into the deuterization of alkenes in the case of using D_2_O to replace H_2_O. As shown in **Scheme** **3**, alkene 1y was smoothly converted to the deuterated product in high yield with good deuterium incorporation. This is obviously a good method for synthesizing high value‐added deuterated compounds based on the following fact: Compared with other deuteration reagents including DCOONa, CD_3_CN, D_2_, PhSiD_3_, CD_3_OD and so on,^[^ ^28^ ^]^ D_2_O is more attractive from the perspective of safety, cost, and handling.

**Scheme 4:**
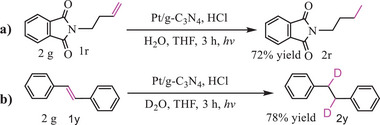
Reactions on 2 g scale. For experimental procedures, see Unit 5 in the Supporting Information.

The present procedure is readily applicable to a multigram‐scale preparation for the targeted product. For example, hydrogenation of alkene 1r proceeded efficiently on 2 g scale under our condition, and gave the targeted product in 72% GC yield (**Scheme** **4**a). Deuterization of alkene 1y afforded the deuterization product in 78% GC yield (Scheme 4b).

**Figure 7:**
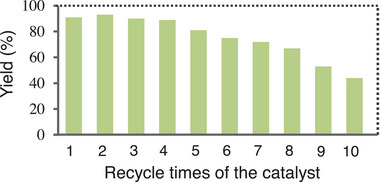
Catalytic recycling test of Pt/g‐C_3_N_4_ in hydrogenation of 4‐chlorostyrene (for the reaction conditions, see Unit 6.1 in the Supporting Information).

Subsequently, our investigation was focused on the recyclability of the catalyst. Pt/g‐C_3_N_4_ was easily recovered via centrifugation or filtration from the reaction system, and then used in the next catalytic cycle. As seen from **Figure** **7**, Pt/g‐C_3_N_4_ was recycled for four times with a very slight change in the catalytic efficiency, and the yield of the hydrogenation product decreased after five cycling runs. The Pt/g‐C_3_N_4_ catalysts before and after five cycling runs were characterized by IR, XRD, XPS, SEM, ICP‐MS, TEM and mapping analysis (see Figures S7–S13 in the Supporting Information). The obtained results suggest that five cycling runs didn't lead to remarkable changes in the structure and morphology of the catalyst. In addition, there was not a significant change in the valence state of Pt before and after the reaction based on the XPS analysis. However, according to the results related to the ICP‐MS, TEM and mapping analysis, the Pt loading amount became significantly lower after five cycling runs, which is obviously one of the main reasons for the decrease in the catalytic efficiency after five cycling runs. Another reason is that the loss of the catalyst is inevitable during recovering the catalyst based on our observation.

**Scheme 5:**
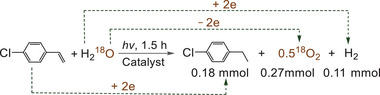
Investigation on who is the electron donor (for the reaction conditions, see Figure S14 in the Supporting Information).

By all appearances, a presence of the reducing reagent as the electron donor is necessary to reduce the substrate molecules or H^+^ of H_2_O based on the conservation law of electric charge. In order to clarify who plays the role of the reducing agent, we performed a H_2_^18^O‐labelling experiment where an addition of H_2_^18^O into the reaction system resulted in the formation of ^18^O_2_ product (**Scheme** **5**). In addition, the results showed that the molar ratio of the reduction products (4‐ethylchlorobenzene and H_2_) and the oxidation product (^18^O_2_) was roughly equal to one (Scheme 5), which is consistent with the theoretical value.^[^ ^20d,e^ ^]^ These results reveal that water serves as the electron donor via oxidation of O^2−^ in H_2_O to O_2_ in the present reaction. It is possible that THF or the benzene ring serves as the electron donor by the oxidation of them. However, this possibility was ruled out based on the following fact: no detectable amount of products from the oxidation of THF or the benzene ring was observed.

**Scheme 6:**
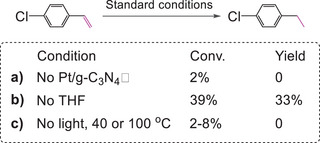
Control experiments for hydrogenation of 4‐chlorostyrene (for the reaction conditions, see Figure S15 in the Supporting Information).

As shown in **Scheme** **6**, several control experiments were performed to clarify the necessity for using Pt/g‐C_3_N_4_, THF or light. When no Pt/g‐C_3_N_4_ was used, the hydrogenation reaction did not occur (Scheme 6a), which revealed that Pt/g‐C_3_N_4_ was indispensable as the catalyst for the present hydrogenation. The targeted product was obtained in a low yield in the absence of THF (Scheme 6b), indicating that THF played an important role in the present reaction. Based on our observation, the catalyst had a poor dispersion in the pure water, which revealed that THF served as the dispersant in the present reaction. We also performed the control experiments under irradiation‐free condition, but hardly any hydrogenation product was observed (Scheme 6c), suggesting that the present transformation is a light‐dependent reaction.

**Scheme 7:**
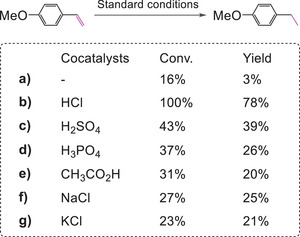
Control experiments for hydrogenation of 4‐methoxystyrene (for the reaction conditions, see Figure S16 in the Supporting Information).

As shown in **Scheme** **7**a, the main product was 4‐methoxybenzaldehyde from the oxidation of the substrate in the absence of HCl. When HCl was added into the reaction system (Scheme 7a vs b), the yield of the targeted product increased from 3% to 78%, while that of 4‐methoxybenzaldehyde product from oxidation of this alkene dropped from 9% to 3%. These results revealed that HCl not only accelerated the hydrogenation, but also increased the selectivity of the hydrogenation product by suppressing the oxidation of the alkene. When HCl was replaced by H_2_SO_4_, H_3_PO_4_ or CH_3_CO_2_H, the yield of the targeted product decreased from 78% to 39%, 26% and 20%, respectively (Scheme 7c–e). The hydrogenation also became more sluggish in the case of using NaCl or KCl instead of HCl (Scheme 7f,g). These results suggested that both H^+^ and Cl^−^ were responsible for promoting the present reaction.

**Figure 8:**
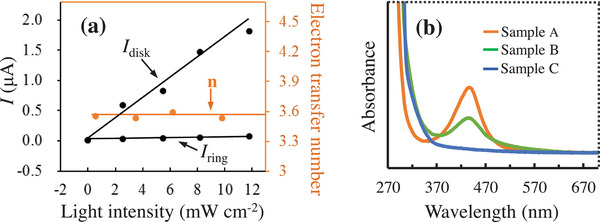
a) Results from rotating disk‐ring electrode (RRDE) experiments. *I*_disk_: current of disk, *I*_ring_: current of ring. *n*: the electron transfer numbers. b) UV–vis spectroscopy related to the produced H_2_O_2_ in hydrogenation of 4‐chlorostyrene. Sample A: 0.4 mmol L^−1^ aqueous solution of H_2_O_2_; Sample B: the produced H_2_O_2_ under standard conditions in Scheme 2 (no HCl and 0.5 h of reaction time); Sample C: the produced H_2_O_2_ under standard conditions in Scheme 2 (0.5 h of reaction time).

Afterwards, our attention was turned to the investigation on the reason why HCl can promote the reaction. According to previous literatures, the water oxidation half‐reaction often yields H_2_O_2_ that poisons g‐C_3_N_4_‐based photocatalyst.^[^ ^29^ ^]^ Maybe HCl can prevent the photocatalyst from being poisoned by removing the produced H_2_O_2_ or suppressing the formation of H_2_O_2_. In order to verify this possibility, we performed the rotating ring‐disk electrode (RRDE) experiments to clarify whether the mechanism pathway of the water oxidation involves the production of H_2_O_2_. Based on previous literatures,^[^ ^20^, ^21^ ^]^ the electron‐transfer number is 4 for H_2_O_2_‐free one‐step process (2H_2_O → O_2_ + 4H^+^), while the electron‐transfer number is 2 for the two‐step process (2H_2_O → H_2_O_2_ → 0.5O_2_ + H_2_O). Our results show that the electron‐transfer numbers range from 3.53 to 3.59 which are close to 4 (**Figure** **8**a), which reveals that the H_2_O_2_‐free process serves as the major pathway under our conditions, that is to say that no H_2_O_2_ intermediate is produced in the major mechanism pathway.

In addition, we analyzed the reaction system using UV–Vis spectroscopy with *o*‐tolidine as the indicator of H_2_O_2_ based on the typical absorption peak at 438 nm.^[^ ^29b^ ^]^ As seen from Figure 8b, the H_2_O_2_ formation was confirmed for HCl‐free reaction system, while no H_2_O_2_ was observed for the reaction system in the presence of HCl. Based on these results and the RRDE experimental results, it is concluded that HCl can suppress the formation of H_2_O_2_ that poisons the photocatalyst, and changes the mechanism pathway from the H_2_O_2_‐involved one to easier H_2_O_2_‐free one, which is apparently one of the main reasons why HCl can promote the reaction.

**Scheme 8:**
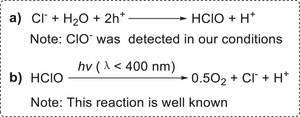
Cl^−^‐triggered oxidation half‐reaction of water.

According to the above results, it is inferred that Cl^−^ participates in the water oxidation half‐reaction. Indeed, Cl^−^ can be theoretically oxidized to ClO^−^ by the photogenerated holes (**Scheme** **8**a) because the redox potential for ClO^−^/Cl^−^ (1.64 V)^[^ ^30^ ^]^ is more negative than the valence band position (1.78 V, see Figure 2b). Moreover, ClO^−^ intermediate was detected in our conditions. In addition, it is well known that HClO can be decomposed to O_2_ and Cl^−^ under the irradiation of UV light (Scheme 8b).^[^ ^31^ ^]^ These evidences suggest that Cl^−^ participates in the water oxidation half‐reaction via a stepwise process where oxidation of Cl^−^ into HClO is followed by the decomposition of HClO to O_2_. The activation energy for producing the HClO intermediate in Cl^−^‐triggered pathway is possibly lower than that for producing some intermediates in Cl^−^‐free oxidation of water, which is evidently an important reason why Cl^−^ can promote the present reaction.

As stated above, H^+^ had also a remarkably accelerated effect on the hydrogenation reaction. Therefore we attempted to clarify the reason behind this phenomenon. Theoretically, an increase of the H^+^ concentration would result in an acceleration of the HClO decomposition (Scheme 8b),^[^ ^31^ ^]^ which was also confirmed by our experimental results related to the HClO decomposition under different concentration of H^+^. This performance of H^+^ is apparently one of the reasons why an addition of H^+^ has an accelerated effect on the water oxidation half‐reaction.

**Scheme 9:**
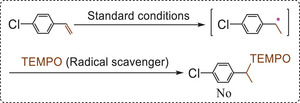
Control experiments related to capturing radicals (for the reaction conditions, see Figure S20 in the Supporting Information).

According to previous literatures,^[^ ^14^, ^17^, ^18^ ^]^ it is possible that the present reaction proceeds through a radical pathway. However, this possibility was ruled out based on our experimental results: When the well‐known radical scavenger TEMPO (2,2,6,6‐tetramethyl‐1‐piperidinyloxy)^[^ ^32^ ^]^ was added into the reaction system, no radical adducts from the reaction between TEMPO and the produced radical intermediate was observed (**Scheme** **9**). Thus, the present reaction should proceed through an ionic pathway based on previous literatures.^[^ ^13^, ^33^ ^]^ It is well known that alkenes can react with H^+^ to give the corresponding carbocations (see Figure S21a, Supporting Information). Theoretically, the produced carbocations have a stronger oxidative capacity than alkenes.^[^ ^21^ ^]^ For example, as shown in Figure S21b,c (Supporting Information), the redox potential for the redox pair related to styrene (−2.71 V) is far lower than that related to the carbocation (0.83 V). Therefore, when the carbocations are adsorbed onto the conduction band of the semiconductor photocatalyst, they can play a role of electron scavengers to promote the water oxidation half‐reaction by accelerating the charge separation/transfer and blocking the electron−hole recombination, which is obviously another important reason why an addition of H^+^ can promote the hydrogenation.

**Figure 9:**
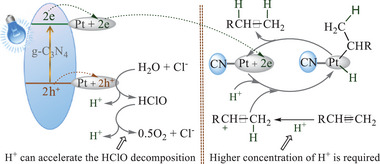
Proposed mechanism for hydrogenation of alkenes under a cooperative catalysis between Pt/g‐C_3_N_4_ and HCl.

On the basis of the above research results and evidence, we propose a main mechanism pathway for the present reaction. As shown in **Figure** **9**, under the irradiation of light, electrons of g‐C_3_N_4_ are excited from the valence band (VB) to the conduction band (CB) to produce electron–hole pairs.^[^ ^34^ ^]^ Then the electrons transfer from the CB to Pt particles,^[^ ^34^ ^]^ which is followed by interactions between Pt and the carbocation as well as H^+^ to give [R‐Pt‐H] intermediate.^[^ ^6^, ^7^, ^8^, ^9^, ^10^, ^11^, ^12^, ^13^, ^14^, ^15^, ^16^, ^17^, ^18^ ^]^ In the meantime, both the carbocation and H^+^ obtain an electron, respectively. Subsequently, [R‐Pt‐H] undergoes the reductive elimination to provide the hydrogenation product.^[^ ^6^, ^7^, ^8^, ^9^, ^10^, ^11^, ^12^, ^13^, ^14^, ^15^, ^16^, ^17^, ^18^ ^]^ In addition, it is possible that some of the alkene molecules directly undergo the hydrogenation half‐reaction without the formation of the carbocation to give the hydrogenation product (see Figure S23 in the Supporting Information).^[^ ^13^, ^33^ ^]^ It is worth noting that H_2_ byproduct can be generated from the reductive elimination of [H‐Pt‐H] intermediate (see Figure S24 in the Supporting Information).

On the other hand, the oxygen evolution half‐reaction also should occur at Pt particles, which has been verified in previous literatures.^[^ ^29^, ^35^ ^]^ First, the holes can migrate from the VB to Pt particles.^[^ ^29^, ^35^ ^]^ Then Cl^−^ and H_2_O is oxidized by the holes to HClO by the assistance of Pt particles.^[^ ^30^ ^]^ Finally, HClO is decomposed to O_2_ and Cl^−^ under the irradiation of UV light.^[^ ^31^ ^]^ As seen from Figure 9, the oxygen evolution half‐reaction can give H^+^, but the produced H^+^ is completely consumed in the hydrogenation half‐reaction. Thus the concentration of H^+^ should be still very low throughout the reaction in the case of not adding H^+^, which is confirmed by our pH analysis results (pH values range from 6.8 to 7.0). The reaction between alkenes and H^+^ is very sluggish in the case of such low concentration of H^+^, which is confirmed by our experimental results: hardly any products from the addition reaction between HI and 4‐chlorostyrene in the case of pH values ranging from 6.8 to 7.0. So an addition of protonic acid is necessary for maintaining higher concentration of H^+^ to enable the carbocation production from the reaction between π‐bond and H^+^.

## 3 Conclusion

In conclusion, we report an example for using water as both the reductant and the proton source to enable the photocatalytic hydrogenation of alkenes, which is in sharp contrast to current photocatalytic methods where a use of pollutive reductants is required to reduce the substrate or the H^+^ of H_2_O.^[^ ^6^, ^7^, ^8^, ^9^, ^10^, ^11^, ^12^, ^13^, ^14^, ^15^, ^16^, ^17^, ^18^ ^]^ In addition, we found an unprecedented cooperative catalysis between Pt/g‐C_3_N_4_ and HCl, in which HCl could effectively accelerate the desired water oxidation half‐reaction and suppress the undesired oxidation of the alkenes. The present method exhibited broad substrate applicability and good functional group tolerance, thus allowed various arylethenes and aliphatic alkenes to undergo the hydrogenation smoothly to give the targeted products in moderate to high yields. Preliminary mechanistic investigation suggests that the hydrogenation half‐reaction works through a process with a pronounced carbocation character. The water oxidation half‐reaction was confirmed to proceed through a Cl^−^‐triggered pathway where oxidation of Cl^−^ into HClO is followed by the decomposition of HClO to O_2_. We believe that this work will guide chemists to use water as a substitute for dangerous hydrogen gas to develop economic and environmental‐friendly procedures for hydrogenation of many organic compounds.

## 4 Experimental Section

### Representative Procedure for Hydrogenation/Deuterization of Various Alkenes

0.5 mmol alkene, 15.00 mg Pt/g‐C_3_N_4_ (0.9 wt% Pt), and 0.3 mL aqueous solution of HCl (37 wt%) were added to a 10 mL glass tube equipped with 3 mL H_2_O or D_2_O (96% D) and 5 mL THF. After the reaction tube was sealed, the reaction was performed for 1.5 h under the irradiation of light (75 W LED, 365 nm wavelength, actual incident light intensity: 0.175 W cm^−2^). Once the reaction time was reached, GC analysis of the mixture provided the GC yields of the product. Then the crude product from another parallel experiment was purified by silica gel chromatography to give the desired product.

## Conflict of Interest

The authors declare no conflict of interest.

## Supporting information



Supporting Information

## Data Availability

The data that support the findings of this study are available in the Supporting Information of this article.
