# Supplementary material for: Photocatalytic Hydrogenation of Alkenes Using Water as Both the Reductant and the Proton Source
Source: Adv Sci (Weinh). 2024 Oct 9;11(44):2406046. doi: 10.1002/advs.202406046 (PMC11600260; doi:10.1002/advs.202406046)
Supplement: Supplementary file 1 — Supporting Information [file ADVS-11-2406046-s001.docx › advs202406046-sup-0001-SuppMat.pdf]

Supporting Information

Photocatalytic Hydrogenation of Alkenes Using Water as both the Reductant and the Proton Source

Xinzhe Tian, Ming Qiu, Wankai An and Yun-Lai Ren*

College of Science, Henan Agricultural University, Zhengzhou, Henan 450002, P.R. China.

E-mail: renyunlai@126.com

Table of Contents

1. General……………...…………………...…...……………………………………...………......S1

1.1 Chemicals…………….…………………….............………………………………….….......…...S1

1.2 Instruments…………………….……………………………….……….....................…........S2

2. Experimental procedure for preparation of catalysts……………………………………...…......S2

2.1 Procedure for preparation of g-C_3N_4…………………………….……………..…...…...S2

2.2 Procedure for preparation of Pt/g-C_3N_4 (x wt% Pt)...….S2

2.3 Procedure for preparation of Ni, Co, Cu, Fe or Pt-loaded g-C_3N_4.........................….S3

3. Characterization of Pt/g-C_3N_4 (0.9 wt% Pt)……………………...………………………….…....S3

4. Experimental procedures and results related to the hydrogenation under different conditions...S4

4.1 Hydrogenation using various cocatalysts……………………………...………...……...S4

4.2 Hydrogenation using various catalysts…………………………………………...……...S5

4.3 Effect of the water loading on the hydrogenation…………………...……………..……...S5

4.4 Hydrogenation using various solvents…………………………………………...……...S6

4.5 Hydrogenation under different wavelengths of light…………...…………………..……...S6

5. Hydrogenation/deuterization of various alkenes…………………………………..…..……...S7

6. Catalytic recycling test of Pt/g-C_3N_4 (0.9 wt% Pt) ……………………………...…..……...S7

6.1 Procedure for catalytic recycling test of Pt/g-C_3N_4 (0.9 wt% Pt) ………………...…..……...S7

6.2 Characterization of the Pt/g-C_3N_4 catalyst (0.9 wt% Pt) before and after two recycles.….......S8

7. Procedure for investigation on who is the electron donor………………...………..…..……..S11

8. Procedure for investigation on necessity for using Pt/g-C_3N_4, THF and light……..……...….S11

9. Procedure for investigation on necessity for using various cocatalysts…….…………………S12

10. Procedure for rotating disk-ring electrode (RRDE) experiments……………….…..………...S12

11. Experimental procedure for determination of H_2O_2…………………………….……….…...S13

12. Control experiments related to capture of radicals……...………………………..…………...S14

13. Computational Details for Fig. S13……………………………………………..…...……....S14

14. One of the proposed mechanisms for the present hydrogenation………………..…..……...S15

15. Mechanism for the hydrogen evolution and the oxygen evolution………………..…..….....S15

16. Spectroscopic and physical data of the isolated products……….……………..…………...S16

17. References………………………..……………………………………………..…..……...S20

18. ^{1}H NMR and ^{13}C NMR for the products………………………………………...…..……...S22

1 General

1.1 Reaction chemicals

The quality and suppliers of the reagents are listed in Table S1. All the chemicals were obtained from commercial vendors and used without further purification.

Table S1 Quality and suppliers of chemicals

Entry	Compound	Purity	Supplier
1	$PtCl_4$	98%	Shanghai Haohong Scientific Co.,Ltd.
2	Melamine	99.9%	Shanghai Haohong Scientific Co.,Ltd.
3	1,3,5-triazinane-2,4,6-trione	98%	Shanghai Haohong Scientific Co.,Ltd.
4	Hydrochloric acid (37 wt%)	AR	Aladdin Chemistry Co., Ltd.
5	Tetrahydrofuran	99.9%	Aladdin Chemistry Co., Ltd.
6	$H_2{}^{18}O$	95%	Aladdin Chemistry Co., Ltd.
7	D_2O (96% D)	96%	Aladdin Chemistry Co., Ltd.
8	4-Chlorostyrene	98%	Shanghai Haohong Scientific Co.,Ltd.
9	4-Cyanostyrene	98%	Shanghai Haohong Scientific Co.,Ltd.
10	Methyl 4-vinylbenzoate	98%	Shanghai Haohong Scientific Co.,Ltd.
11	4-Vinylbenzoic acid	98%	Shanghai Haohong Scientific Co.,Ltd.
12	4-Aminostyrene	98%	Shanghai Haohong Scientific Co.,Ltd.
13	4-Hydroxystyrene	90%	Shanghai Haohong Scientific Co.,Ltd.
14	3,4-Dimethoxystyrene	98%	Shanghai Haohong Scientific Co.,Ltd.
15	4-Vinylbiphenyl	98%	Shanghai Haohong Scientific Co.,Ltd.
16	2-Vinylbenzofuran	98%	Shanghai Haohong Scientific Co.,Ltd.
17	1-Vinylisoquinoline	97%	Aladdin Chemistry Co., Ltd.
18	2-Vinylnaphthalene	98%	Shanghai Haohong Scientific Co.,Ltd.
19	1-Hexadecene	98%	Shanghai Haohong Scientific Co.,Ltd.
20	1-Octadecene	98%	Shanghai Haohong Scientific Co.,Ltd.
21	10-Undecen-1-ol	98%	Shanghai Haohong Scientific Co.,Ltd.
23	13-Tetradecenoic acid	95%	Bide Pharmatech Ltd
25	2-Allylisoindoline-1,3-dione	97%	Shanghai Haohong Scientific Co.,Ltd.
29	2-Hexadecene	98%	Aladdin Chemistry Co., Ltd.

1.2 Instrumtents

^{1}H-NMR and ^{13}C-NMR spectra were recorded on a Bruker 500 MHz instrument with chemical shifts reported in ppm relative to the internal standard tetramethylsilane. GC-MS spectra was recorded on an Agilent 6890/5973N gas chromatography-mass spectrometry instrument. Gas chromatography analyses were performed on a Varian CP-3800 instrument with a FID detector and a CP-WAX 57CB FS capillary chromatographic column (25 m × 0.32 mm). The morphology of Pt/g-C_3N_4 was investigated by a FEI talos F200S transmission electron microscopy (TEM). X-ray photoelectron spectroscopy (XPS) analysis was performed on a ESCALAB 250Xi. The UV-Vis absorption spectra were obtained on a JASCO model V-670 spectrometer equipped with an integrating sphere. Cyclic voltammetry curves were conducted on CHI760E Electrochemical Workstation (Shanghai Chen Hua Electrochemical Instrument) by using a three-electrode (a glassy-carbon or ITO working electrode, a saturated calomel reference electrode and a Ag/AgCl wire counter electrode) electrochemical cell.

2. Experimental procedure for preparation of catalysts

2.1 Procedure for preparation of g-C_3N_4: Polymeric carbon nitride (g-C_3N_4) was prepared following previous procedures with some modification.[1,2] Typically, 45 mL water, 619 mg cyanuric acid and 605 mg melamine were added to a flask. After the reaction system was shaken for 24 h to give a milky suspension, the obtained suspension was centrifuged, and then dried at 60 ^{o}C under vacuum. Then the obtained powder was annealed in a crucible at 550 ^{o}C for 4 h under Ar atmosphere, and then cooled to room temperature to provide yellow g-C_3N_4 powder.

2.2 Procedure for preparation of Pt/g-C_3N_4 (x wt% Pt): Pt/g-C_3N_4 was prepared following previous procedures with some modification.[1,2] Typically, after 150 mg g-C_3N_4 and 150 mL ethanol was added to a 250 mL flask, the system was sonicated for 3 h to make g-C_3N_4 to be dispersed in ethanol. Then y mL (y = 2.32, 1.54, 0.77, 0.39 and 0.15, respectively for 2.7 wt%, 1.8 wt%, 0.9 wt%, 0.5 wt% and 0.2 wt% Pt-loaded on g-C_3N_4) solution of $PtCl_4$ in ethanol (0.01mol/L) was added and the mixture was stirred for 10 min. Then 10 mL water was added and the mixture was

refluxed at 90 ^{o}C for 1 h. Subsequently, the reaction mixture was cooled to room temperature. The precipitation was collected, washed with ethanol, dried at 60 ^{o}C under reduced pressure to give Pt/g-C_3N_4 (2.7 wt%, 1.8 wt%, 0.9 wt%, 0.5 wt% or 0.2 wt% Pt). The Pt loading amount was determined by the inductively coupled plasma–mass spectrometry.

2.3 Procedure for preparation of Ni, Co, Cu, Fe or Pd-loaded g-C_3N_4: M/g-C_3N_4 was prepared following previous procedures with some modification.[1,2] Typically, after 150 mg g-C_3N_4 was added to a 250 mL flask equipped with 150 mL ethanol, the system was sonicated for 3 h to make g-C_3N_4 to be dispersed in ethanol. Then x mL solution of metal salt (2.9 mL solution of $NiCl_2 \cdot 6H_2O$, 7.6 mL solution of $CoCl_2$, 2.6 mL solution of $Cu(OAc)_2 \cdot H_2O$, 5.6 mL solution of $FeCl_3$ and 1.5 mL solution of K_2PdCl_6, respectively for Ni/g-C_3N_4, Co/g-C_3N_4, Cu/g-C_3N_4, Fe/g-C_3N_4 and Pd/g-C_3N_4) in ethanol (0.01 mol/L) was added and the mixture was stirred for 10 min. Subsequently, 10 mL of water was added and the mixture was refluxed at 90 ^{o}C for 1 h. Finally, the reaction mixture was cooled to room temperature. The precipitation was collected, washed with ethanol, dried at 60 ^{o}C under reduced pressure to give Ni/g-C_3N_4 (0.9 wt% Ni), Co/g-C_3N_4 (0.9 wt% Co), Cu/g-C_3N_4 (0.9 wt% Cu), Fe/g-C_3N_4 (0.9 wt% Fe) and Pd/g-C_3N_4 (0.9 wt% Pd), respectively. The transition metal loading amount was determined by the inductively coupled plasma-mass spectrometry.

3. Characterization of Pt/g-C_3N_4 (0.9 wt% Pt)

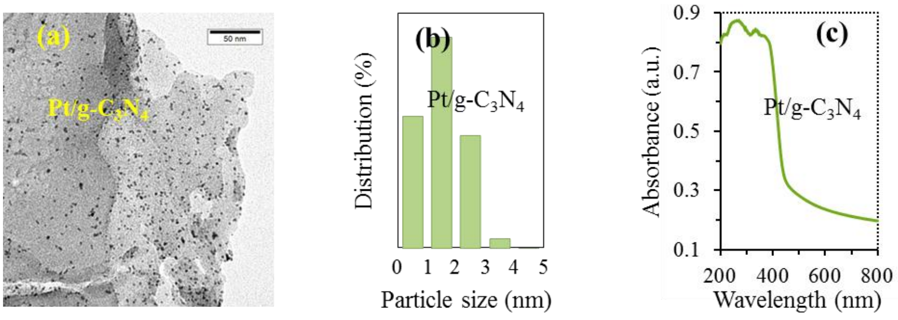

Figure S1. Samples of 0.9 wt% Pt/g-C_3N_4: (a) TEM images. (b) Particle size distribution. (c) UV-visible diffuse reflection spectra.

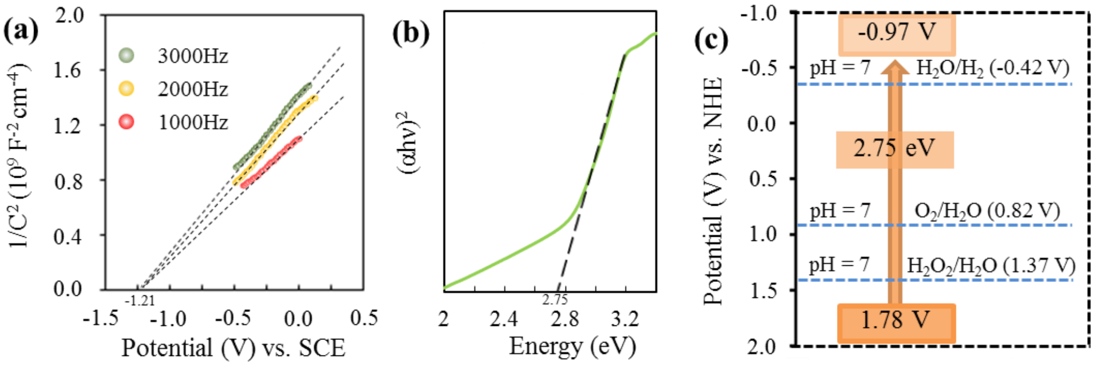

Figure S2. (a) Electrochemical Mott-Schottky plots of Pt/g-C_3N_4 (0.9 wt% Pt) in 0.5 mol/L aqueous solution of Na_2SO_4. (b) Tauc plots of Pt/g-C_3N_4 (0.9 wt% Pt). (c) Energy-band positions. Note: Standard electrode potentials (E^0) of H_2O/H_2, O_2/H_2O and H_2O_2/H_2O are 0.00, 1.23 and 1.78 V vs. NHE, respectively (see Ref. 3). Thus their redox potentials at pH = 7 are calculated to be -0.42, 0.82 V and 1.37 V vs. NHE, respectively, based on Nernst equation.

4. Experimental procedures and results related to the hydrogenation under different conditions

4.1 Hydrogenation using various cocatalysts

0.2 mmol 4-chlorostyrene, 15.00 mg Pt/g-C_3N_4 (0.9 wt% Pt) and cocatalyst were added to a 10 mL quartz glass tube equipped with 3 mL H_2O, 5 mL THF and a magnetic stirrer. Then the reaction tube was sealed and placed in a constant-temperature bath (25 ^oC) to perform the reaction for 1.5 h with magnetic stirring under the light irradiation (light source: 75 W LED, wavelength: 365 nm, actual incident light intensity: 0.175 W/cm^2). Once the reaction time was reached, GC analysis of the mixture provided the GC yields of the product with 1,2,3,4-tetramethylbenzene as an internal standard.

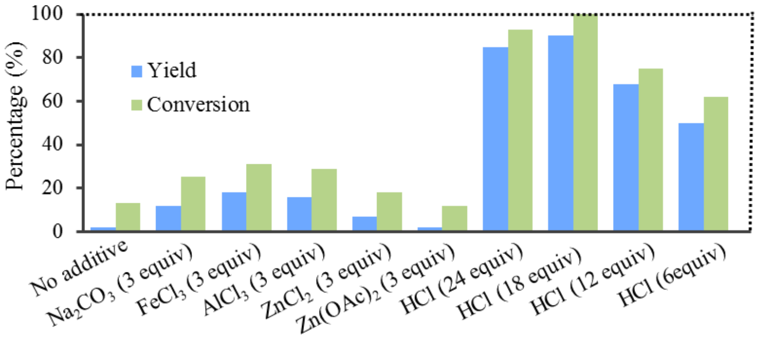

Figure S3. Pt/g-C_3N_4-catalyzed hydrogenation of 4-chlorostyrene in the presence of various cocatalysts under the conditions: 0.2 mmol 4-chlorostyrene, 15 mg Pt/g-C_3N_4 (0.9 wt% Pt), cocatalyst, 3 mL H_2O, 5 mL THF, light (light source: 75 W LED, wavelength: 365 nm, actual incident light intensity: 0.175 W/cm^2).

4.2 Hydrogenation using various catalysts

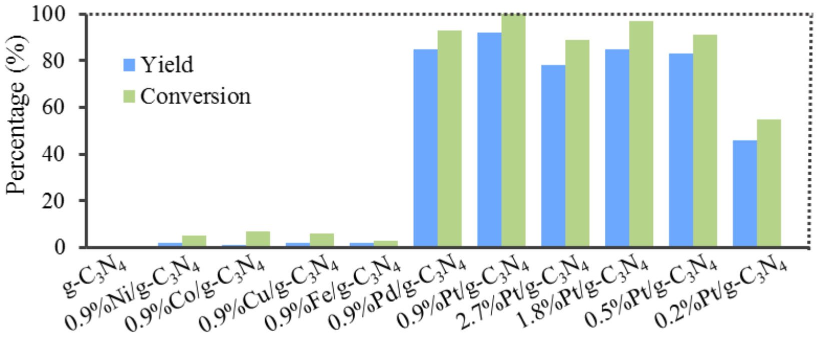

Figure S4. Hydrogenation of 4-chlorostyrene with various catalysts under the conditions: 0.2 mmol 4-chlorostyrene, 15 mg M/g-C_3N_4, 0.3 ml aqueous solution of HCl (37 wt%), 3 mL H_2O, 5 mL THF, light (light source: 75 W LED, wavelength: 365 nm, actual incident light intensity: 0.175 W/cm^2). Note: x%M/g-C_3N_4 means that the actual loading amount of the metal in M/g-C_3N_4 is x wt%. M represents the metals.

0.2 mmol 4-chlorostyrene, 15.00 mg M/g-C_3N_4 (or g-C_3N_4) and 0.3 ml aqueous solution of HCl (37 wt%) were added to a 10 mL quartz glass tube equipped with 3 mL H_2O and 5 mL THF. Then the reaction tube was sealed and placed in a constant-temperature bath (25 ^oC) to perform the reaction for 1.5 h with magnetic stirring under the light irradiation (light source: 75 W LED, wavelength: 365 nm, actual incident light intensity: 0.175 W/cm^2). Once the reaction time was reached, GC analysis of the mixture provided the GC yields of the product with 1,2,3,4-tetramethylbenzene as an internal standard.

4.3 Effect of the water loading on the hydrogenation

Table S2 Effect of the water loading on the hydrogenation[a]

Entry	Water	Conversion	Yield
1	1 mL	75%	72%
2	2 mL	93%	88%
3	3 mL	100%	92%
4	4 mL	100%	90%
5	5 mL	95%	86%
6	6 mL	91%	77%
7	7 mL	82%	62%

[a] Reaction condition: 0.2 mmol 4-chlorostyrene, 15 mg Pt/g-C_3N_4 (0.9 wt% Pt), 0.3 ml aqueous solution of HCl (37 wt%), 8 mL solvent (THF + H_2O), 1.5 h, light irradiation (75 W LED, 365 nm wavelength, actual incident light intensity: 0.175 W/cm^2).

0.2 mmol 4-chlorostyrene, 15.00 mg Pt/g-C_3N_4 (0.9 wt% Pt) and 0.3 ml aqueous solution of HCl (37 wt%) were added to a 10 mL quartz glass tube equipped with 8 mL solvent (THF + H_2O). Then the reaction tube was sealed and placed in a constant-temperature bath (25 ^oC) to perform the reaction for 1.5 h with magnetic stirring and the light irradiation (light source: 75 W LED, wavelength: 365 nm, actual incident light intensity: 0.175 W/cm^2). Once the reaction time was reached, GC analysis of the mixture provided the GC yields of the product with 1,2,3,4-tetramethylbenzene as an internal standard.

4.4 Hydrogenation using various solvents

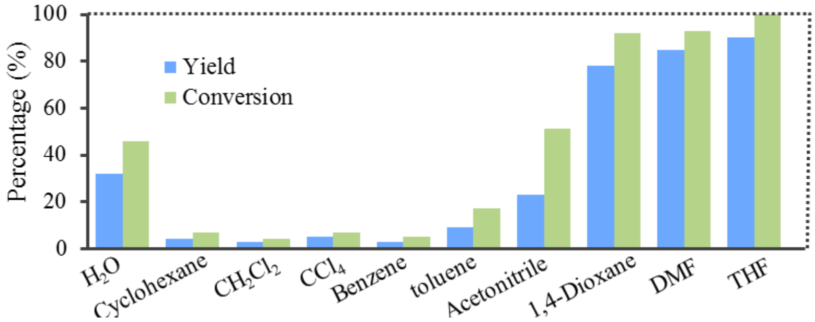

Figure S5. Hydrogenation of 4-chlorostyrene with various solvents under the conditions: 0.2 mmol 4-chlorostyrene, 15 mg Pt/g-C_3N_4 (0.9 wt% Pt), 0.3 ml aqueous solution of HCl (37 wt%), 3 mL H_2O, 5 mL solvent, light (light source: 75 W LED, wavelength: 365 nm, actual incident light intensity: 0.175 W/cm^2).

0.2 mmol 4-chlorostyrene, 15.00 mg Pt/g-C_3N_4 (0.9 wt% Pt) and 0.3 ml aqueous solution of HCl (37 wt%) were added to a 10 mL quartz glass tube equipped with 3 mL H_2O and 5 mL solvent. Then the reaction tube was sealed and placed in a constant-temperature bath (25 ^oC) to perform the reaction for 1.5 h with magnetic stirring and the light irradiation (light source: 75 W LED, wavelength: 365 nm, actual incident light intensity: 0.175 W/cm^2). Once the reaction time was reached, GC analysis of the mixture provided the GC yields of the product with 1,2,3,4-tetramethylbenzene as an internal standard.

4.5 Hydrogenation under different wavelengths of light

0.2 mmol 4-chlorostyrene, 15.00 mg Pt/g-C_3N_4 (0.9 wt% Pt) and 0.3 ml aqueous solution of HCl (37 wt%) were added to a 10 mL quartz glass tube equipped with 3 mL H_2O and 5 mL solvent. Then the reaction tube was sealed and placed in a constant-temperature bath (25 ^oC) to perform the reaction for 1.5 h with magnetic stirring and the light irradiation (light source: 75 W LED, wavelength: 365 nm, actual incident light intensity: 0.175 W/cm^2). Once the reaction time was reached, GC

analysis of the mixture provided the conversion of the substrate and the GC yields of the product with 1,2,3,4-tetramethylbenzene as an internal standard.

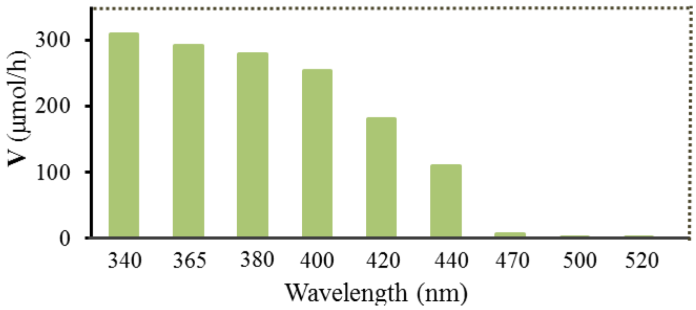

Figure S6 Effect of different wavelength on the reaction

5. Hydrogenation/deuterization of various alkenes

0.2 mmol alkene, 15.00 mg Pt/g-C_3N_4 (0.9 wt% Pt) and 0.3 ml aqueous solution of HCl (37 wt%) were added to a 10 mL quartz glass tube equipped with 3 mL H_2O or D_2O (96% D) and 5 mL THF. Then the reaction tube was sealed and placed in a constant-temperature bath (25 ^oC) to perform the reaction for 1.5 h with magnetic stirring and the light irradiation (light source: 75 W LED, wavelength: 365 nm, actual incident light intensity: 0.175 W/cm^2). Once the reaction time was reached, GC analysis of the mixture provided the GC yields of the product. Then the crude product from another parallel experiment was purified by silica gel chromatography to give the desired product.

6. Catalytic recycling test of Pt/g-C_3N_4 (0.9 wt% Pt)

6.1 Procedure for catalytic recycling test of Pt/g-C_3N_4 (0.9 wt% Pt): 0.2 mmol 4-chlorostyrene, 15.00 mg Pt/g-C_3N_4 (0.9 wt% Pt) and 0.3 ml aqueous solution of HCl (37 wt%) were added to a 10 mL quartz glass tube equipped with 3 mL H_2O and 5 mL THF. Then the reaction tube was sealed and placed in a constant-temperature bath (25 ^oC) to perform the reaction for 1.5 h with magnetic stirring and the light irradiation (light source: 75 W LED, wavelength: 365 nm, actual incident light intensity: 0.175 W/cm^2). Once the reaction time was reached, GC analysis of the mixture provided the GC yields of the hydrogenation product. After reaction, the mixture was filtered to get solid catalyst. The obtained catalyst was washed with a small amount of water and anhydrous ethanol, dried at 55 ^oC in a vacuum oven, then used as the catalyst for next cycle. The Pt/g-C_3N_4 catalyst before and after recycle was characterized by IR, XRD, XPS, SEM, TEM and Mapping analysis.

6.2 Characterization of the Pt/g-C_3N_4 catalyst (0.9 wt% Pt) before and after two recycles

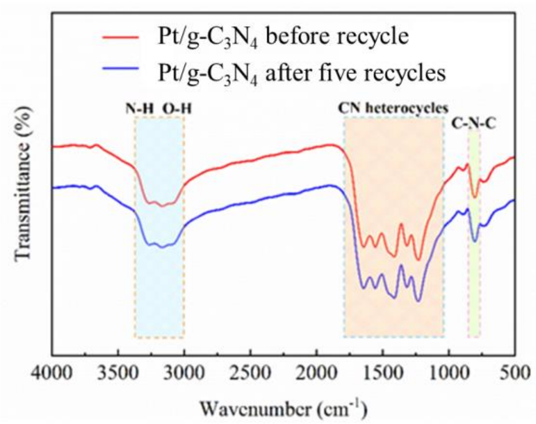

Figure S7 Fourier Transform Infrared Spectrometer (FTIR) of the Pt/g-C_3N_4 catalyst before recycle (red) and after five recycles (blue).

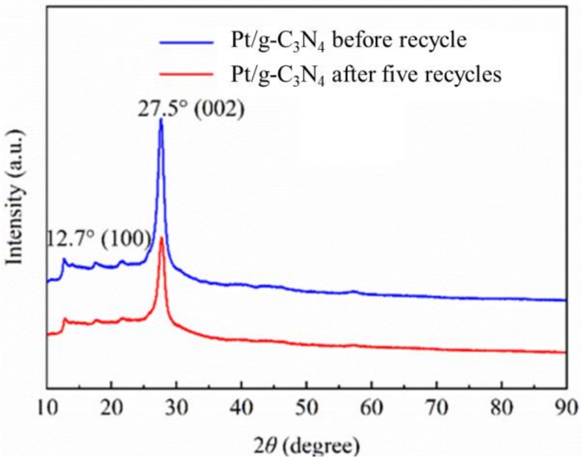

Figure S8 X-ray diffraction (XRD) of the Pt/g-C_3N_4 catalyst before recycle (blue) and after five recycles (red).

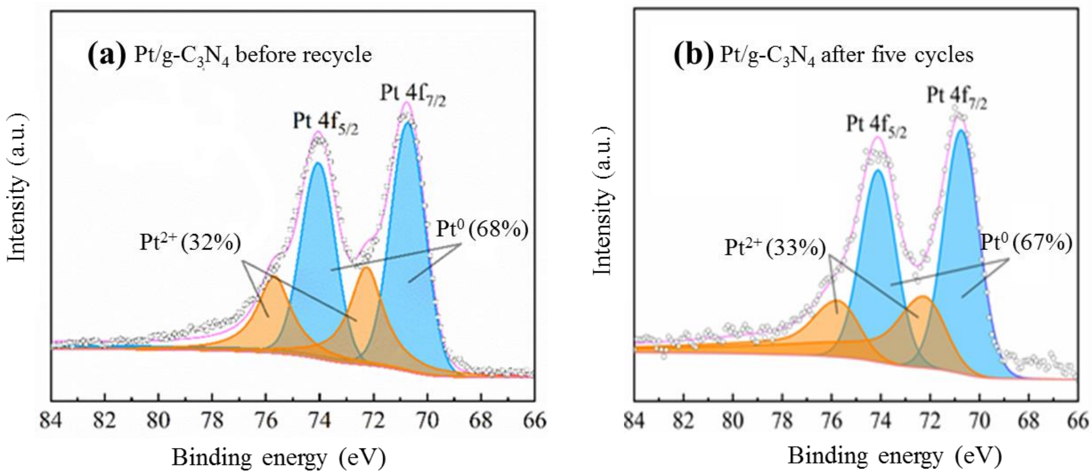

Figure S9 XPS spectra of the Pt/g-C_3N_4 catalyst (a) before recycle, (b) after five recycles.

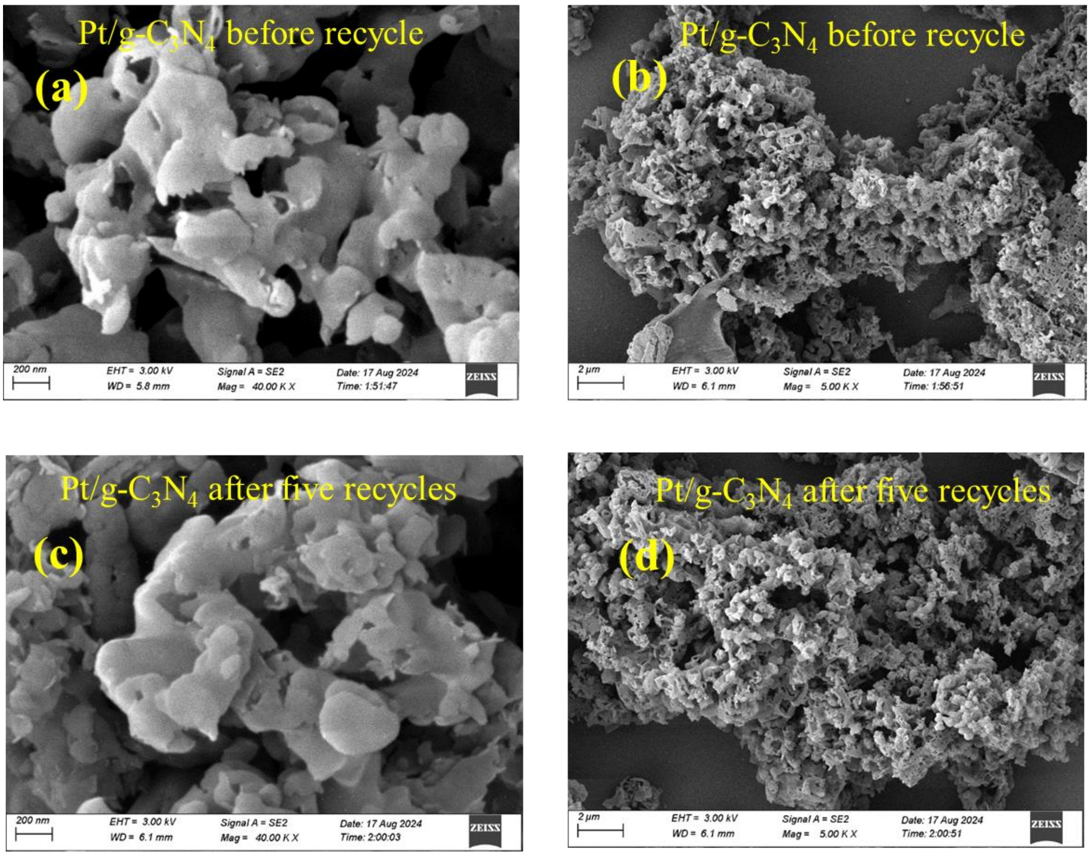

Figure S10 Scanning Electron Microscope (SEM) images of the Pt/g-C_3N_4 catalyst (a) before recycle, (b) before recycle, (c) after five recycles, (d) after five recycles.

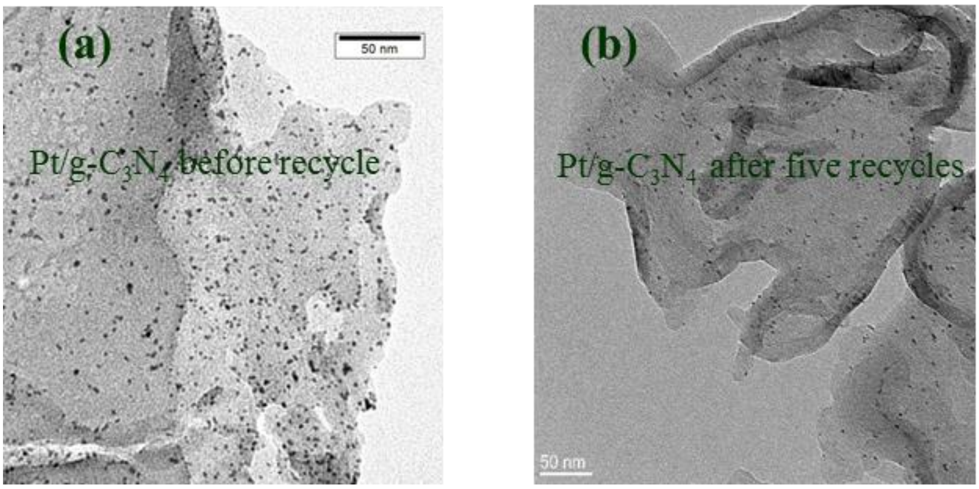

Figure S11 TEM images of the Pt/g-C_3N_4 catalyst (a) before recycle, (b) after five recycles.

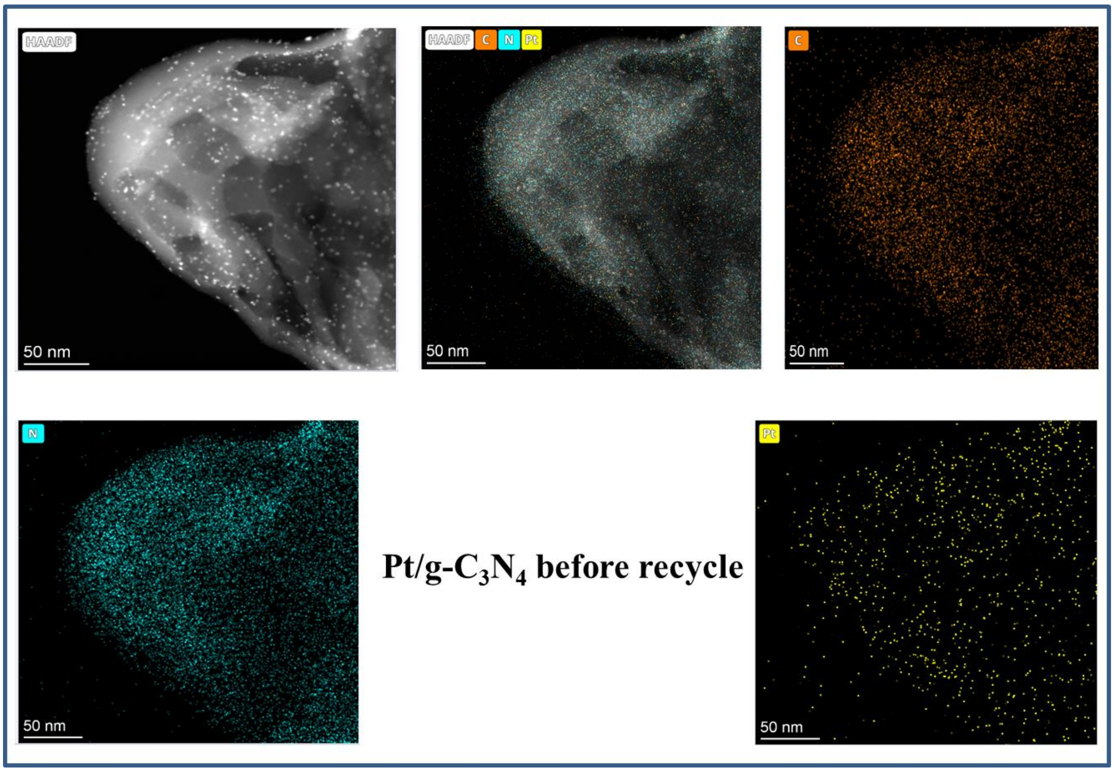

Figure S12 Mapping images of the Pt/g-C_3N_4 catalyst before recycle.

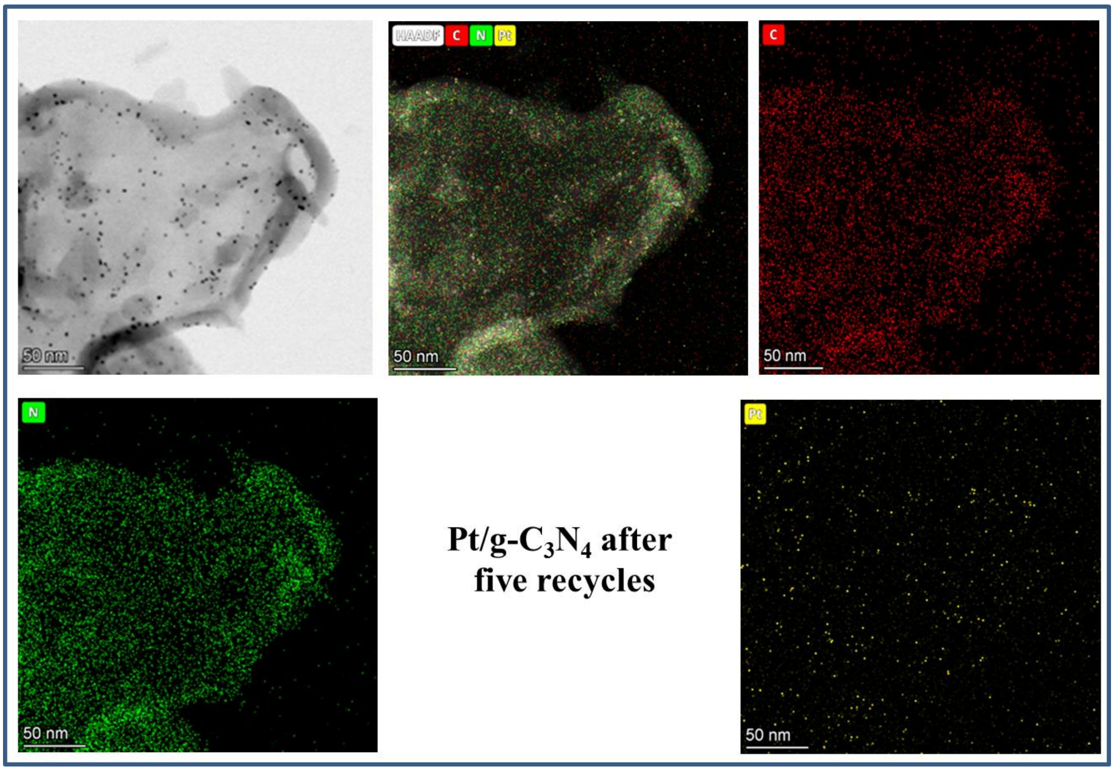

Figure S13 Mapping images of the Pt/g-C_3N_4 catalyst after five recycles.

7. Procedure for investigation on who is the electron donor

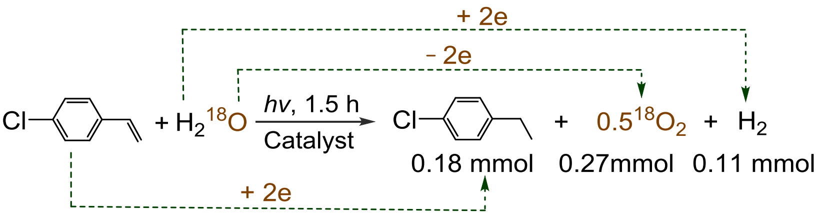

Figure S14 Investigation on who is the electron donor.

0.2 mmol 4-chlorostyrene, 15.00 mg Pt/g-C_3N_4 (0.9 wt% Pt) and 0.3 ml aqueous solution of HCl (37 wt%) were added to a 10 mL quartz glass tube equipped with 3 mL $H_2{}^{18}O$ and 5 mL THF. Then the reaction tube was sealed and placed in a constant-temperature bath (25 ^oC) to perform the reaction for 1.5 h with magnetic stirring and the light irradiation (light source: 75 W LED, wavelength: 365 nm, actual incident light intensity: 0.175 W/cm^2). Once the reaction time was reached, GC analysis of the mixture provided the GC yields of the hydrogenation product. H_2 and $^{18}O_2$ was detected by another Gas chromatography. Formation of $^{18}O_2$ was confirmed by GC-MS.

8. Procedure for investigation on necessity for using Pt/g-C_3N_4, THF and light

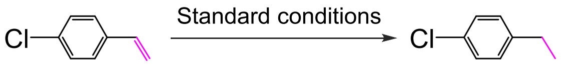

	Condition	Conv.	Yield
**a)**	No Pt/g-C_3N_4	2%	0
**b)**	No THF	39%	33%
**c)**	No light, 40 or 100 ^oC	2-8%	0

Figure S15. Several control experiments for hydrogenation of 4-chlorostyrene.

0.2 mmol 4-chlorostyrene, 15.00 mg Pt/g-C_3N_4 (0.9 wt% Pt) and 0.3 ml aqueous solution of HCl (37 wt%) were added to a 10 mL quartz glass tube equipped with 3 mL H_2O and 5 mL THF. Then the reaction tube was sealed and placed in a constant-temperature bath (25 ^oC) to perform the reaction for 1.5 h with magnetic stirring and the light irradiation (light source: 75 W LED, wavelength: 365 nm, actual incident light intensity: 0.175 W/cm^2). Once the reaction time was reached, GC analysis of the mixture provided the GC yields of the hydrogenation product. Note: a) "No Pt/g-C_3N_4": Pt/g-C_3N_4 was not added. b) "No THF": THF was not added. c) "No light, 40 or 100 ^oC": the reaction was performed under irradiation-free condition and the reaction temperature was 40 or 100 ^oC.

9. Procedure for investigation on necessity for using various cocatalysts

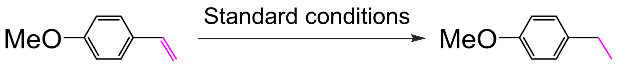

	Cocatalysts	Conv.	Yield
**a)**	-	15%	1%
**b)**	HCl	100%	78%
**c)**	H_2SO_4	43%	39%
**d)**	NaCl	27%	25%
**e)**	KCl	23%	21%

Figure S16. Effect of different cocatalyst on the reaction.

0.2 mmol 4-methoxystyrene, 15.00 mg Pt/g-C_3N_4 (0.9 wt% Pt) and cocatalyst were added to a 10 mL quartz glass tube equipped with 3 mL H_2O and 5 mL THF. Then the reaction tube was sealed and placed in a constant-temperature bath (25 ^oC) to perform the reaction for 1.5 h with magnetic stirring and the light irradiation (light source: 75 W LED, wavelength: 365 nm, actual incident light intensity: 0.175 W/cm^2). Once the reaction time was reached, GC analysis of the mixture provided the GC yields of the hydrogenation product. Note: a) no cocatalyst was added in the case of Figure S16a. b) 0.3 ml aqueous solution of HCl (37 wt%) as the cocatalyst was added in the case of Figure S16b. c) 3.6 mmol NaCl or KCl as the cocatalyst was added in the case of Figure S16d and 16e).

10. Procedure for rotating disk-ring electrode (RRDE) experiments

Table S3 Results from RRDE experiments

Light intensity (mW cm^{-2})	I_{disk} (μA)	I_{ring} (μA)	n[a]
0	0	0	0
2.63	0.58	0.022	3.55
5.61	0.82	0.033	3.53
8.37	1.47	0.05	3.59
12.05	1.81	0.072	3.53

[a] The electron transfer numbers (n) were calculated from equation: $n = 4I_{disk}/(I_{disk} + I_{Ring}/N)$ where N is the RRDE collection efficiency, measured to be 0.30.

According to previous literature,[3] The electron transfer number was studied by rotating disk-ring electrodes (RRDE). The Pt/g-C_3N_4 modified bare glassy carbon electrodes at a rotating speed of 1600 rpm in N_2-saturated solution of HCl in

water/THF (V/V = 3:5). The disk potential was set at 0.6 V (vs. SCE) under different light intensity irradiation (λ= 365 nm). The ring potential was set at 0.9 V (vs. SCE). The results from RRDE experiments are shown in Table S3 and Figure S17.

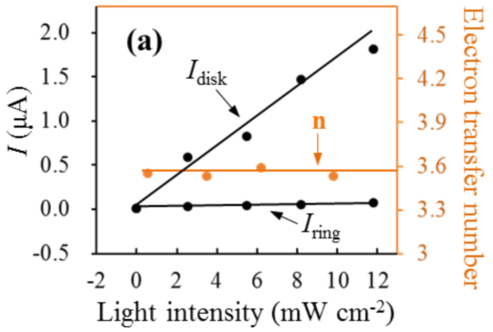

Figure S17. Results from rotating disk-ring electrode (RRDE) experiments

11. Experimental procedure for determination of H_2O_2

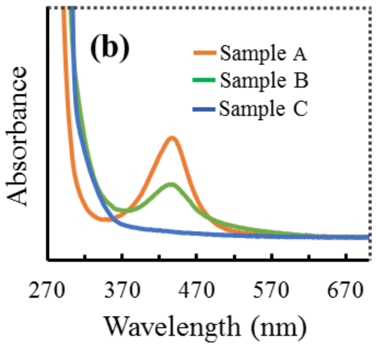

Figure S18. UV-Vis spectroscopy related to the produced H_2O_2 in hydrogenation of 4-chlorostyrene. **Sample A:** 0.4 mmol/L aqueous solution of H_2O_2; **Sample B:** the produced H_2O_2 under standard condition (no HCl and 0.5 h of reaction time); **Sample C:** the produced H_2O_2 under standard conditions (0.5 h of reaction time).

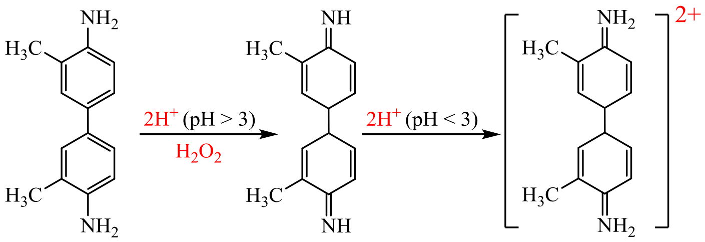

Figure S19. Formation of protonated monomer with absorbance at 436-438 nm.[4]

Experimental procedure for Figure 18 (measurement of H_2O_2): According to previous literature,[4] 0.5 mL volume of 1% o-tolidine in 0.1 mol/L HCl and 2 mL volume of 0.8mg/mL suspension of Pt/g-C_3N_4 in water were added into the sample. After being shaken for 5 min, this mixture turned blue due to the oxidation of *o*-tolidine. Then this mixture was acidified with 1 mol/L HCl (2 mL), and was set for

10 h to give yellow dispersion (for the reaction formulation of the yellow species formation, see Figure S19 and Reference 4). The yellow dispersion was quickly filtered through a 0.22 um membrane filter and the absorption spectrum of the filtrate was immediately recorded with a UV-Vis spectrophotometer. **Note: Sample A:** 0.4 mmol/L aqueous solution of H_2O_2. **Sample B:** the produced H_2O_2 in hydrogenation of 4-chlorostyrene under the following conditions: 0.5 mmol 4-chlorostyrene, 15 mg Pt/g-C_3N_4 (0.9 wt% Pt), 3 mL H_2O, 5 mL THF, 0.5 h, light (light source: 75 W LED, wavelength: 365 nm, actual incident light intensity: 0.175 W/cm^2). **Sample C:** the produced H_2O_2 in hydrogenation of 3,4-dimethoxybromobenzene under the following conditions: 0.2 mmol 4-chlorostyrene, 15 mg Pt/g-C_3N_4 (0.9 wt% Pt), 0.3 ml aqueous solution of HCl (37 wt%), 3 mL H_2O, 5 mL THF, 0.5 h, light (light source: 75 W LED, wavelength: 365 nm, actual incident light intensity: 0.175 W/cm^2).

12. Control experiments related to capture of radicals

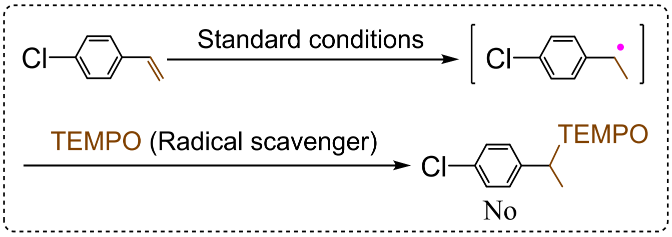

Figure S20. Control experiments related to capture of radicals.

0.2 mmol 4-chlorostyrene, 15.00 mg Pt/g-C_3N_4 (0.9 wt% Pt), 0.3 ml aqueous solution of HCl (37 wt%) and 0.6 mmol TEMPO were added to a 10 mL quartz glass tube equipped with 3 mL H_2O and 5 mL THF. Then the reaction tube was sealed and placed in a constant-temperature bath (25 ^{o}C) to perform the reaction for 1.5 h with magnetic stirring and the light irradiation (light source: 75 W LED, wavelength: 365 nm, actual incident light intensity: 0.175 W/cm^2). Once the reaction time was reached, HPLC-MS (Liquid Chromatograph-Mass Spectrometer) analysis of the mixture provided the GC yields of the hydrogenation product.

13. Computational Details for Figure S21

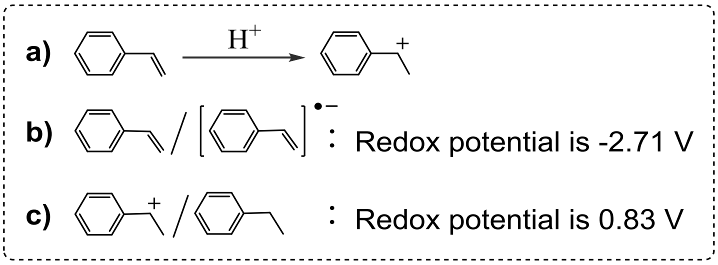

Figure S21. Standard redox potentials related to styrene and carbocation

Standard redox potentials ($E^\ominus$) vs. NHE in Figure S21 were obtained based on the equation ($\Delta_r G_m^\ominus = -nFE^\ominus$, n = 1, F = 96.5 kJ • V^{-1} • mol^{-1}) and the obtained standard molar Gibbs free energy change ($\Delta_r G_m^\ominus$) based on the DFT calculation (Figure S22).

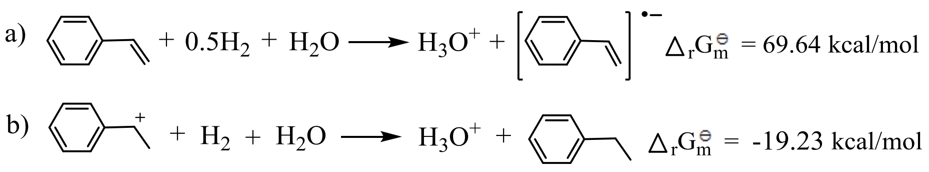

Figure S22. Standard molar Gibbs free energy change related to styrene and carbocation

All DFT calculations performed by Gaussian 16[5] program in the solution phase (solvent = water) with the SMD[6] solvent model. The M06[7] functional with the standard 6-31+G(d,p)[8-10] basis set was used for geometry optimization, frequency calculations and single-point energy calculations. All the optimized stationary points had been identified as minima (zero imaginary frequencies) via the vibrational analysis.

14. One of the proposed mechanisms for the present hydrogenation

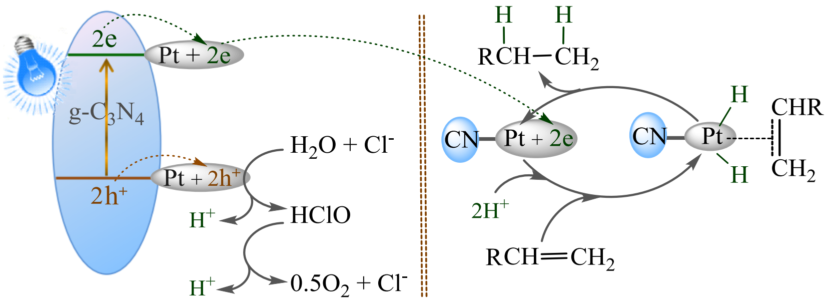

Figure S23. One of the proposed mechanisms for hydrogenation of alkenes under a cooperative catalysis between Pt/g-C_3N_4 and HCl.

15. Mechanism for the hydrogen evolution and the oxygen evolution

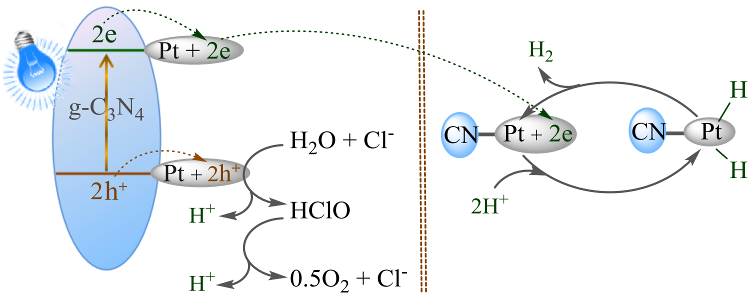

Figure S24. Mechanism for the hydrogen evolution and the oxygen evolution.

16. The spectroscopic and physical data of the isolated products

4-Ethylbenzonitrile[11]

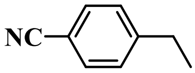

^{1}H NMR (400 MHz, $CDCl_3$): δ 7.57 (d, *J* = 8.3 Hz, 2H), 7.30 (d, *J* = 8.2 Hz, 2H), 2.71 (q, *J* = 7.6 Hz, 2H), 1.25 (t, *J* = 7.5 Hz, 3H); ^{13}C NMR (101 MHz, $CDCl_3$): δ (ppm) = 149.9, 132.2, 128.7, 119.2, 109.5, 29.1, 15.1.

Methyl 4-ethylbenzoate[12]

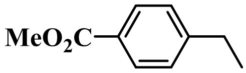

^{1}H NMR (400 MHz, $CDCl_3$): δ (ppm) = 7.96 (d, *J* = 8.3 Hz, 2H), 7.26 (d, *J* = 7.9 Hz, 2H), 3.90 (s, 3H), 2.70 (q, *J* = 7.6 Hz, 2H), 1.25 (t, *J* = 7.6 Hz, 3H); ^{13}C NMR (101 MHz, $CDCl_3$): δ (ppm) = 167.3, 149.8, 129.7, 127.9, 127.7, 52.0, 29.0, 15.3.

4-Ethylbenzoic acid[13]

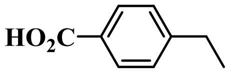

^{1}H NMR (400 MHz, DMSO-d_6): δ (ppm) = 12.81 (s, 1H), 7.85 (d, *J* = 8.2 Hz, 2H), 7.32 (d, *J* = 8.0 Hz, 2H), 2.65 (q, *J* = 7.6 Hz, 2H), 1.18 (t, *J* = 7.6 Hz, 3H); ^{13}C NMR (101 MHz, DMSO-d_6): δ (ppm) = 167.8, 149.6, 130.0, 128.7, 128.4, 28.6, 15.7.

4-Ethylaniline[14]

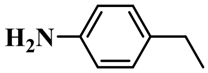

^{1}H NMR (400 MHz, $CDCl_3$): δ (ppm) = 6.88 (d, *J* = 8.4 Hz, 1H), 6.49 (d, *J* = 8.4 Hz, 1H), 3.40 (s, 1H), 2.44 (q, *J* = 7.6 Hz, 1H), 1.09 (t, *J* = 7.6 Hz, 2H); ^{13}C NMR (101 MHz, $CDCl_3$): δ (ppm) = 144.0, 134.2, 128.4, 115.1, 27.8, 15.8.

4-Ethylphenol[15]

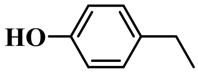

^{1}H NMR (400 MHz, $CDCl_3$): δ (ppm) = 7.07 (d, *J* = 8.0 Hz, 2H), 6.76 (d, *J* = 8.0 Hz, 2H), 4.75 (s, 1H), 2.59 (q, *J* = 8.0 Hz, 2H), 1.21 (t, *J* = 8.0 Hz, 3H).; ^{13}C NMR (101 MHz, $CDCl_3$): δ (ppm) = 152.37, 135.55, 127.89, 114.10, 26.95, 14.85.

4-Ethyl-1,2-dimethoxybenzene[16]

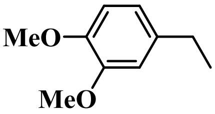

^{1}H NMR (400 MHz, $CDCl_3$): δ (ppm) = 6.80 (d, *J* = 8.6 Hz, 1H), 6.74 (d, *J* = 6.6 Hz, 2H), 3.88 (s, 3H), 3.86 (s, 3H), 2.60 (q, *J* = 7.6 Hz, 2H), 1.22 (d, *J* = 7.6 Hz, 3H); ^{13}C NMR (101 MHz, $CDCl_3$): δ (ppm) = 148.8, 147.0, 137.0, 119.5, 111.3, 111.2, 56.0, 55.8, 28.5, 15.9.

4-Ethyl-1,1'-biphenyl[14]

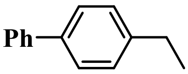

^{1}H NMR (400 MHz, $CDCl_3$): δ (ppm) = 7.59 (d, *J* = 7.0 Hz, 2H), 7.53 (d, *J* = 8.2 Hz, 2H), 7.47 – 7.41 (m, 2H), 7.38 – 7.23 (m, 3H), 2.71 (dd, J_1 = 15.2 Hz, J_2 = 7.6 Hz, 2H), 1.33 – 1.25 (m, 3H); ^{13}C NMR (101 MHz, $CDCl_3$): δ (ppm) = 143.4, 141.2, 138.6, 128.7, 128.3, 127.1, 127.0, 126.9, 28.5, 15.6.

2-Ethylbenzofuran[12]

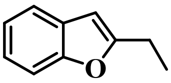

^{1}H NMR (400 MHz, $CDCl_3$): δ (ppm) = 7.48 (d, *J* = 6.9 Hz, 1H), 7.41 (d, *J* = 7.0 Hz, 1H), 7.18 (p, *J* = 7.0 Hz, 2H), 6.37 (s, 1H), 2.79 (q, *J* = 7.5 Hz, 2H), 1.33 (t, *J* = 7.5 Hz, 3H); ^{13}C NMR (101 MHz, $CDCl_3$): δ (ppm) = 161.1, 154.7, 129.1, 123.1, 122.5, 120.3, 110.7, 101.0, 21.9, 11.9.

1-Ethylisoquinoline[17]

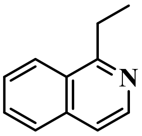

^{1}H NMR (400 MHz, $CDCl_3$): δ (ppm) = 8.43 (d, *J* = 5.8 Hz, 1H), 8.16 (d, *J* = 8.4 Hz, 1H), 7.80 (d, *J* = 8.0 Hz, 1H), 7.69 – 7.63 (m, 1H), 7.58 (t, *J* = 8.3 Hz, 1H), 7.49 (d, *J* = 5.7 Hz, 1H), 3.33 (dd, J_1 = 15.1 Hz, J_2 =7.5 Hz, 2H), 1.45 (t, *J* = 7.6 Hz, 3H); ^{13}C NMR (101 MHz, $CDCl_3$): δ (ppm) = 163.2, 142.0, 136.2, 129.8, 127.4, 127.0, 126.7, 125.3, 119.2, 28.5, 13.6.

2-Ethylnaphthalene[12]

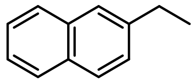

^{1}H NMR (400 MHz, $CDCl_3$): δ (ppm) = 7.89 – 7.78 (m, 1H), 7.67 (s, 1H), 7.53 – 7.42 (m, 1H), 7.40 (d, *J* = 8.2 Hz, 1H), 2.92 – 2.81 (m, 1H), 1.43 – 1.33 (m, 1H); ^{13}C NMR (101 MHz, $CDCl_3$): δ (ppm) = 141.8, 133.8, 132.0, 127.9, 127.7, 127.5, 127.2, 125.9, 125.6, 125.1, 29.1, 15.6.

Hexadecane[18]

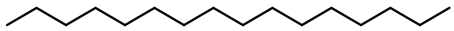

^{1}H NMR (400 MHz, $CDCl_3$): δ (ppm) = 1.26 (s, 28H), 0.88 (t, *J* = 5.6 Hz, 6H); ^{13}C NMR (101 MHz, $CDCl_3$): δ (ppm) = 77.4, 77.0, 76.7, 32.0, 29.7, 29.4, 22.7, 14.1.

Octadecane[18]

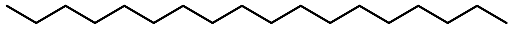

^{1}H NMR (400 MHz, $CDCl_3$): δ (ppm) = 1.25 (s, 32H), 0.88 (t, *J* = 6.3 Hz, 6H); ^{13}C NMR (101 MHz, $CDCl_3$): δ (ppm) = 32.0, 29.7, 29.7, 29.4, 22.7, 14.2.

Undecan-1-ol[19]

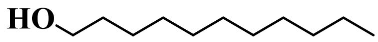

^{1}H NMR (400 MHz, $CDCl_3$): δ (ppm) = 3.62 (t, *J* = 8.0 Hz, 2H), 1.56 (t, *J* = 4.0 Hz, 2H), 1.26 (s, 16H), 0.88 (t, *J* = 4 Hz, 3H).; ^{13}C NMR (101 MHz, $CDCl_3$): δ (ppm) = 62.93, 32.77, 31.92, 29.63, 29.47, 29.35, 25.77, 22.68, 14.08.

Methyl tetradecanoate[20]

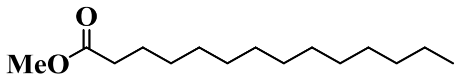

^{1}H NMR (400 MHz, $CDCl_3$): δ (ppm) = 3.66 (s, 3H), 2.29 (t, *J* = 7.5 Hz, 2H), 1.61 (t, *J* = 7.2 Hz ,2H), 1.25 (s, 20H), 0.87 (t, *J* = 6.5 Hz, 3H); ^{13}C NMR (101 MHz, $CDCl_3$): δ (ppm) = 174.4, 51.5, 34.2, 32.0, 29.7, 29.7, 29.6, 29.5, 29.4, 29.3, 29.2, 25.0, 22.7, 14.2.

Tetradecanoic acid[21]

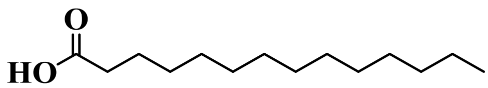

^1H NMR (400 MHz, DMSO-d_6): δ (ppm) = 11.98 (s, 1H), 2.18 (t, J = 7.3 Hz, 2H), 1.48 (t, J = 7.1 Hz, 2H), 1.24 (s, 20H), 0.85 (t, J = 6.4 Hz, 3H); ^{13}C NMR (101 MHz, DMSO-d_6): δ (ppm) = 175.0, 34.1, 31.8, 29.6, 29.5, 29.5, 29.5, 29.4, 29.3, 29.2, 29.1, 25.0, 22.6, 14.4.

Butyl 4-methoxybenzoate[22]

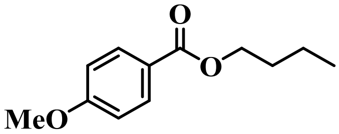

^1H NMR (400 MHz, $CDCl_3$): δ (ppm) = 8.00 (d, J = 8.9 Hz, 2H), 6.92 (d, J = 8.9 Hz, 2H), 4.30 (d, J = 6.6 Hz, 2H), 3.86 (s, 3H), 1.75 (d, J = 6.7 Hz, 2H), 1.47 (d, J = 7.6 Hz, 2H), 0.98 (t, J = 7.4 Hz, 3H); ^{13}C NMR (101 MHz, $CDCl_3$): δ (ppm) =166.5, 163.3, 132.3, 131.6, 123.0, 113.7, 113.6, 64.6, 55.4, 30.8, 19.3, 13.8.

2-Propylisoindoline-1,3-dione[23]

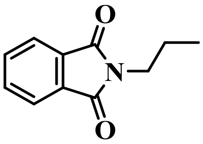

^1H NMR (400 MHz, $CDCl_3$): δ (ppm) =7.83 (dd, J_1 = 5.4 Hz, J_2 =3.1 Hz, 2H), 7.70 (dd, J_1 = 5.4 Hz, J_2 = 3.0 Hz, 2H), 3.70 – 3.59 (m, 2H), 1.69 (d, J = 7.3 Hz, 2H), 1.02 – 0.89 (m, 3H); ^{13}C NMR (101 MHz, $CDCl_3$): δ (ppm) = 168.6, 133.9, 132.2, 123.2, 39.6, 21.9, 11.4.

2-Butylisoindoline-1,3-dione[23]

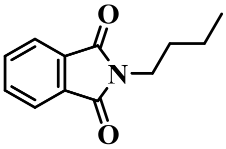

^1H NMR (400 MHz, $CDCl_3$): δ (ppm) = 7.84 (dd, J_1 = 5.5 Hz, J_2 = 2.9 Hz, 2H), 7.71 (dd, J_1 = 5.4 Hz, J_2 = 2.7 Hz, 2H), 3.68 (d, J = 7.4 Hz, 2H), 1.66 (p, J_1 = 7.8 Hz, J_2 = 7.2 Hz, 2H), 1.43–1.32 (m, 2H), 0.95 (t, J = 7.4 Hz, 3H); ^{13}C NMR (101 MHz, $CDCl_3$): δ (ppm) = 168.5, 133.9, 132.2, 123.2, 37.8, 30.7, 20.1, 13.7.

Butoxybenzene[24]

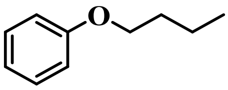

^{1}H NMR (400 MHz, $CDCl_3$): δ (ppm) = 7.80–7.71 (m, 3H), 7.45 (t, *J* = 8.1 Hz ,1H), 7.34 (t, *J* = 8.1 Hz , 1H), 7.16 (d, *J* = 10.7 Hz, 2H), 4.10 (t, *J* = 6.6 Hz, 2H), 1.94–1.79 (m, 2H), 1.56 (h, *J* = 7.4 Hz, 2H), 1.03 (t, *J* = 7.4 Hz, 3H); ^{13}C NMR (101 MHz, $CDCl_3$): δ (ppm) = 157.2, 134.7, 129.4, 128.9, 127.7, 126.8, 126.3, 123.5, 119.1, 106.6, 67.7, 31.4, 19.4, 14.0.

4-Butyl-1,1'-biphenyl[25]

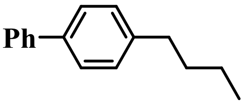

^{1}H NMR (400 MHz, $CDCl_3$): δ (ppm) = 7.58 (d, *J* = 7.0 Hz, 2H), 7.51 (d, *J* = 8.0 Hz, 2H), 7.42 (t, *J* = 7.8 Hz, 2H), 7.32 (t, *J* = 7.4 Hz, 1H), 7.25 (d, *J* = 8.1 Hz, 2H), 2.63 (t, *J* = 7.6 Hz, 2H), 1.75–1.60 (m, 2H), 0.97 (t, *J* = 7.4 Hz, 3H); ^{13}C NMR (101 MHz, $CDCl_3$): δ (ppm) = 141.9, 141.2, 138.6, 129.0, 128.8, 127.1, 127.0, 127.0, 37.8, 24.6, 14.0.

1,2-Diphenylethane-1,2-d_2[26]

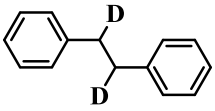

^{1}H NMR (400 MHz, $CDCl_3$): δ (ppm) = 7.32–7.28 (m, 4H), 7.23–7.19 (m, 6H), 2.92 (s, 2H). ^{13}C NMR (101 MHz, $CDCl_3$): δ (ppm) = 142.1, 128.8, 128.7, 126.2, 37.8(m).

17. Reference

[1] X. Fan, Y. L. Yao, Y. S. Xu, L. Yu, C. T. V. Qiu, *ChemCatChem* **2019**, *11*, 2596–2599.

[2] Y. Guo, J. T. Yang, J. Y. Zhuang, H. M. Sun, H. W. Zhang, Y. Y. Yue, H. B. Zhu, X. J. Bao, P. Yuan, *Appl. Catal. A: Gen.* **2020**, *589*, 117312.

[3] J. Liu, Y. Liu, N. Y. Liu, Y. Z. Han, X. Zhang, H. Huang, Y. Lifshitz, S.-T. Lee, J. Zhong, Z. H. Kang, *Science* **2015**, *347*, 970–974.

[4] J. H. Liu, Y. W. Zhang, L. H. Lu, G. Wu, W. Chen, *Chem. Commun.* **2012**, *48*, 8826–8828.

[5] M. J. Frisch, G. W. Trucks, H. B. Schlegel, G. E. Scuseria, M. A. Robb, J. R. Cheeseman, G. Scalmani, V. Barone, G. A. Petersson, H. Nakatsuji, X. Li, M. Caricato, A. V. Marenich, J. Bloino, B. G. Janesko, R. Gomperts, B. Mennucci, H. P. Hratchian, J. V. Ortiz, A. F. Izmaylov, J. L. Sonnenberg, D. Williams-Young, F. Ding, F. Lipparini, F. Egidi, J. Goings, B. Peng, A. Petrone, T. Henderson, D. Ranasinghe, V. G. Zakrzewski, J. Gao, N. Rega, G. Zheng, W. Liang, M. Hada, M. Ehara, K. Toyota, R. Fukuda, J. Hasegawa, M. Ishida, T. Nakajima, Y. Honda, O. Kitao, H. Nakai, T. Vreven, K. Throssell, J. A. Montgomery, Jr., J. E. Peralta, F. Ogliaro, M. J. Bearpark, J. J. Heyd, E. N. Brothers, K. N. Kudin, V. N. Staroverov, T. A. Keith, R. Kobayashi, J. Normand, K. Raghavachari, A. P. Rendell, J. C. Burant, S. S. Iyengar,

J. Tomasi, M. Cossi, J. M. Millam, M. Klene, C. Adamo, R. Cammi, J. W. Ochterski, R. L. Martin, K. Morokuma, O. Farkas, J. B. Foresman, and D. J. Fox. *Gaussian 16, RevisionC.01*, Gaussian, Inc., Wallingford CT, **2016**.

[6] A. V. Marenich, C. J. Cramer, and D. G. Truhlar. "Universal solvation model based on solute electron density and a continuum model of the solvent defined by the bulk dielectric constant and atomic surface tensions". In: J. Phys. Chem. B 113 (2009), pages 6378–96 (cited on pages 299, 301, 311).

[7] Y. Zhao and D. G. Truhlar. "The M06 suite of density functionals for main group thermochemistry, thermochemical kinetics, noncovalent interactions, excited states, and transition elements: two new functionals and systematic testing of four M06-class functionals and 12 other functionals". In: Theor. Chem. Acc. 120 (2008), pages 215–41 (cited on page 97).

[8] G. A. Petersson, A. Bennett, T. G. Tensfeldt, M. A. Al-Laham, W. A. Shirley, and J. Mantzaris. "A complete basis set model chemistry. I. The total energies of closed-shell atoms and hydrides of the first-row atoms". In: J. Chem. Phys. 89 (1988), pages 2193–218 (cited on pages 18, 76, 78).

[9] G. A. Petersson and M. A. Al-Laham. "A complete basis set model chemistry. II. Open-shell systems and the total energies of the first-row atoms". In: J. Chem. Phys. 94 (1991), pages 6081–90 (cited on pages 18, 76, 78).

[10] T. Clark, J. Chandrasekhar, G. W. Spitznagel, and P. v. R. Schleyer. "Efficient diffuse function-augmented basissets for anion calculations. 3. The 3-21+G basis set for 1st-row elements, Li-F". In: *J. Comp. Chem.* 4 (1983), pages 294–301 (cited on page 20).

[11] G. Xue, F.Xie; H. Liang, G. Chen, W. Dai, *Org. Lett.* **2022**, *24*, 5590-5595.

[12] Z. Sun, R.e Ji, J. Wu, J. Zhao, F. Fang, F. Wang, C. Jiang, Z. Liu, *Adv. Synth. Catal.* **2023**, *365*, 476-481.

[13] S. Tang, X. Zhao, L. Yang, B. Li, B. Wang, *Angew. Chem. Int. Ed.* **2022**, *61*, e202212975.

[14] J. Tian, D. Xu, W Sun, *Adv. Syn. & Cat*. **2022**, *364*, 3874-3880.

[15] P. J. Kohlpaintner, L. Marquart, L. J. Gooßen, S. R. Waldvogel, *Eur. J. Org. Chem*. **2023**, *26*, e20230022.

[16] X. Zhou, T. Yu and G. Dong, *J. Am. Chem. Soc.***2022**,*144*,9570-9575.

[17] J. Wang, H. Lu, Y. He, C. Jing, H. Wei, *J. Am. Chem. Soc.***2022**,*144*,22433-22439.

[18] J. Ullrich, B. Breit, *ACS Catal.***2018**,*8*,785-789.

[19] S. Kumari, A. Kulkarni, G. Kumaraswamy, S. S. Gupta, *Chem. Mater.***2013**,*25*,4813-4819.

[20] Y. Zheng, Y. Zhao, S. Tao, X. Li, X. Cheng, G. Jiang, X. Wan, *Eur. J. Org. Chem*. **2021**, *18*, 2713-2718.

[21] T. Iwasaki, K. Higashikawa, V. P. Reddy, W. W. S. Ho,Y. Fujimoto, K. Fukase, J. Terao, H. Kuniyasu,N. Kambe, *Chem. Eur. J.* **2013**, *19*, 2956-2960.

[22] X. Yang, Y. Guo, H. Tong, R. Liu, R. Zhou,*Green Chem.* **2023**, *25*, 1672-1678.

[23] Xi. Liu, J. Qin, W. Dai, Z. Zhu,P. Zhou,Y. Wang,J. Nie, Y. Yang, Z. Zhang, *ACS Catal.* **2022**, *12*, 13300-13311.

[24] C. Matt, F. Kölblin J. Streuff, *Org. Lett.* **2019**, *21*, 6983-6988.

[25] P. Visser, B. L. Feringa, *Chem. Commun.* **2023**, *59*, 5539-5542.

[26] M. Yaghoubi, I. C. Reyes, B. J. *StokesSynOpen* **2024**, *8*, 169–172.

18. ¹H NMR and ¹³C NMR for the products

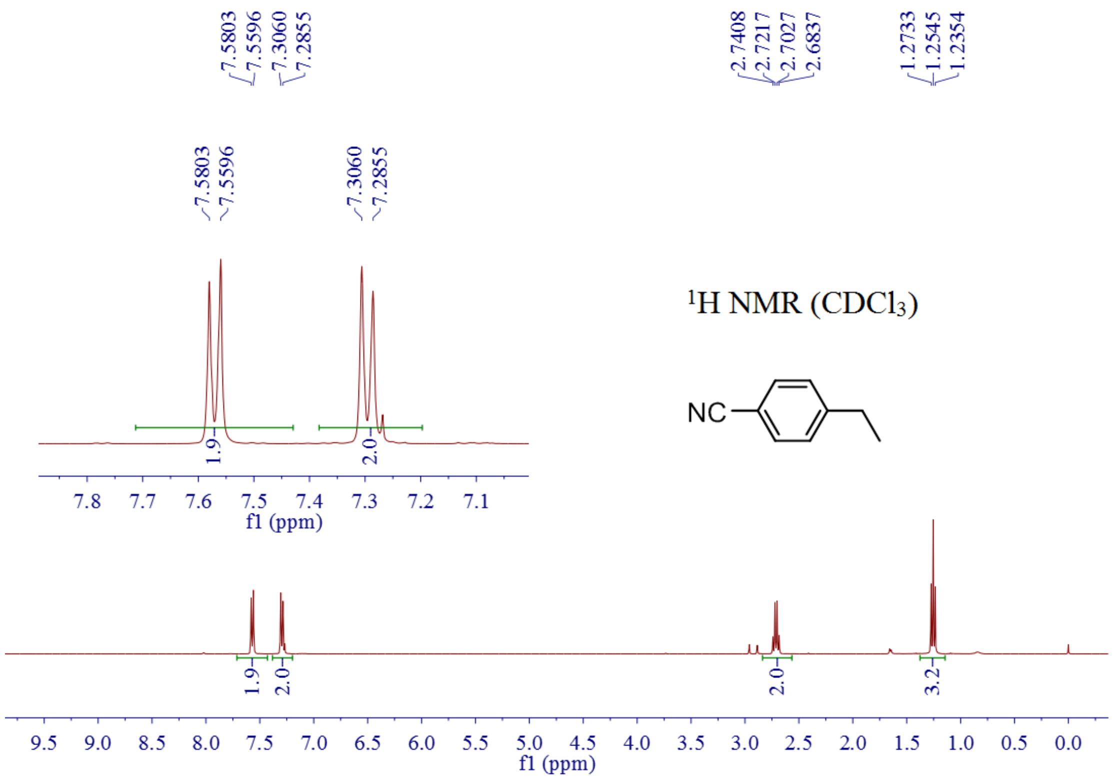

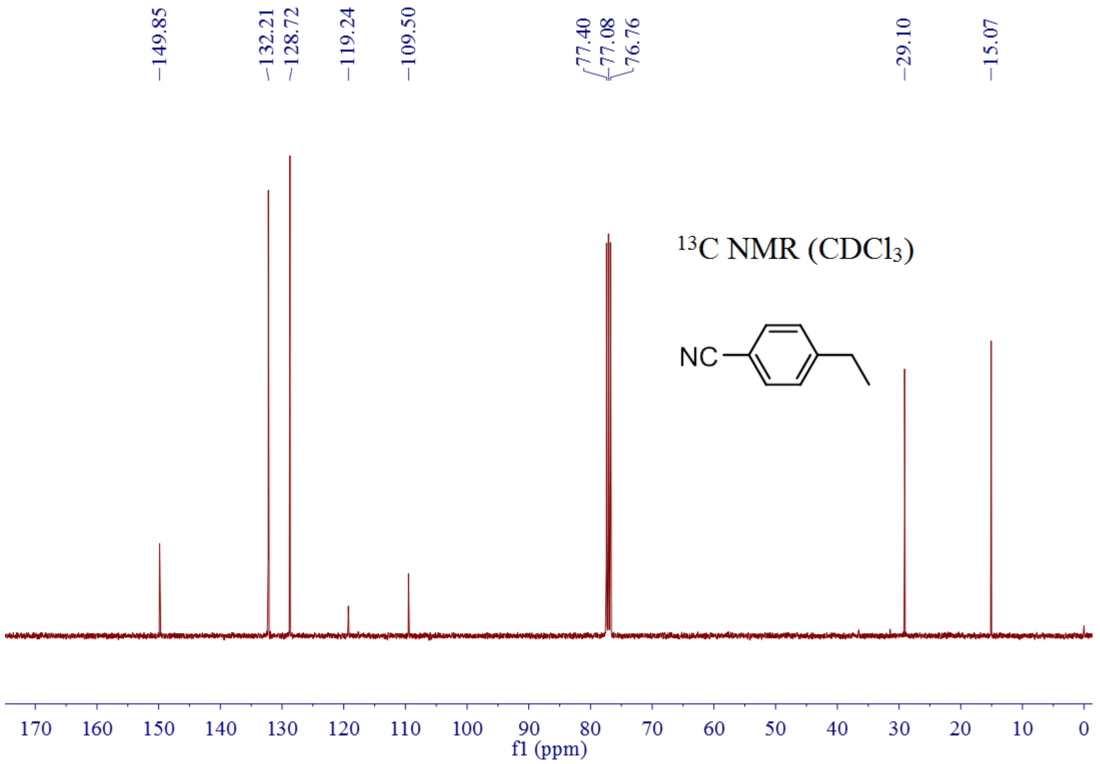

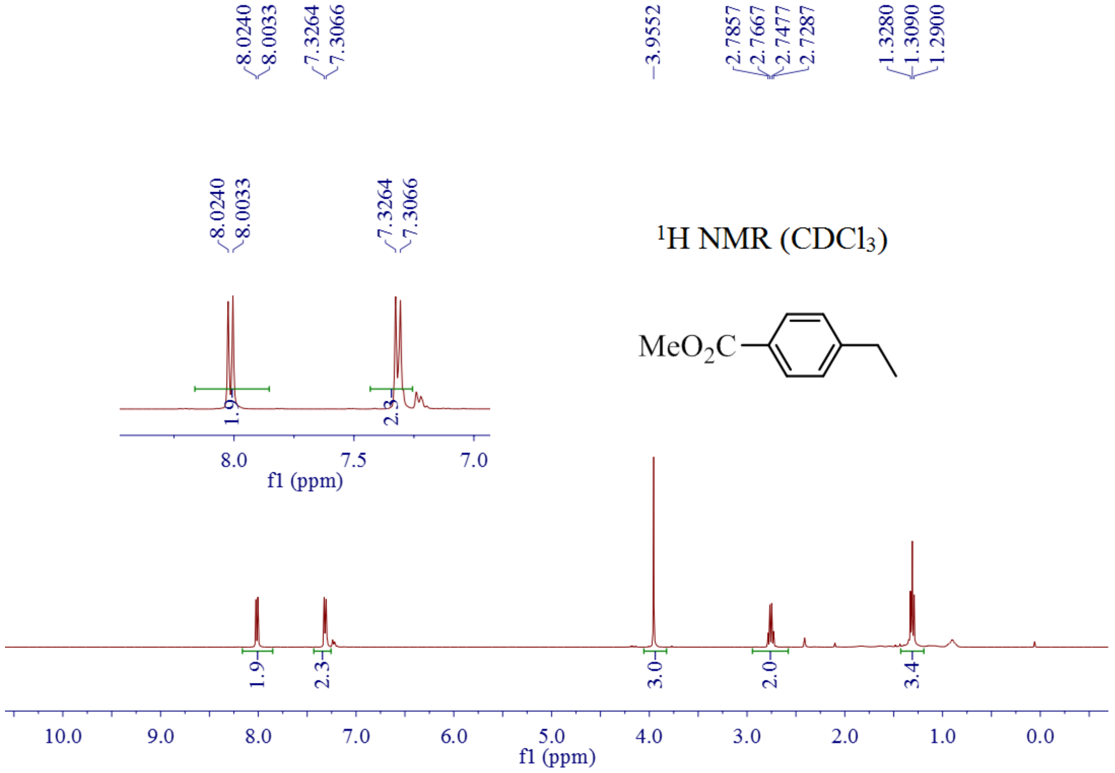
8.0240
8.0033
7.3264
7.3066
3.9552
2.7857
2.7667
2.7477
2.7287
1.3280
1.3090
1.2900
^{1}H NMR ($CDCl_3$)
MeO_2C
1.9
2.3
3.0
2.0
3.4
f1 (ppm)

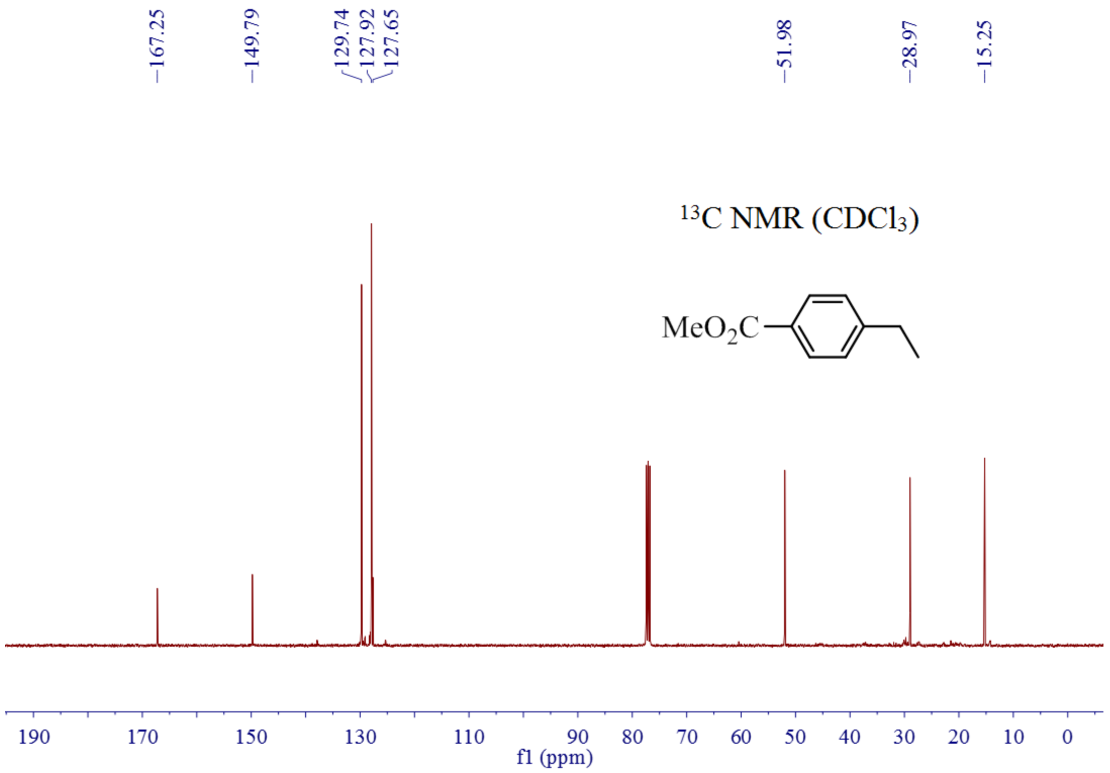
167.25
149.79
129.74
127.92
127.65
51.98
28.97
15.25
^{13}C NMR ($CDCl_3$)
MeO_2C
f1 (ppm)

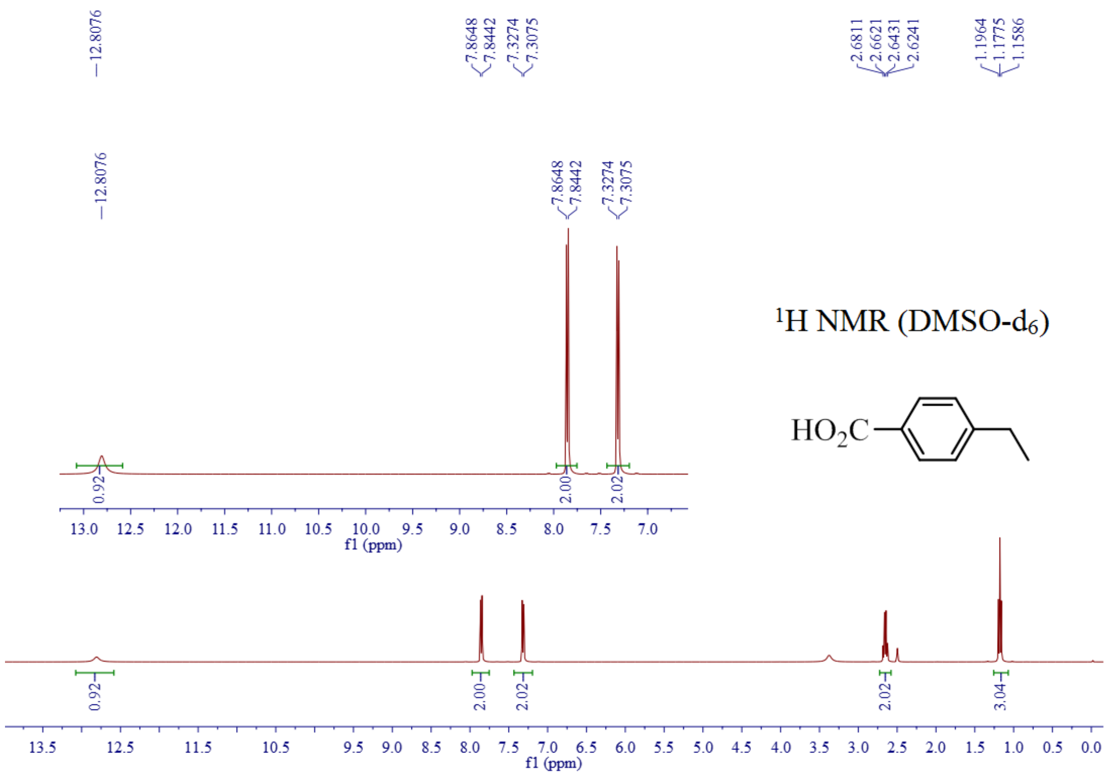

12.8076
7.8648
7.8442
7.3274
7.3075
2.6811
2.6621
2.6431
2.6241
1.1964
1.1775
1.1586
^{1}H NMR (DMSO-d_6)
HO_2C
0.92
2.00
2.02
3.04
f1 (ppm)

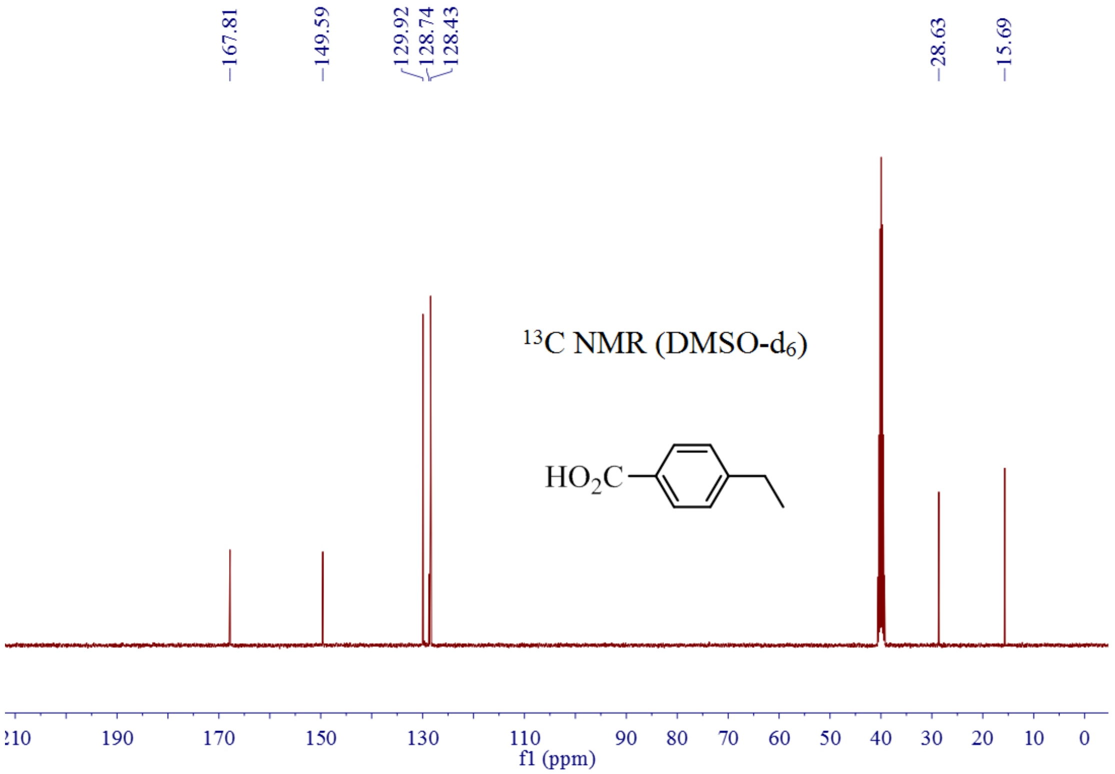

167.81
149.59
129.92
128.74
128.43
28.63
15.69
^{13}C NMR (DMSO-d_6)
HO_2C
f1 (ppm)

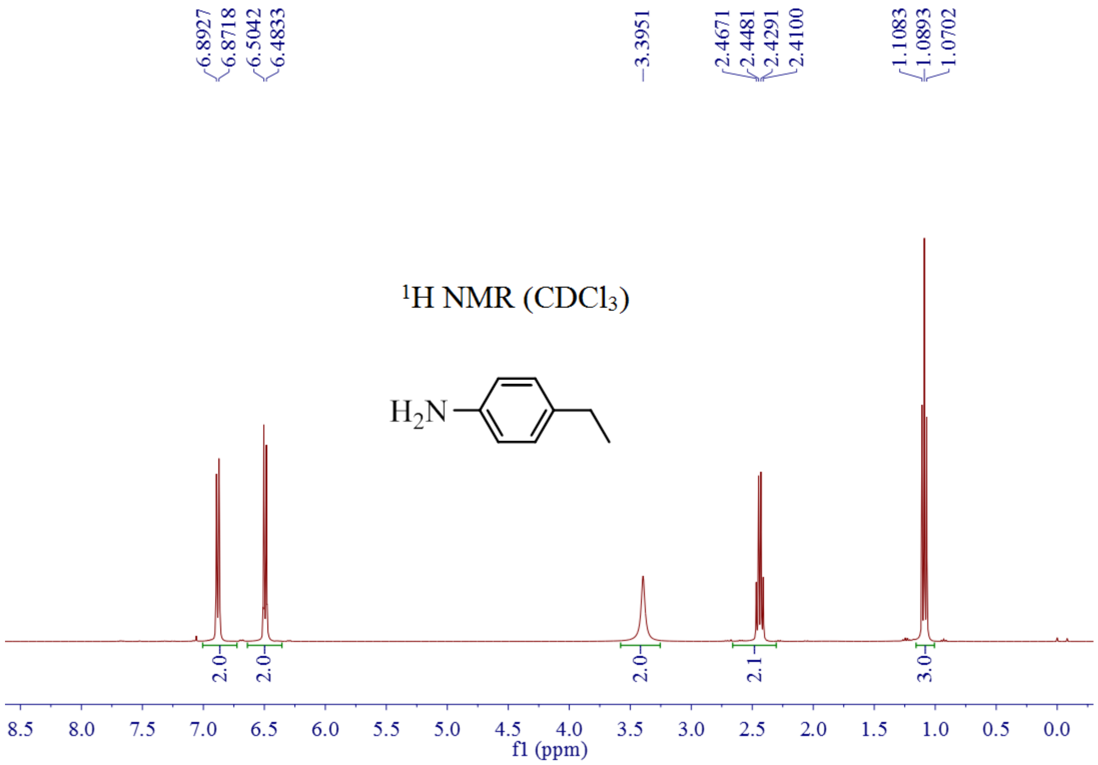
6.8927
6.8718
6.5042
6.4833
3.3951
2.4671
2.4481
2.4291
2.4100
1.1083
1.0893
1.0702
¹H NMR (CDCl3)
H2N
2.0
2.0
2.0
2.1
3.0
8.5 8.0 7.5 7.0 6.5 6.0 5.5 5.0 4.5 4.0 3.5 3.0 2.5 2.0 1.5 1.0 0.5 0.0
f1 (ppm)

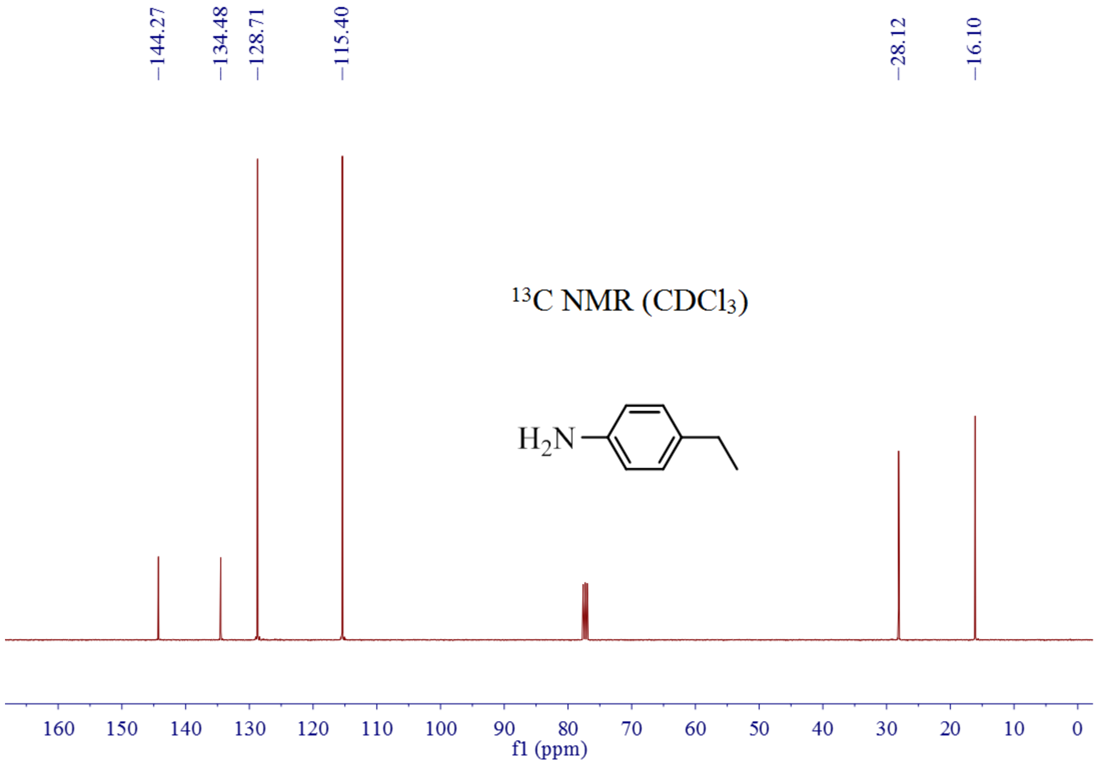
144.27
134.48
128.71
115.40
28.12
16.10
¹³C NMR (CDCl3)
H2N
160 150 140 130 120 110 100 90 80 70 60 50 40 30 20 10 0
f1 (ppm)

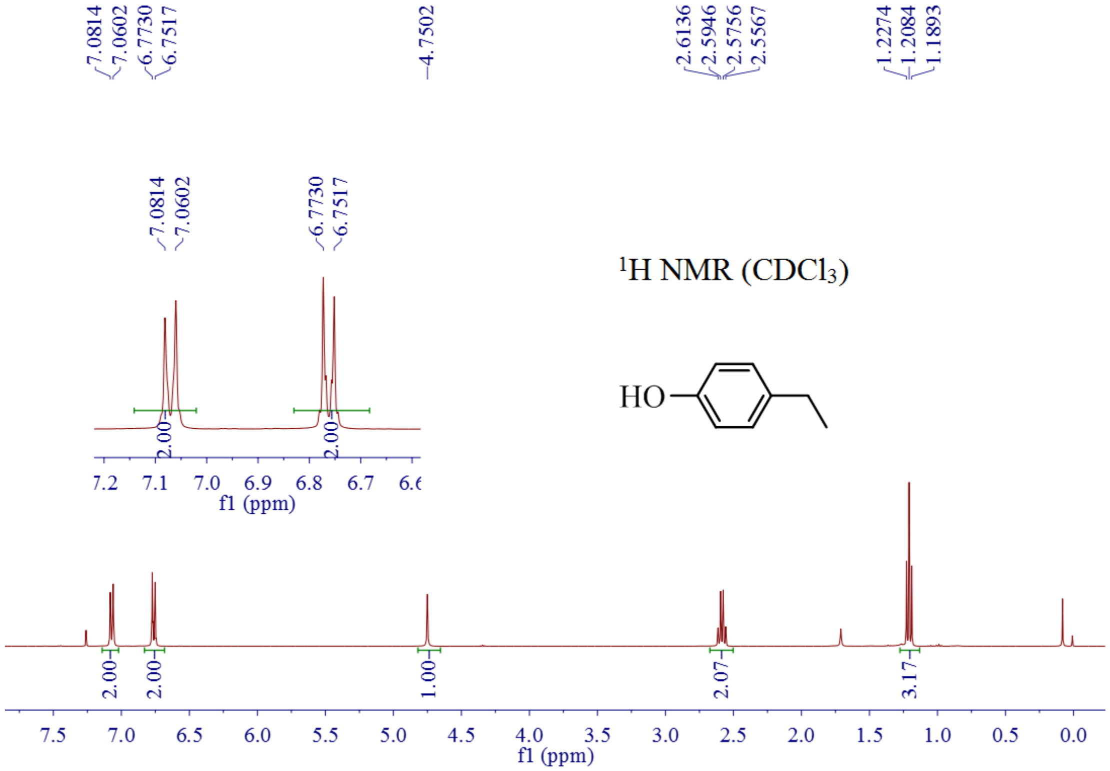
7.0814
7.0602
6.7730
6.7517
4.7502
2.6136
2.5946
2.5756
2.5567
1.2274
1.2084
1.1893
1H NMR (CDCl3)
HO
2.00
2.00
1.00
2.07
3.17
f1 (ppm)

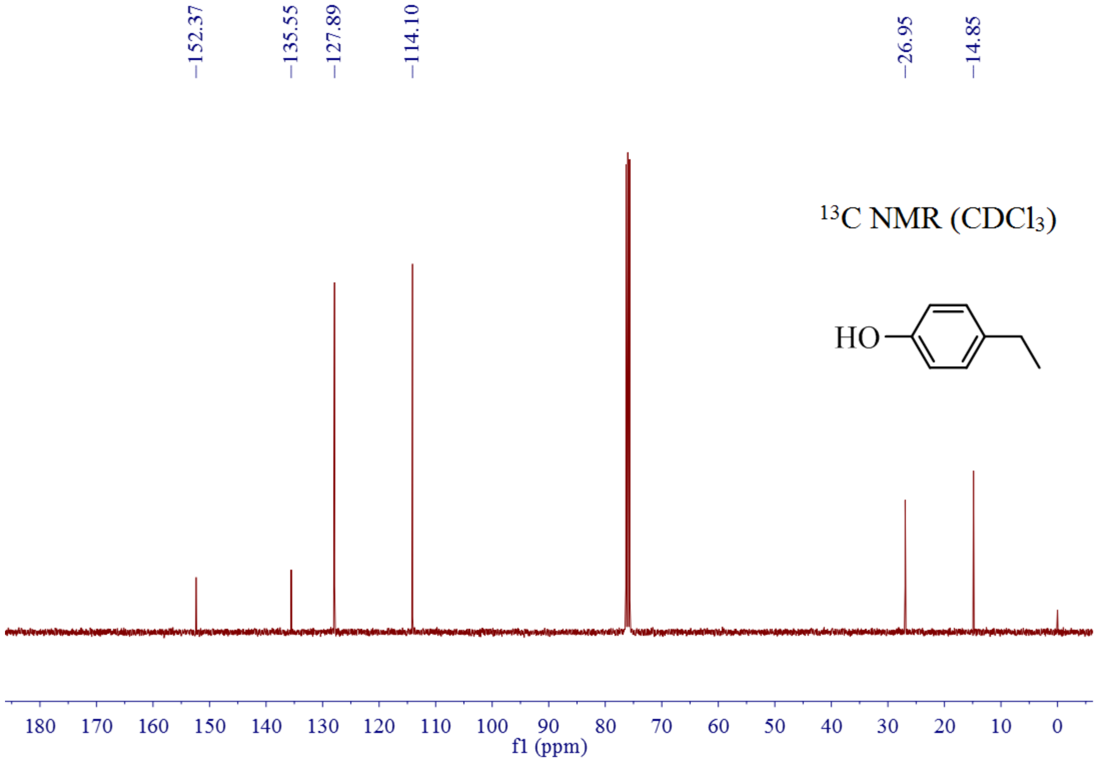
152.37
135.55
127.89
114.10
26.95
14.85
13C NMR (CDCl3)
HO
f1 (ppm)

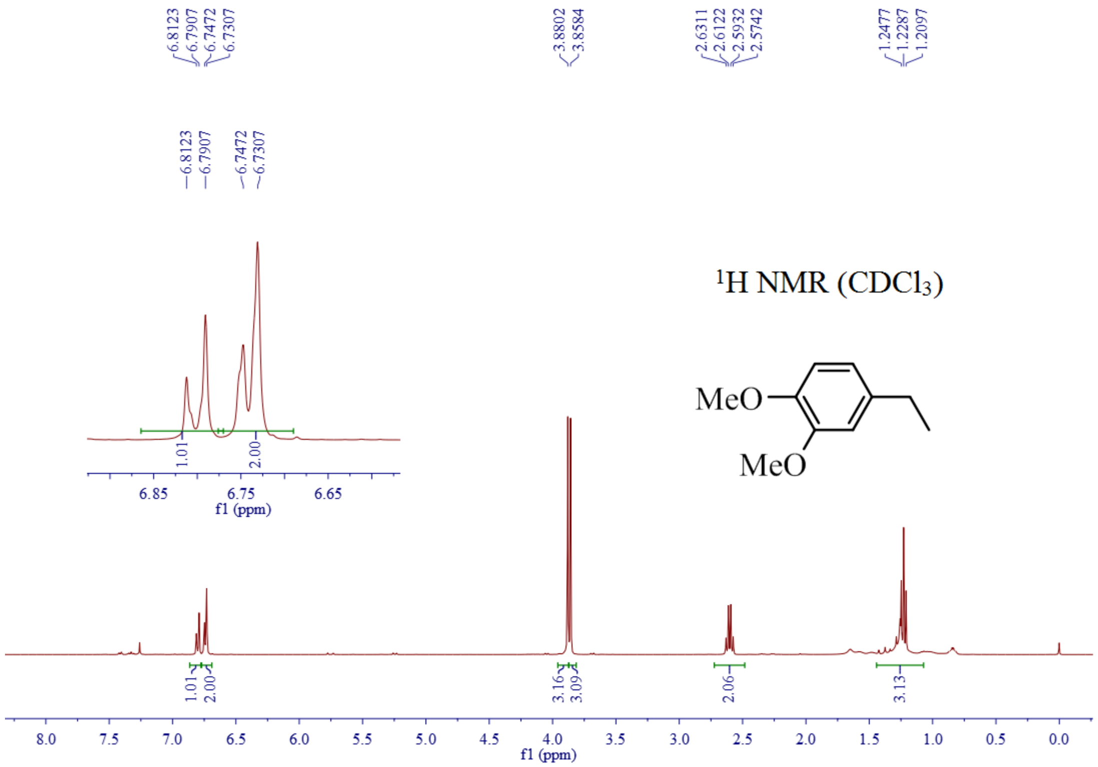
6.8123
6.7907
6.7472
6.7307
3.8802
3.8584
2.6311
2.6122
2.5932
2.5742
1.2477
1.2287
1.2097
^{1}H NMR ($CDCl_3$)
MeO
MeO
1.01
2.00
6.85
6.75
6.65
f1 (ppm)
3.16
3.09
2.06
3.13
8.0 7.5 7.0 6.5 6.0 5.5 5.0 4.5 4.0 3.5 3.0 2.5 2.0 1.5 1.0 0.5 0.0
f1 (ppm)

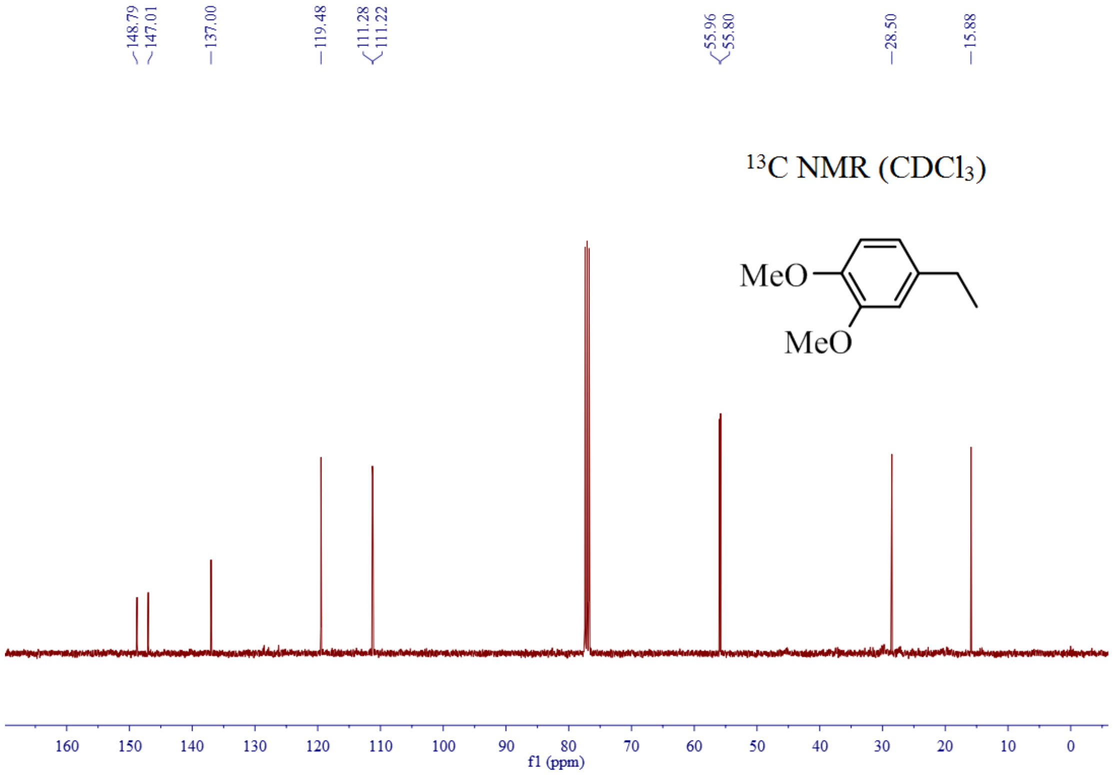
148.79
147.01
137.00
119.48
111.28
111.22
55.96
55.80
28.50
15.88
^{13}C NMR ($CDCl_3$)
MeO
MeO
160 150 140 130 120 110 100 90 80 70 60 50 40 30 20 10 0
f1 (ppm)

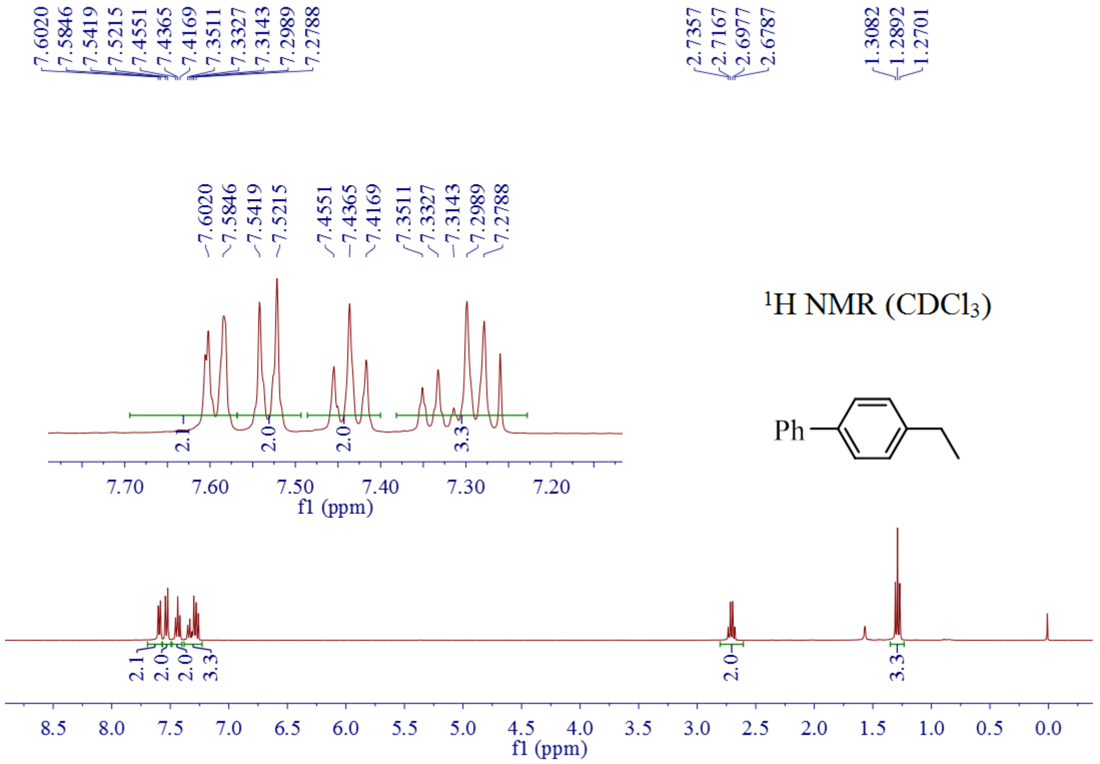
7.6020
7.5846
7.5419
7.5215
7.4551
7.4365
7.4169
7.3511
7.3327
7.3143
7.2989
7.2788
2.7357
2.7167
2.6977
2.6787
1.3082
1.2892
1.2701
1H NMR (CDCl3)
Ph
2.1
2.0
2.0
3.3
7.70
7.60
7.50
7.40
7.30
7.20
f1 (ppm)
8.5
8.0
7.5
7.0
6.5
6.0
5.5
5.0
4.5
4.0
3.5
3.0
2.5
2.0
1.5
1.0
0.5
0.0
f1 (ppm)

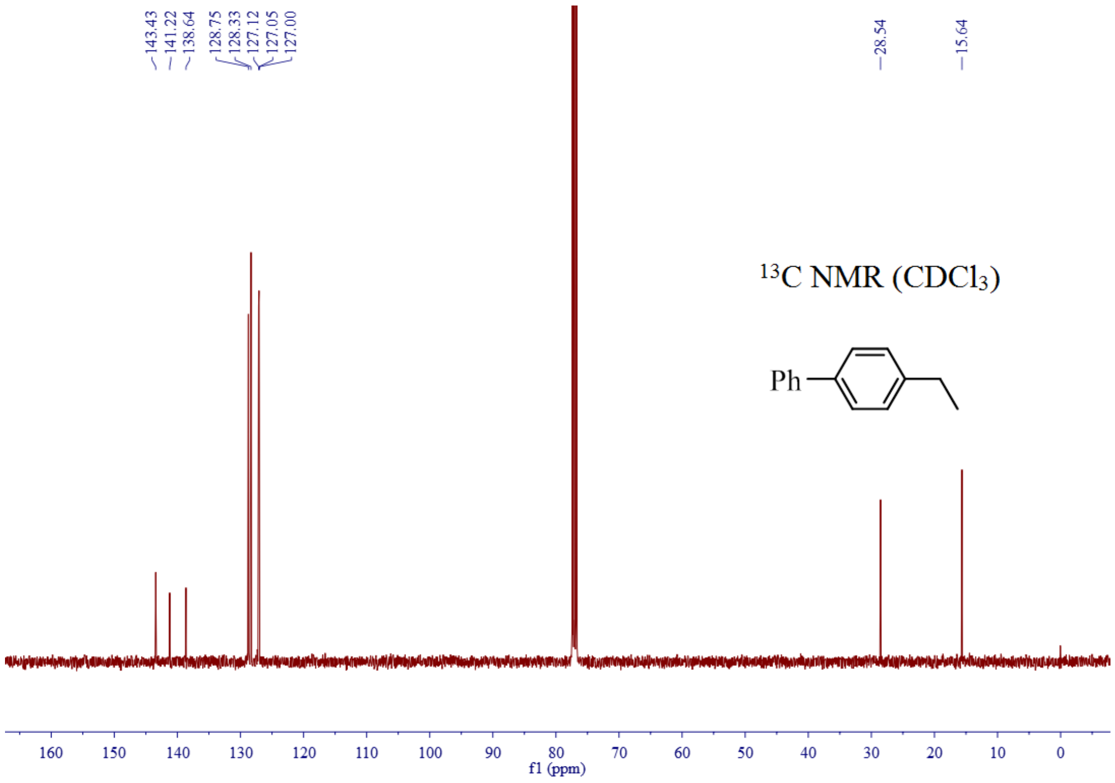
143.43
141.22
138.64
128.75
128.33
127.12
127.05
127.00
28.54
15.64
13C NMR (CDCl3)
Ph
160
150
140
130
120
110
100
90
80
70
60
50
40
30
20
10
0
f1 (ppm)

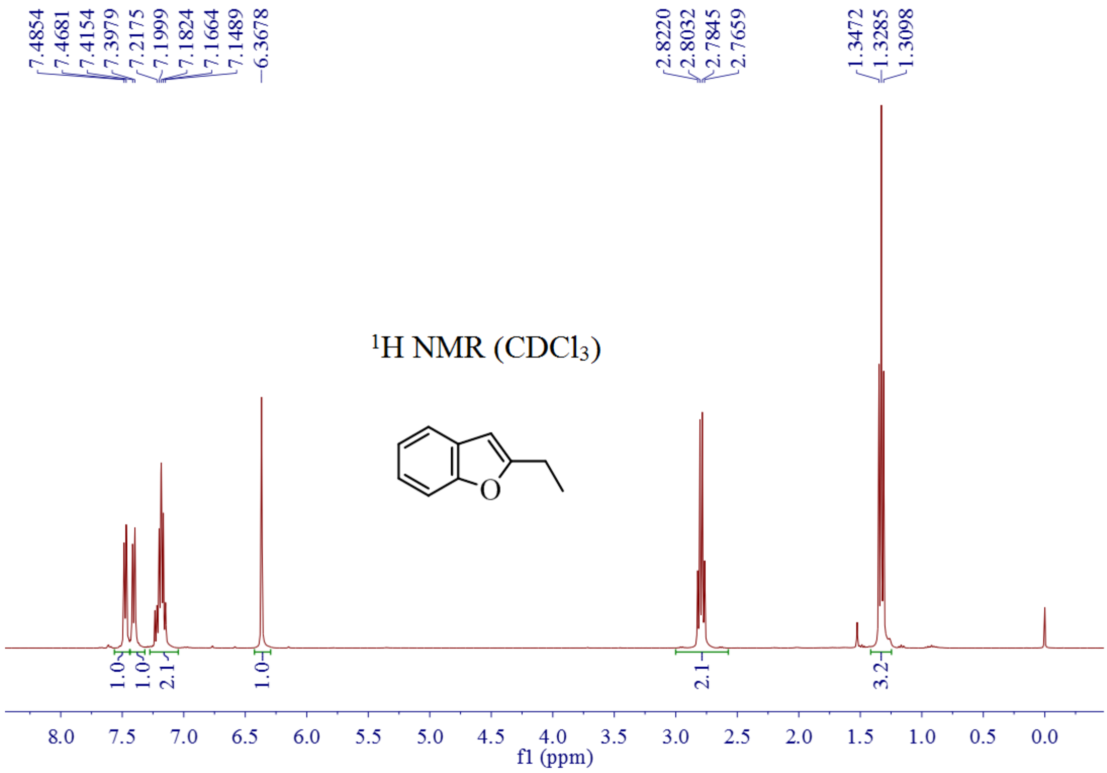
7.4854
7.4681
7.4154
7.3979
7.2175
7.1999
7.1824
7.1664
7.1489
6.3678
2.8220
2.8032
2.7845
2.7659
1.3472
1.3285
1.3098
¹H NMR (CDCl₃)
O
1.0
1.0
2.1
1.0
2.1
3.2
8.0
7.5
7.0
6.5
6.0
5.5
5.0
4.5
4.0
3.5
3.0
2.5
2.0
1.5
1.0
0.5
0.0
f1 (ppm)

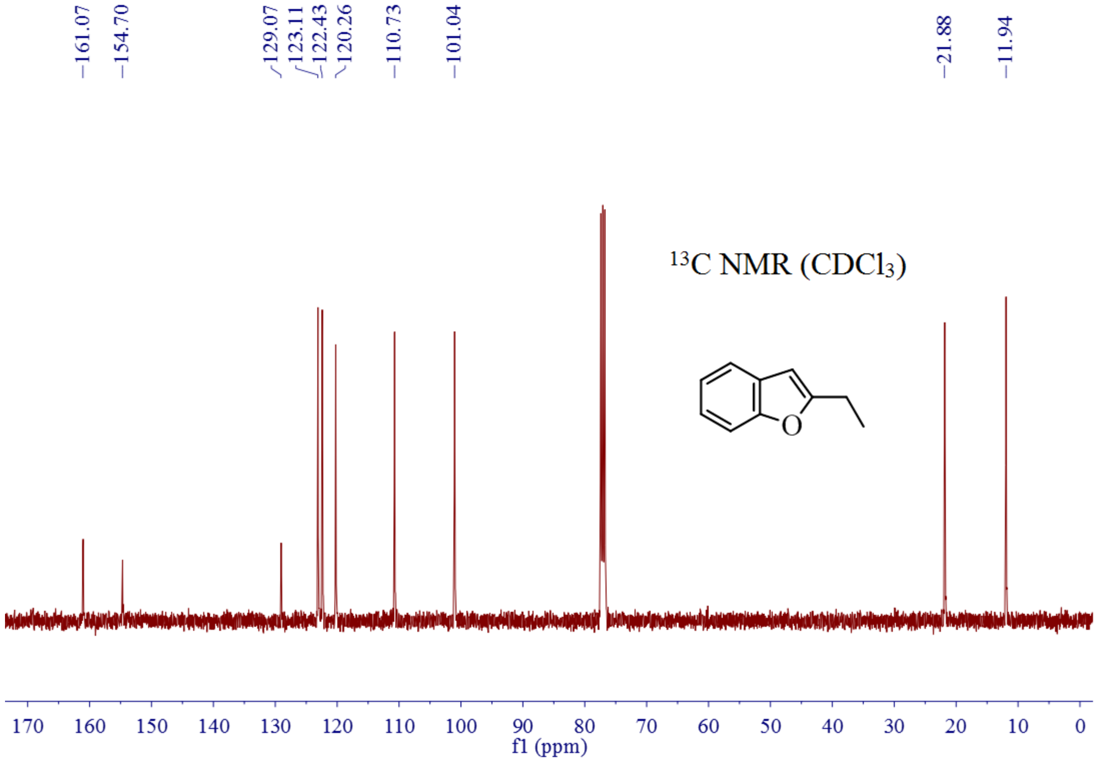
161.07
154.70
129.07
123.11
122.43
120.26
110.73
101.04
21.88
11.94
¹³C NMR (CDCl₃)
O
170
160
150
140
130
120
110
100
90
80
70
60
50
40
30
20
10
0
f1 (ppm)

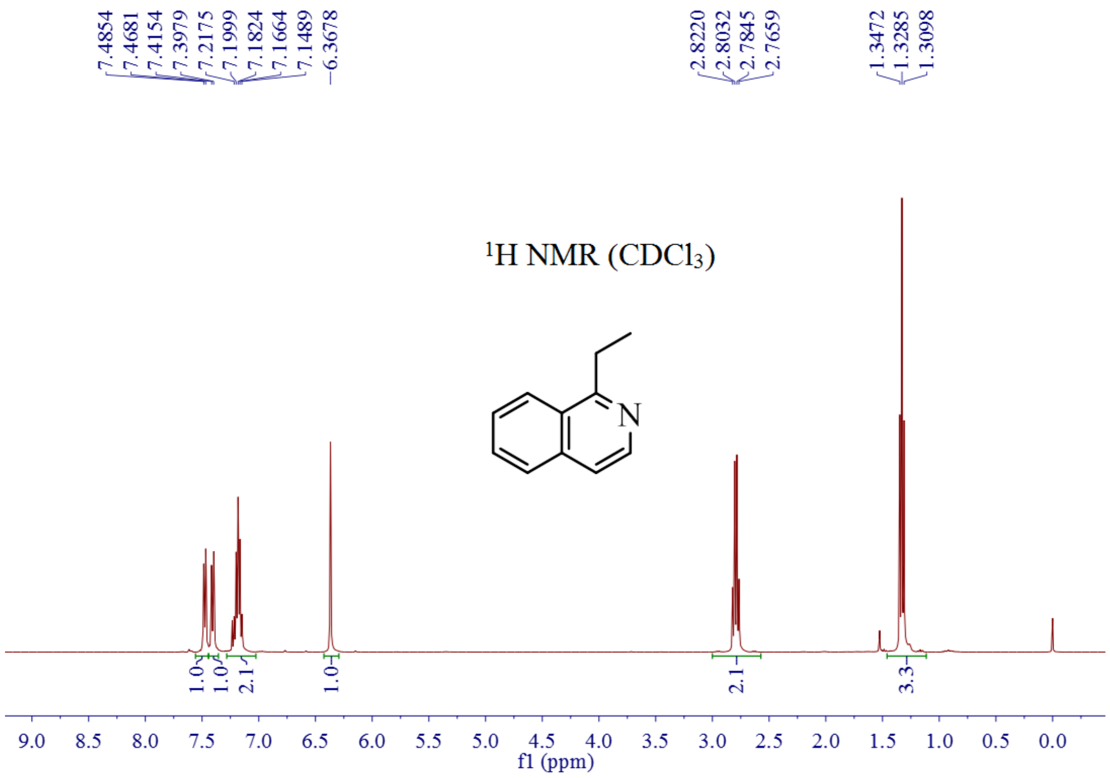
7.4854
7.4681
7.4154
7.3979
7.2175
7.1999
7.1824
7.1664
7.1489
6.3678
2.8220
2.8032
2.7845
2.7659
1.3472
1.3285
1.3098
^{1}H NMR (CDCl$_3$)
N
1.0
1.0
2.1
1.0
2.1
3.3
9.0 8.5 8.0 7.5 7.0 6.5 6.0 5.5 5.0 4.5 4.0 3.5 3.0 2.5 2.0 1.5 1.0 0.5 0.0
f1 (ppm)

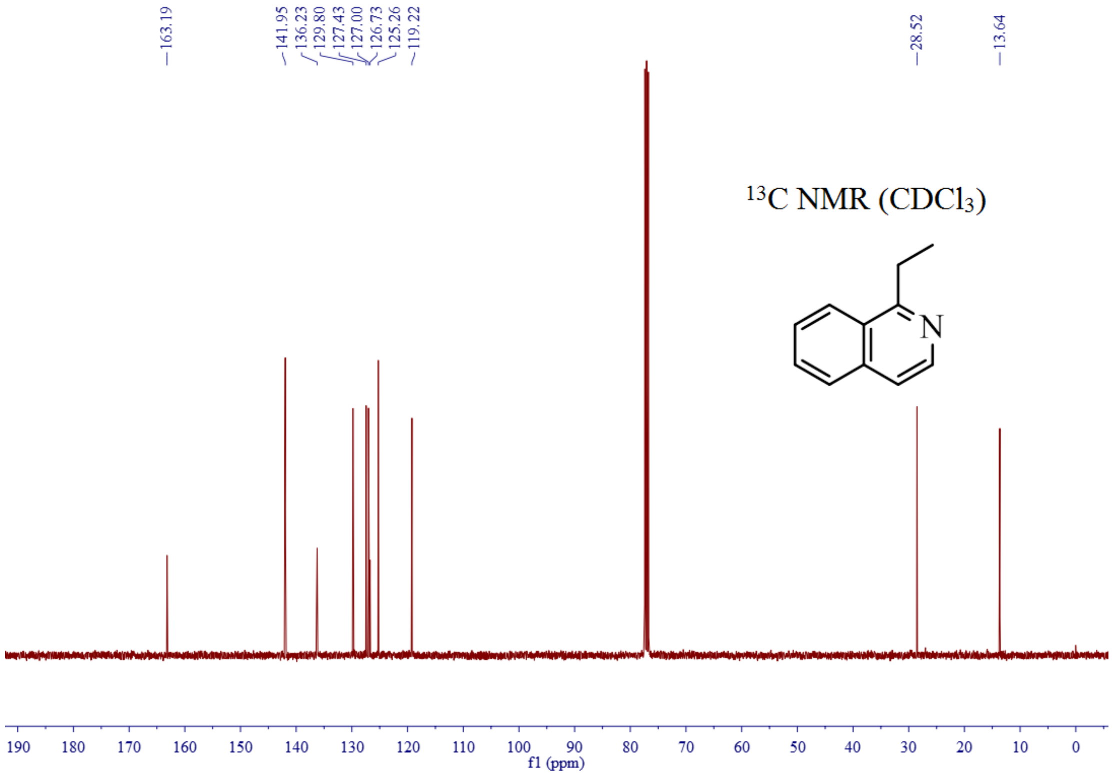
163.19
141.95
136.23
129.80
127.43
127.00
126.73
125.26
119.22
28.52
13.64
^{13}C NMR (CDCl$_3$)
N
190 180 170 160 150 140 130 120 110 100 90 80 70 60 50 40 30 20 10 0
f1 (ppm)

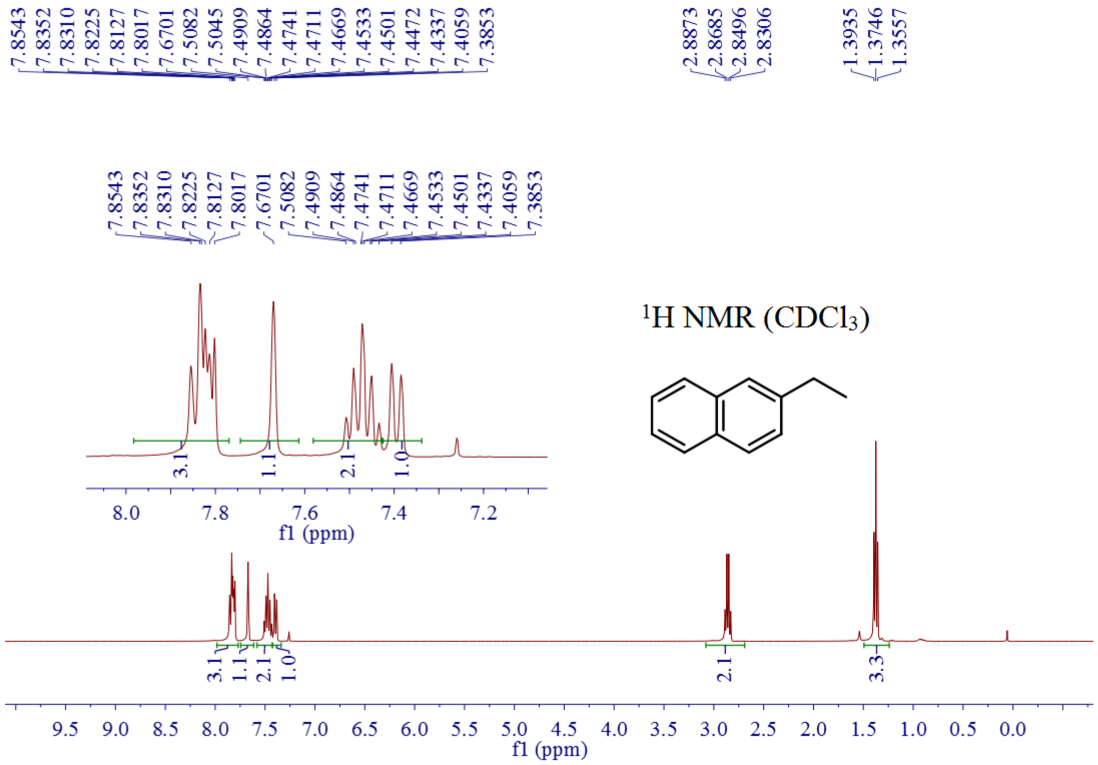
7.8543 7.8352 7.8310 7.8225 7.8127 7.8017 7.6701 7.5082 7.5045 7.4909 7.4864 7.4741 7.4711 7.4669 7.4533 7.4501 7.4472 7.4337 7.4059 7.3853
2.8873 2.8685 2.8496 2.8306
1.3935 1.3746 1.3557
1H NMR (CDCl3)
3.1 1.1 2.1 1.0
2.1
3.3
f1 (ppm)

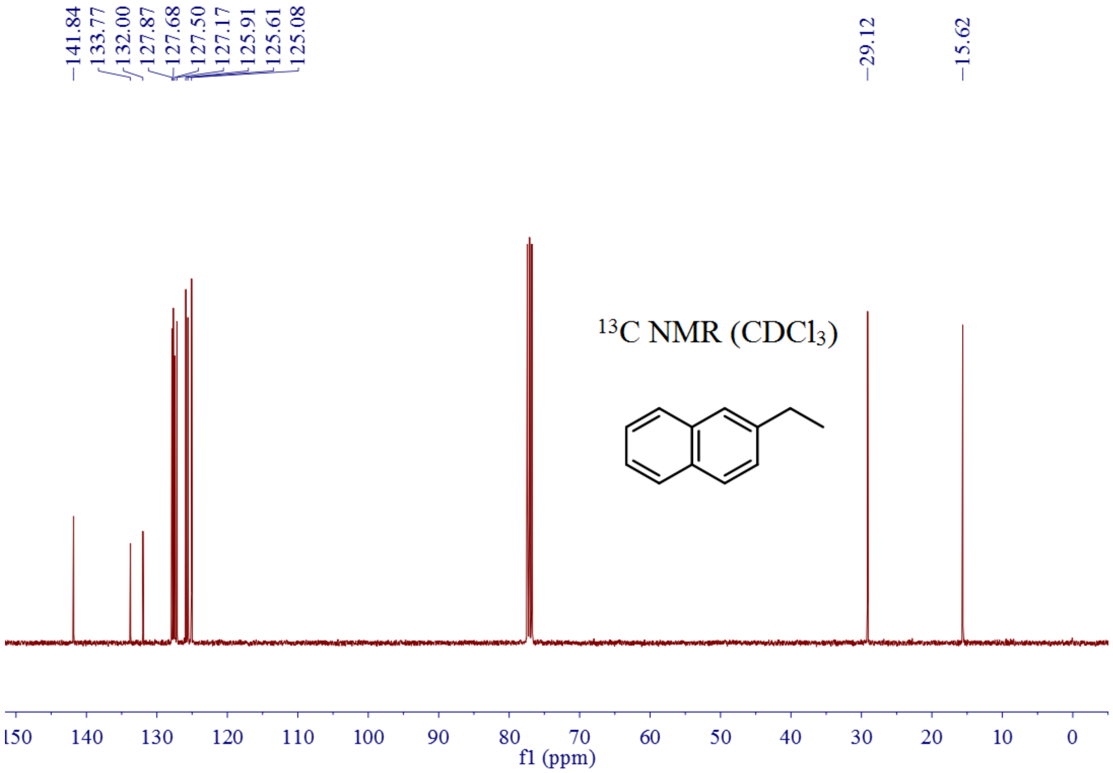
141.84 133.77 132.00 127.87 127.68 127.50 127.17 125.91 125.61 125.08
29.12
15.62
13C NMR (CDCl3)
f1 (ppm)

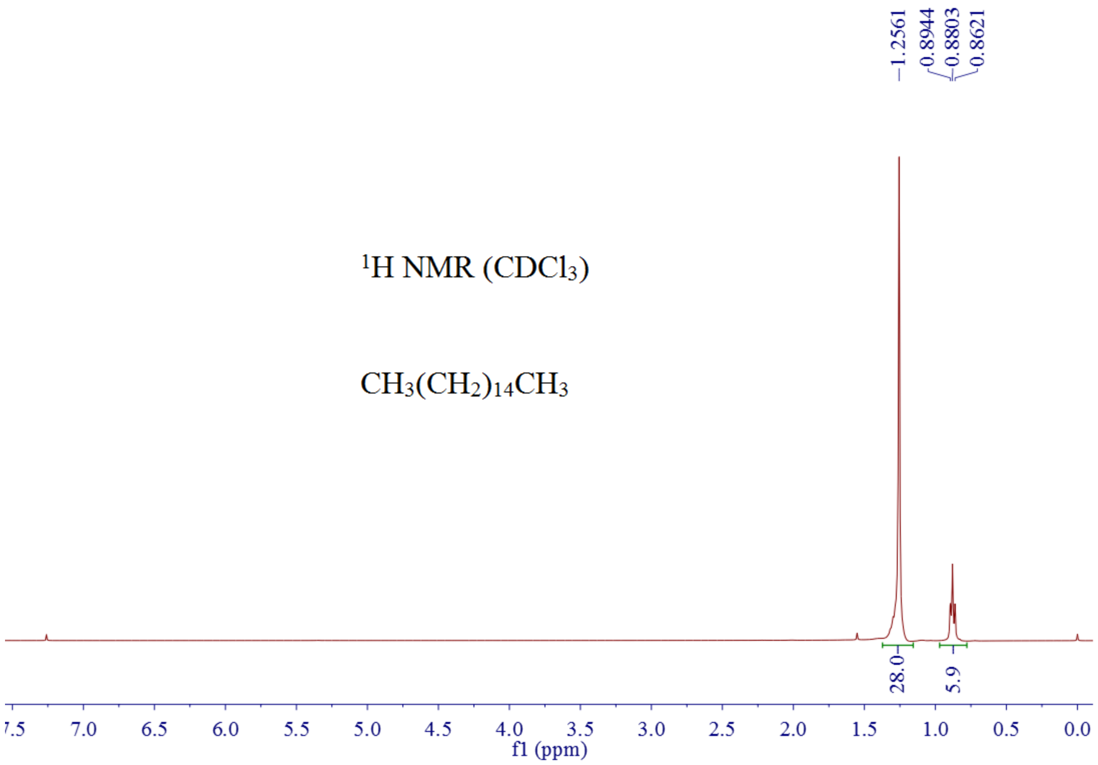

1.2561
0.8944
0.8803
0.8621
1H NMR (CDCl3)
CH3(CH2)14CH3
28.0
5.9
7.5 7.0 6.5 6.0 5.5 5.0 4.5 4.0 3.5 3.0 2.5 2.0 1.5 1.0 0.5 0.0
f1 (ppm)

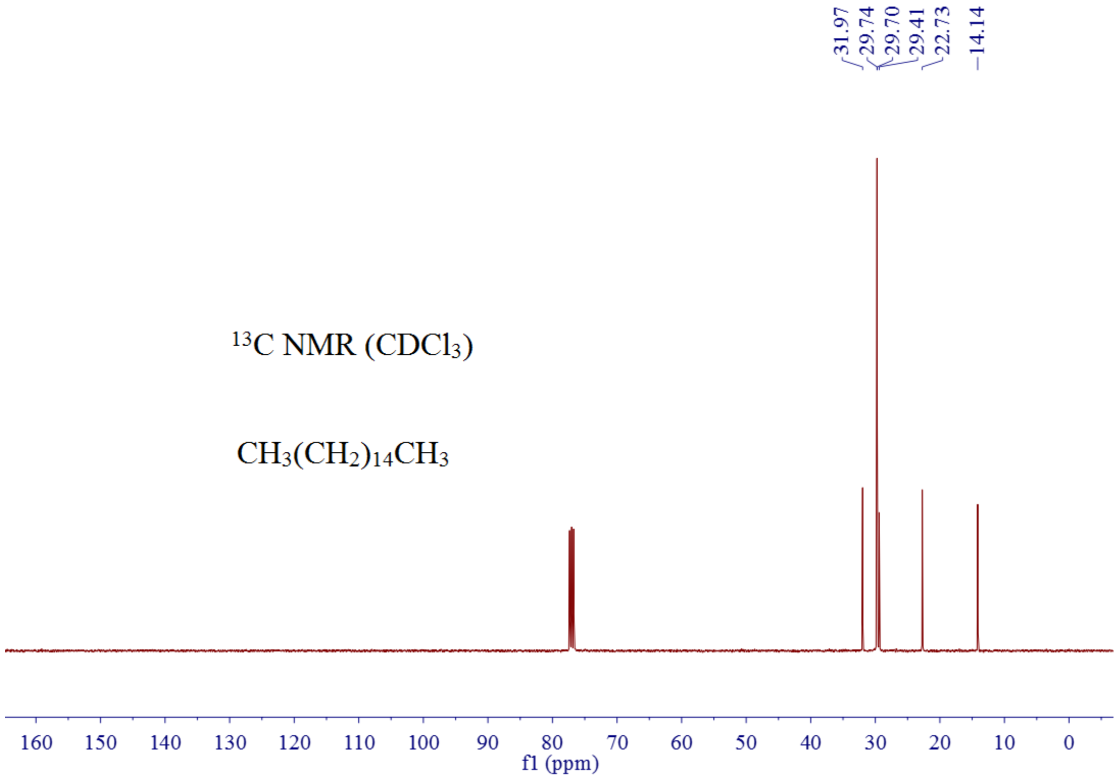

31.97
29.74
29.70
29.41
22.73
14.14
13C NMR (CDCl3)
CH3(CH2)14CH3
160 150 140 130 120 110 100 90 80 70 60 50 40 30 20 10 0
f1 (ppm)

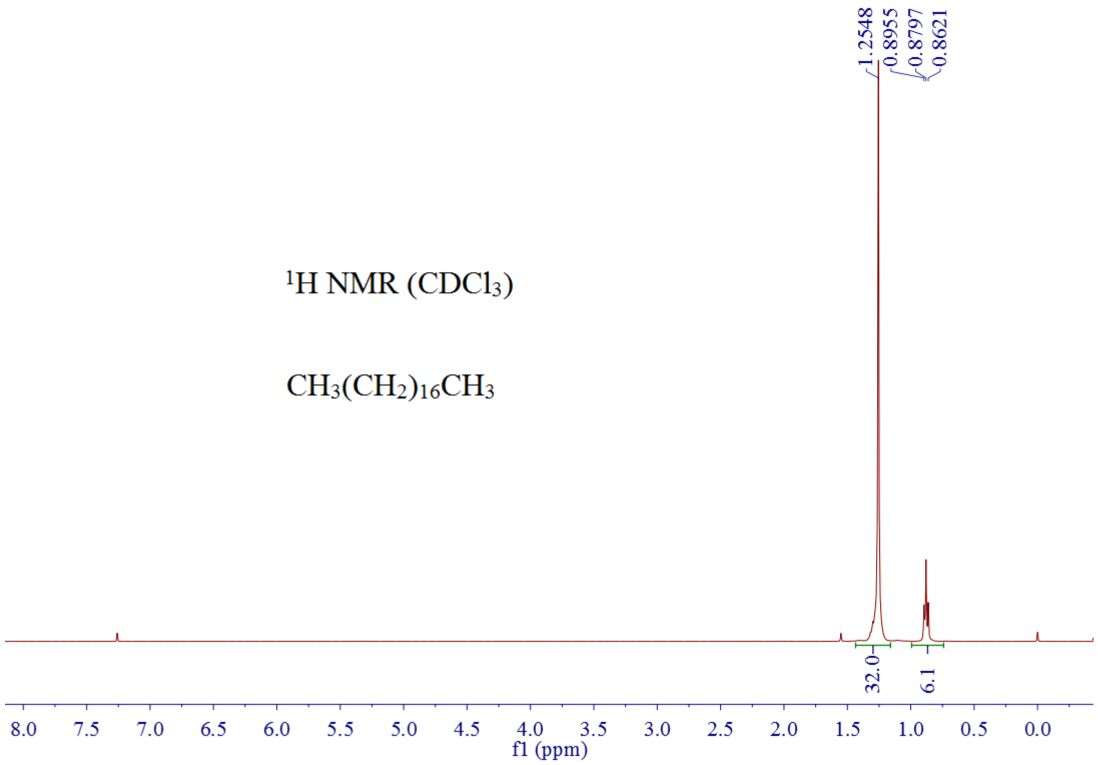
1.2548
0.8955
0.8797
0.8621
1H NMR (CDCl3)
CH3(CH2)16CH3
32.0
6.1
8.0 7.5 7.0 6.5 6.0 5.5 5.0 4.5 4.0 3.5 3.0 2.5 2.0 1.5 1.0 0.5 0.0
f1 (ppm)

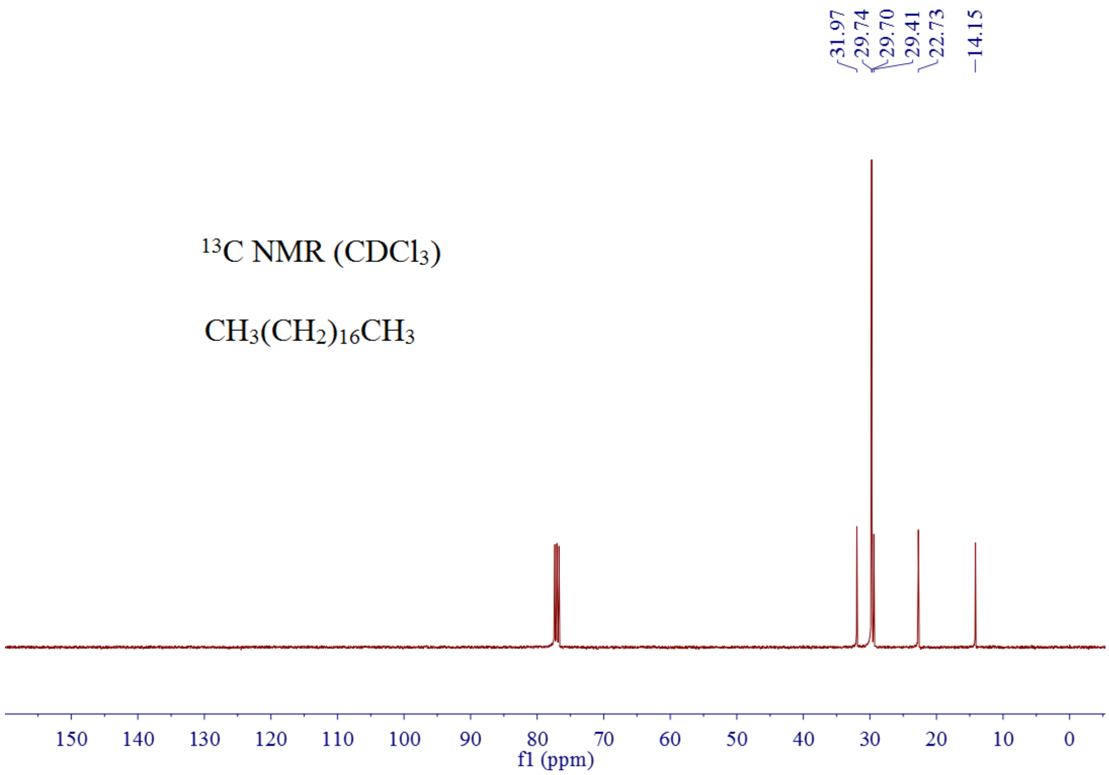
31.97
29.74
29.70
29.41
22.73
14.15
13C NMR (CDCl3)
CH3(CH2)16CH3
150 140 130 120 110 100 90 80 70 60 50 40 30 20 10 0
f1 (ppm)

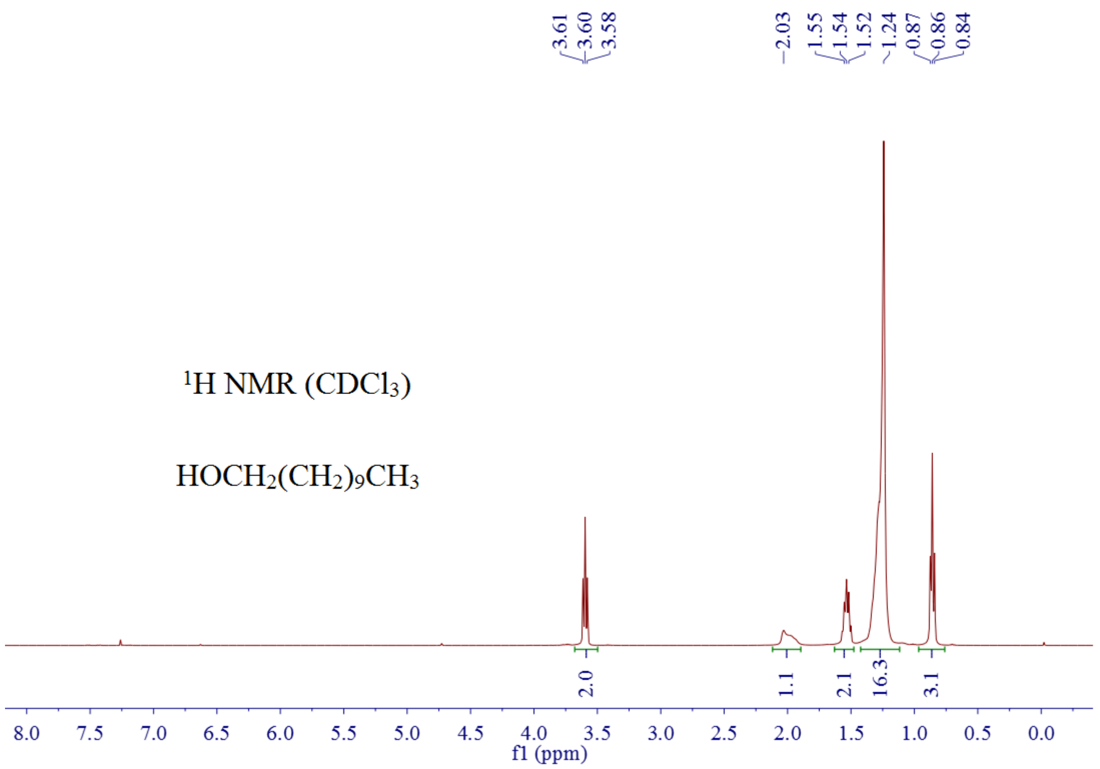
3.61
3.60
3.58
2.03
1.55
1.54
1.52
1.24
0.87
0.86
0.84
^{1}H NMR ($CDCl_3$)
$HOCH_2(CH_2)_9CH_3$
2.0
1.1
2.1
16.3
3.1
f1 (ppm)

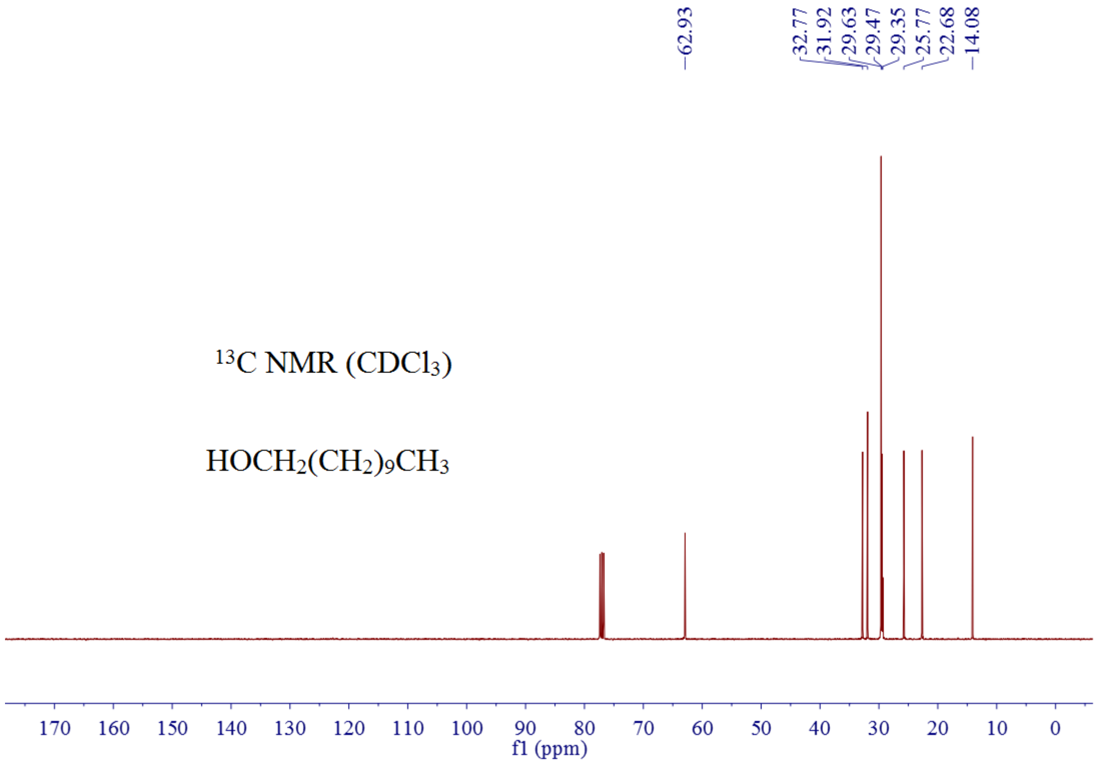
62.93
32.77
31.92
29.63
29.47
29.35
25.77
22.68
14.08
^{13}C NMR ($CDCl_3$)
$HOCH_2(CH_2)_9CH_3$
f1 (ppm)

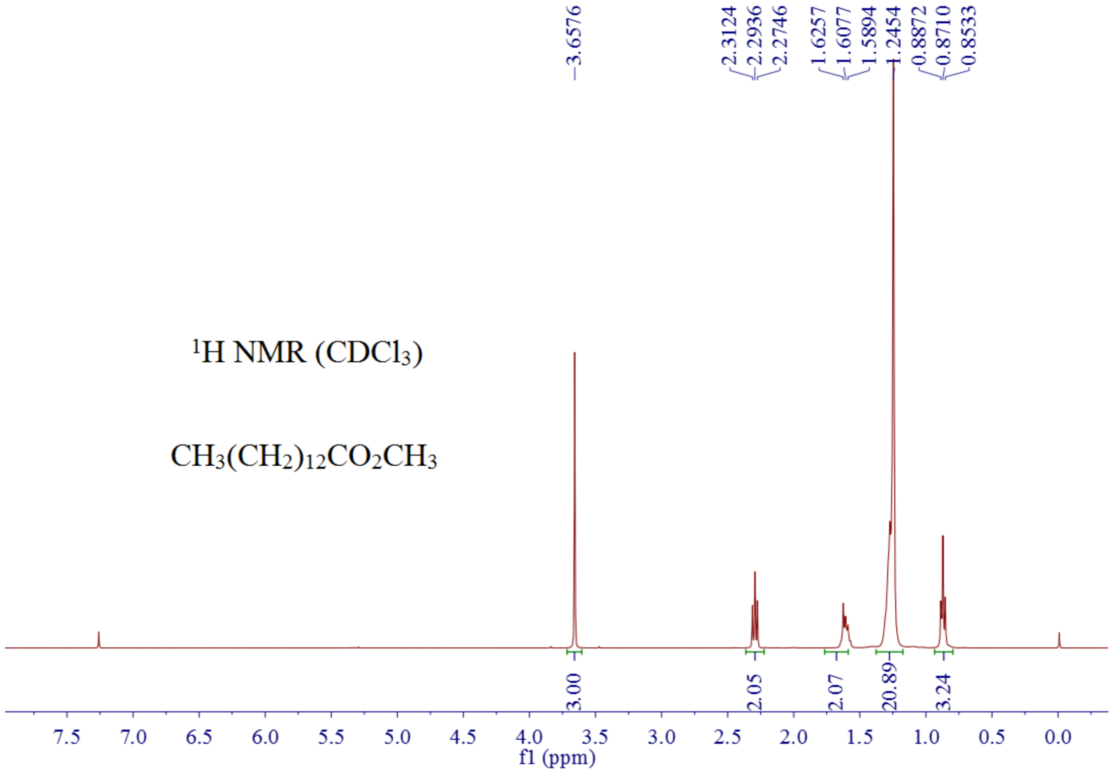
3.6576
2.3124
2.2936
2.2746
1.6257
1.6077
1.5894
1.2454
0.8872
0.8710
0.8533
^{1}H NMR ($CDCl_3$)
$CH_3(CH_2)_{12}CO_2CH_3$
3.00
2.05
2.07
20.89
3.24
7.5 7.0 6.5 6.0 5.5 5.0 4.5 4.0 3.5 3.0 2.5 2.0 1.5 1.0 0.5 0.0
f1 (ppm)

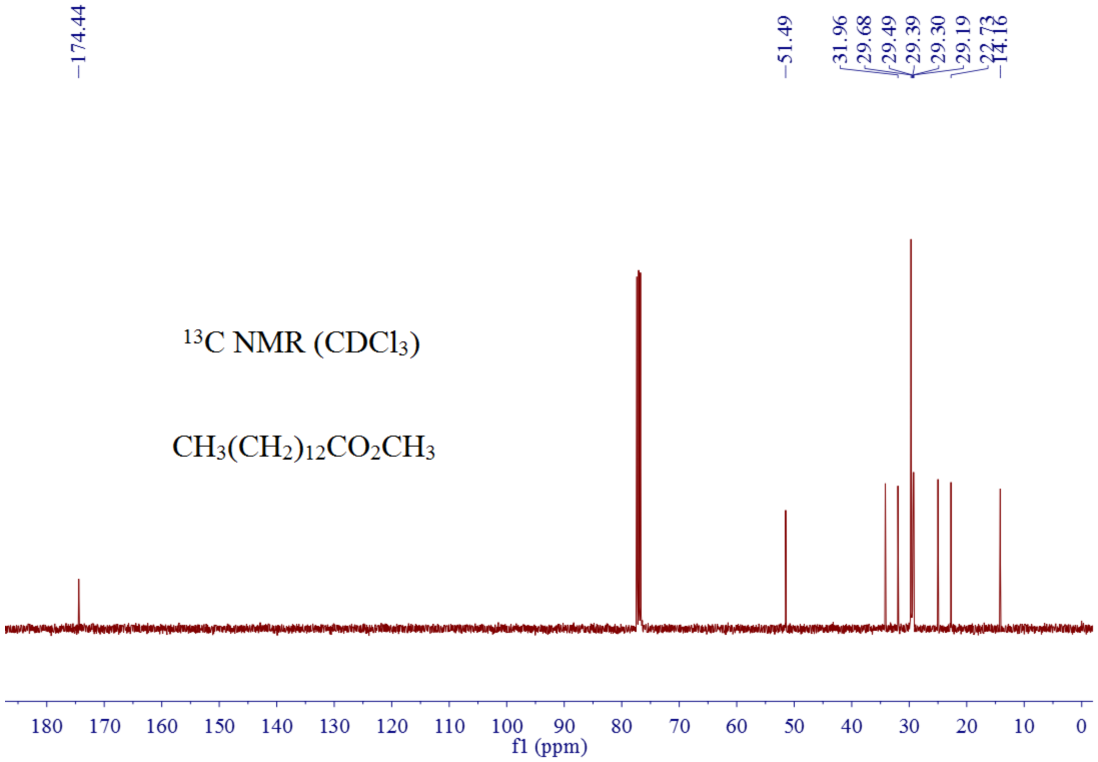
174.44
51.49
31.96
29.68
29.49
29.39
29.30
29.19
22.73
14.16
^{13}C NMR ($CDCl_3$)
$CH_3(CH_2)_{12}CO_2CH_3$
180 170 160 150 140 130 120 110 100 90 80 70 60 50 40 30 20 10 0
f1 (ppm)

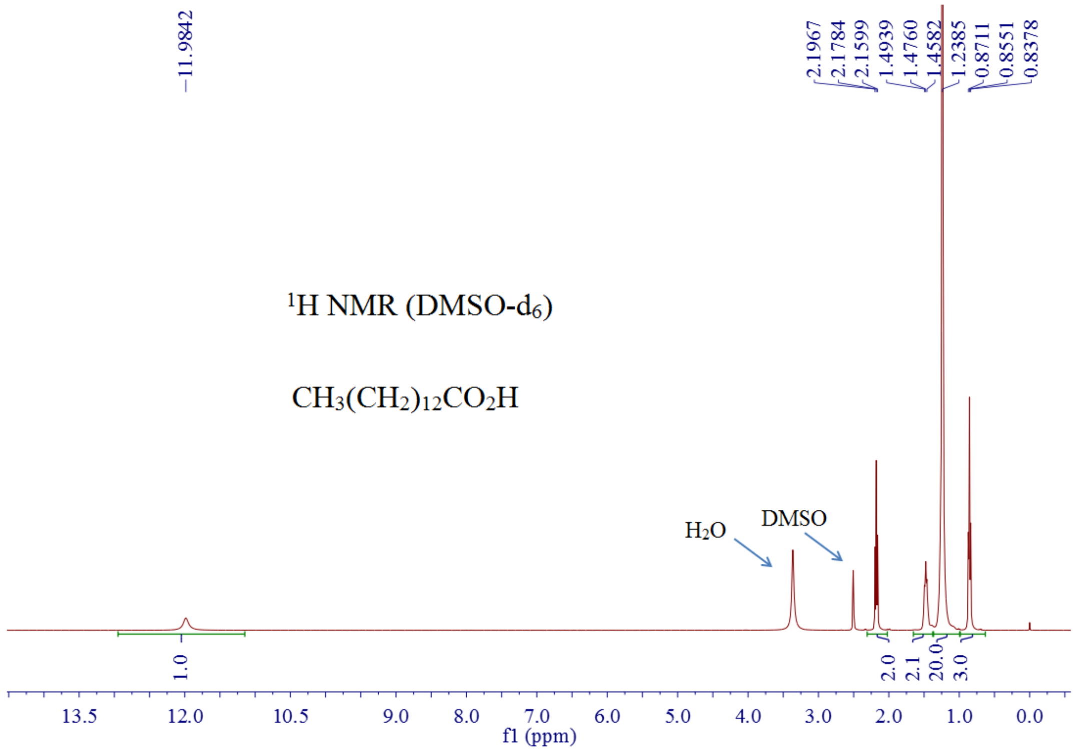

-11.9842
2.1967
2.1784
2.1599
1.4939
1.4760
1.4582
1.2385
0.8711
0.8551
0.8378
¹H NMR (DMSO-d₆)
CH3(CH2)12CO2H
H2O
DMSO
1.0
2.0
2.1
20.0
3.0
13.5
12.0
10.5
9.0
8.0
7.0
6.0
5.0
4.0
3.0
2.0
1.0
0.0
f1 (ppm)

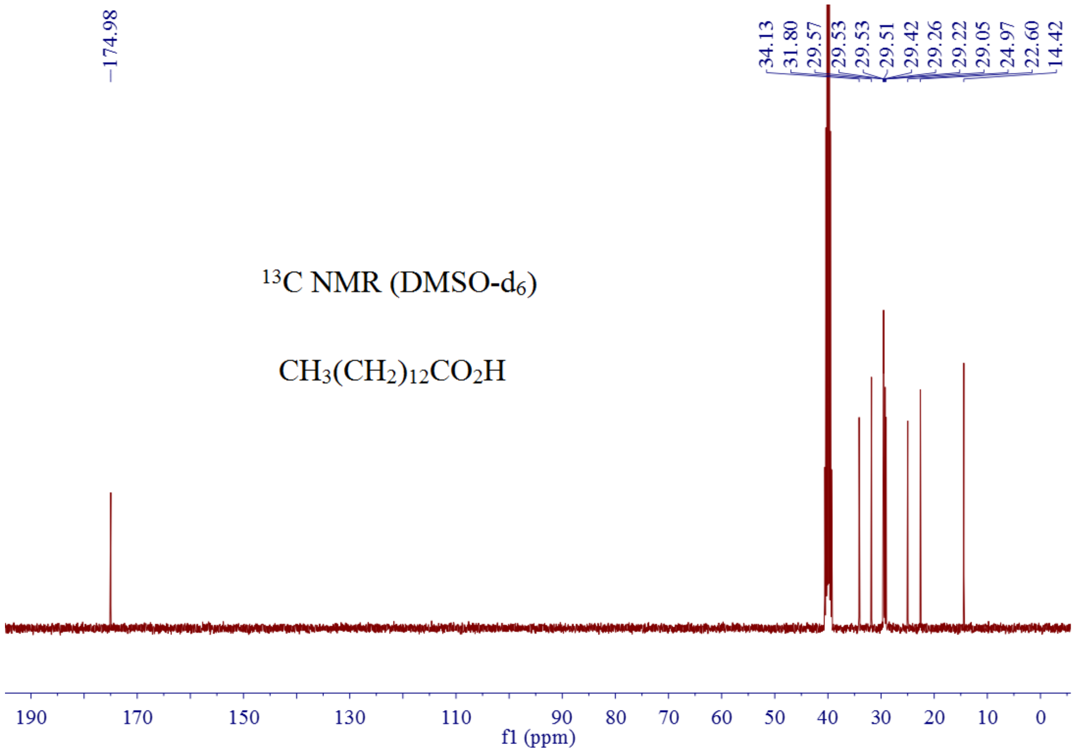

-174.98
34.13
31.80
29.57
29.53
29.53
29.51
29.42
29.26
29.22
29.05
24.97
22.60
14.42
¹³C NMR (DMSO-d₆)
CH3(CH2)12CO2H
190
170
150
130
110
90
80
70
60
50
40
30
20
10
0
f1 (ppm)

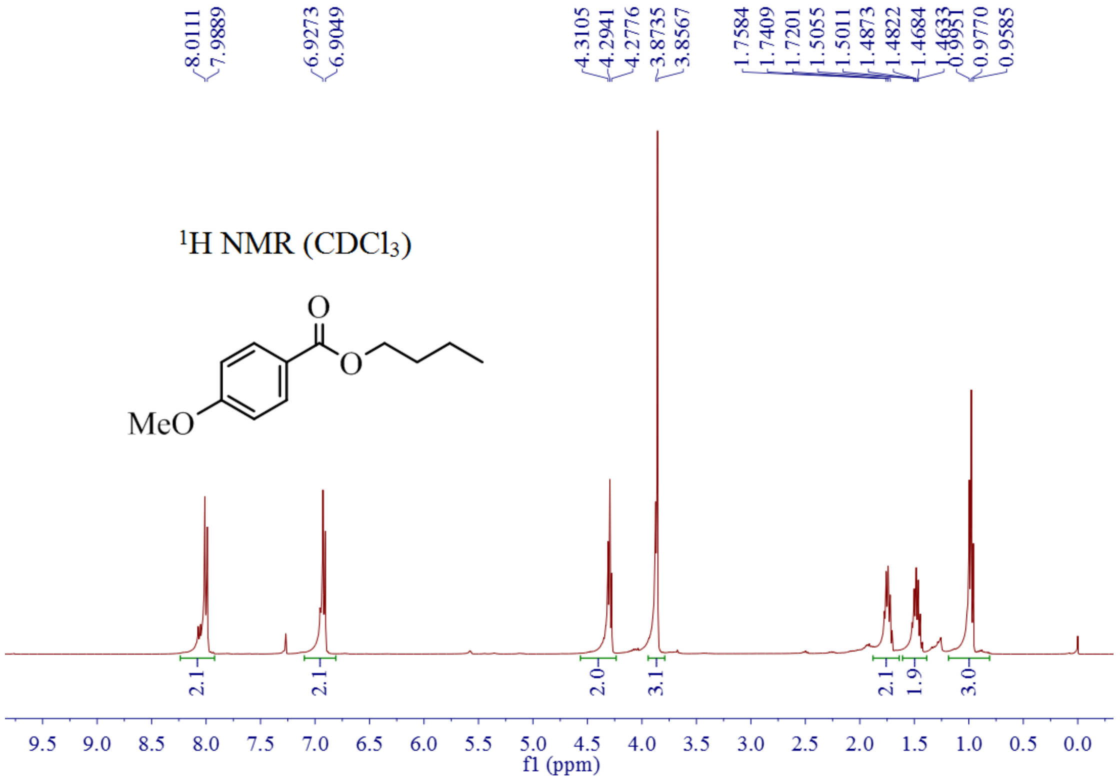
8.0111
7.9889
6.9273
6.9049
4.3105
4.2941
4.2776
3.8735
3.8567
1.7584
1.7409
1.7201
1.5055
1.5011
1.4873
1.4822
1.4684
1.4633
0.9951
0.9770
0.9585
1H NMR (CDCl3)
O
O
MeO
2.1
2.1
2.0
3.1
2.1
1.9
3.0
9.5
9.0
8.5
8.0
7.5
7.0
6.5
6.0
5.5
5.0
4.5
4.0
3.5
3.0
2.5
2.0
1.5
1.0
0.5
0.0
f1 (ppm)

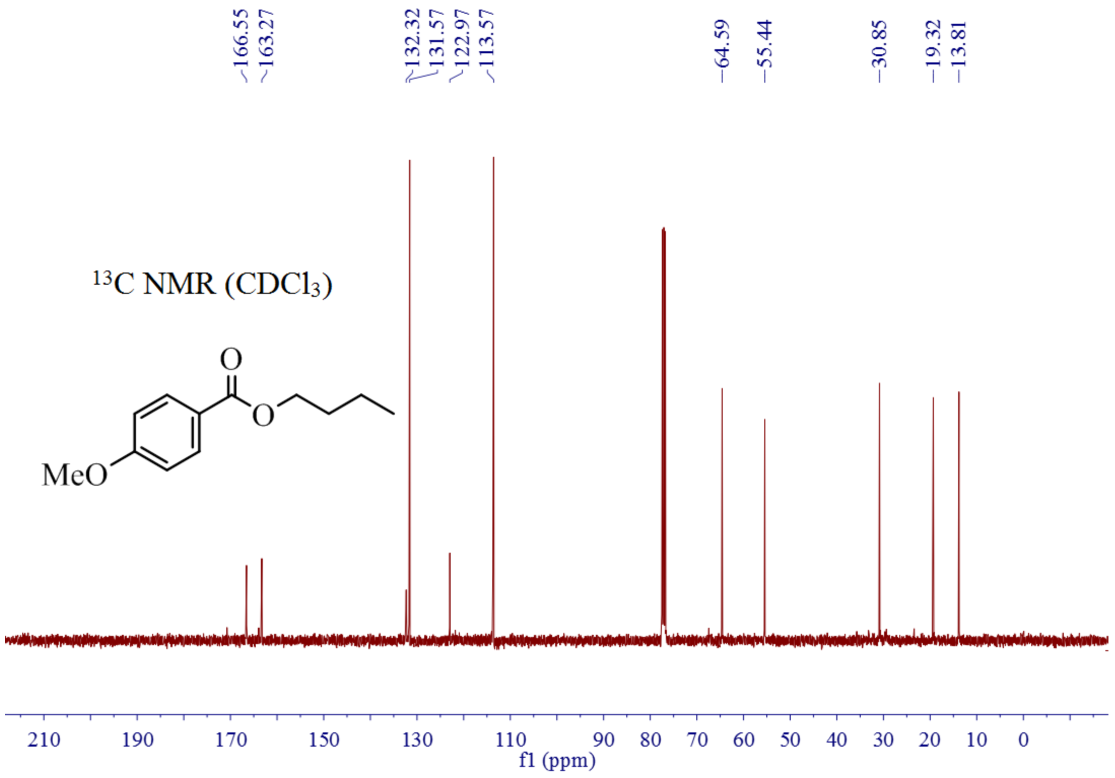
166.55
163.27
132.32
131.57
122.97
113.57
64.59
55.44
30.85
19.32
13.81
13C NMR (CDCl3)
O
O
MeO
210
190
170
150
130
110
90
80
70
60
50
40
30
20
10
0
f1 (ppm)

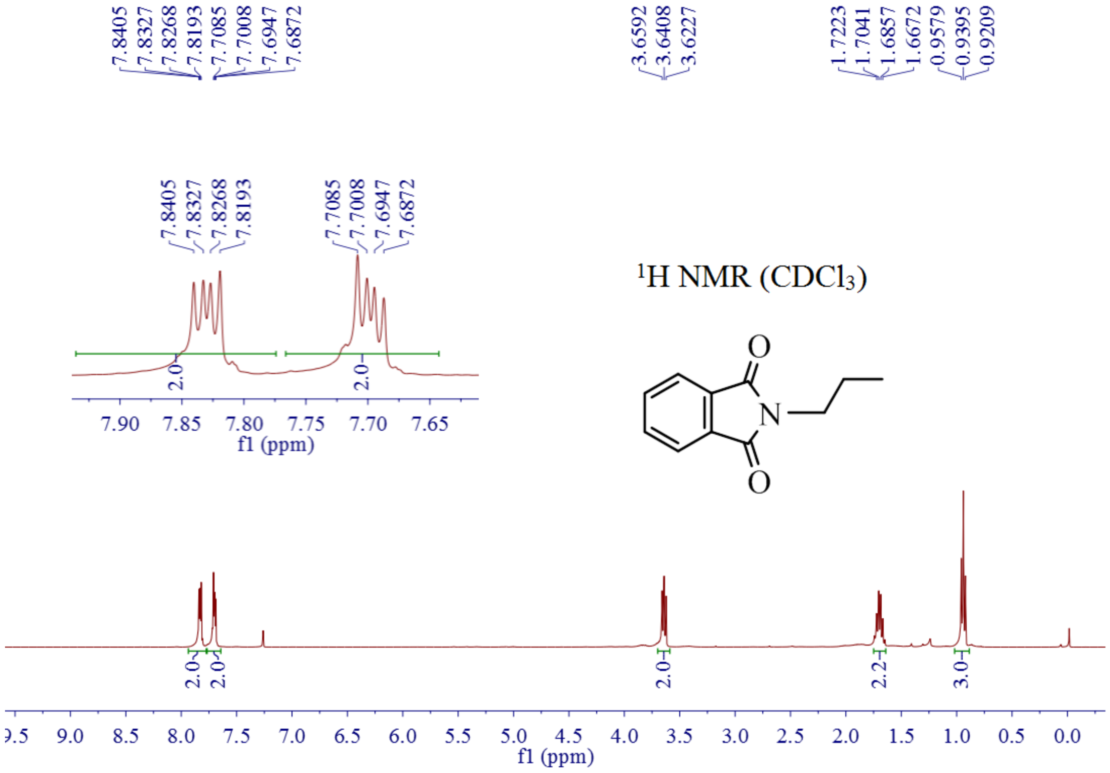
7.8405
7.8327
7.8268
7.8193
7.7085
7.7008
7.6947
7.6872
3.6592
3.6408
3.6227
1.7223
1.7041
1.6857
1.6672
0.9579
0.9395
0.9209
1H NMR (CDCl3)
O
N
O
2.0
2.0
2.0
2.0
2.0
2.2
3.0
7.90 7.85 7.80 7.75 7.70 7.65
f1 (ppm)
9.5 9.0 8.5 8.0 7.5 7.0 6.5 6.0 5.5 5.0 4.5 4.0 3.5 3.0 2.5 2.0 1.5 1.0 0.5 0.0
f1 (ppm)

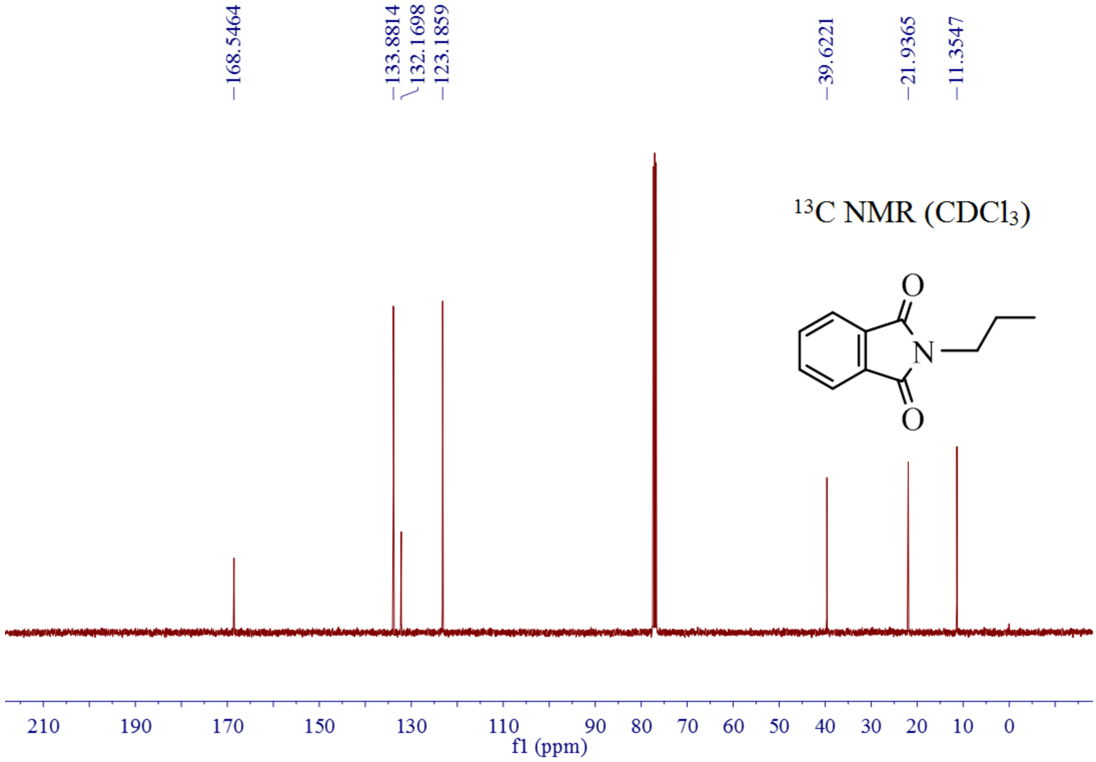
168.5464
133.8814
132.1698
123.1859
39.6221
21.9365
11.3547
13C NMR (CDCl3)
O
N
O
210 190 170 150 130 110 90 80 70 60 50 40 30 20 10 0
f1 (ppm)

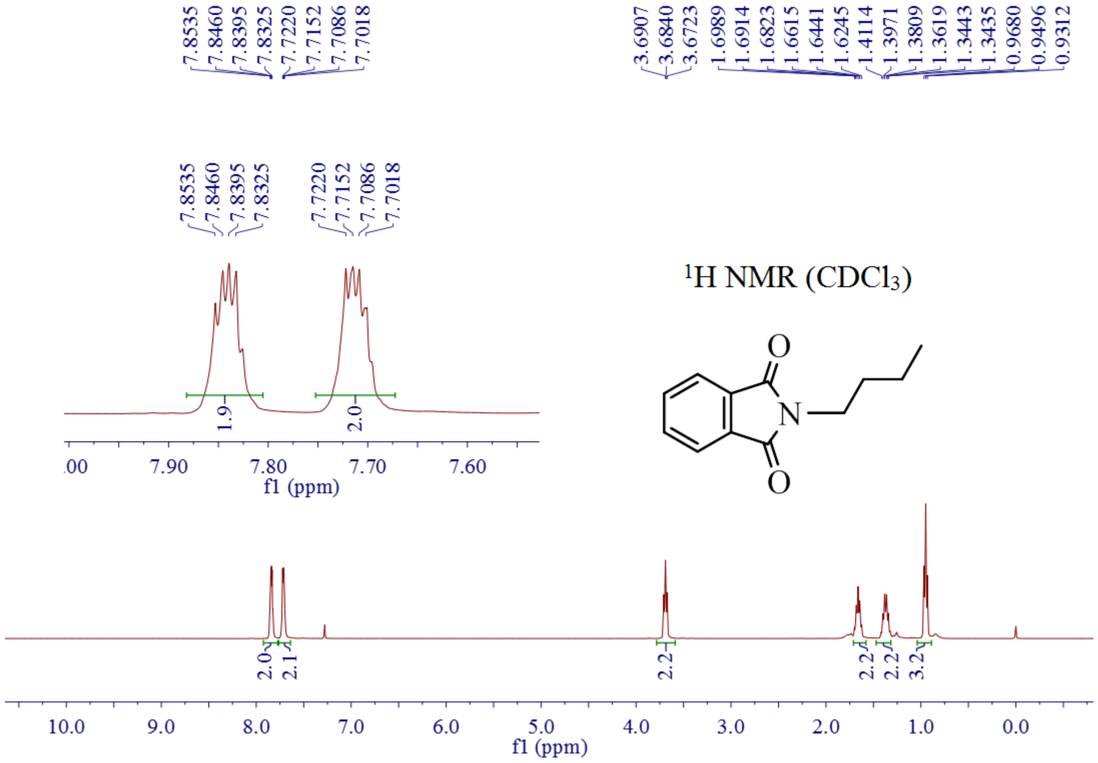
7.8535
7.8460
7.8395
7.8325
7.7220
7.7152
7.7086
7.7018
3.6907
3.6840
3.6723
1.6989
1.6914
1.6823
1.6615
1.6441
1.6245
1.4114
1.3971
1.3809
1.3619
1.3443
1.3435
0.9680
0.9496
0.9312
^{1}H NMR (CDCl3)
O
N
O
1.9
2.0
7.90
7.80
7.70
7.60
f1 (ppm)
2.0
2.1
2.2
2.2
2.2
3.2
10.0
9.0
8.0
7.0
6.0
5.0
4.0
3.0
2.0
1.0
0.0
f1 (ppm)

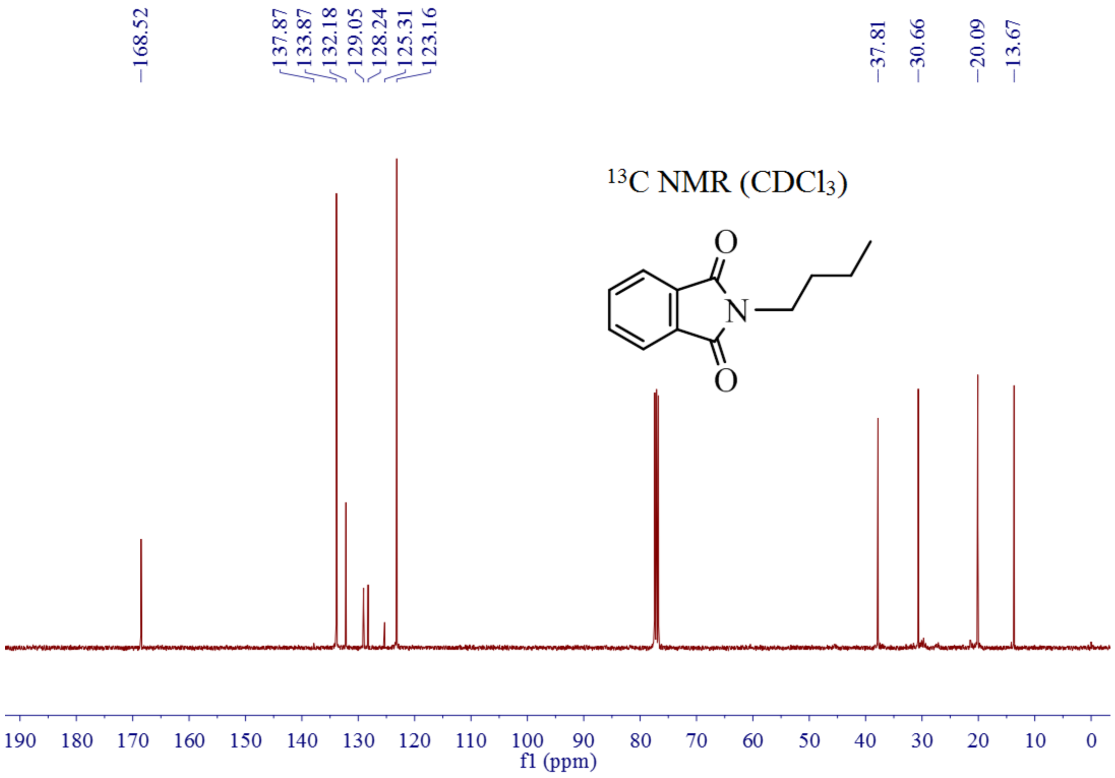
168.52
137.87
133.87
132.18
129.05
128.24
125.31
123.16
37.81
30.66
20.09
13.67
^{13}C NMR (CDCl3)
O
N
O
190
180
170
160
150
140
130
120
110
100
90
80
70
60
50
40
30
20
10
0
f1 (ppm)

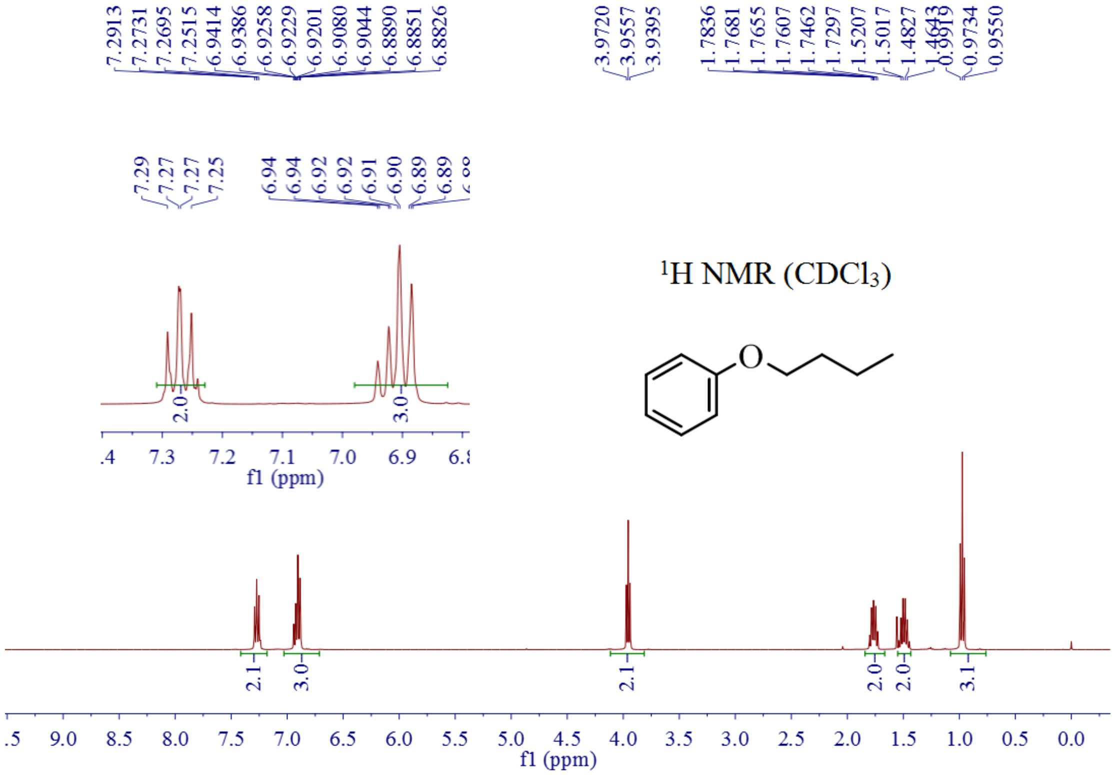
7.2913
7.2731
7.2695
7.2515
6.9414
6.9386
6.9258
6.9229
6.9201
6.9080
6.9044
6.8890
6.8851
6.8826
3.9720
3.9557
3.9395
1.7836
1.7681
1.7655
1.7607
1.7462
1.7297
1.5207
1.5017
1.4827
0.9734
0.9550
1H NMR (CDCl3)
O
f1 (ppm)

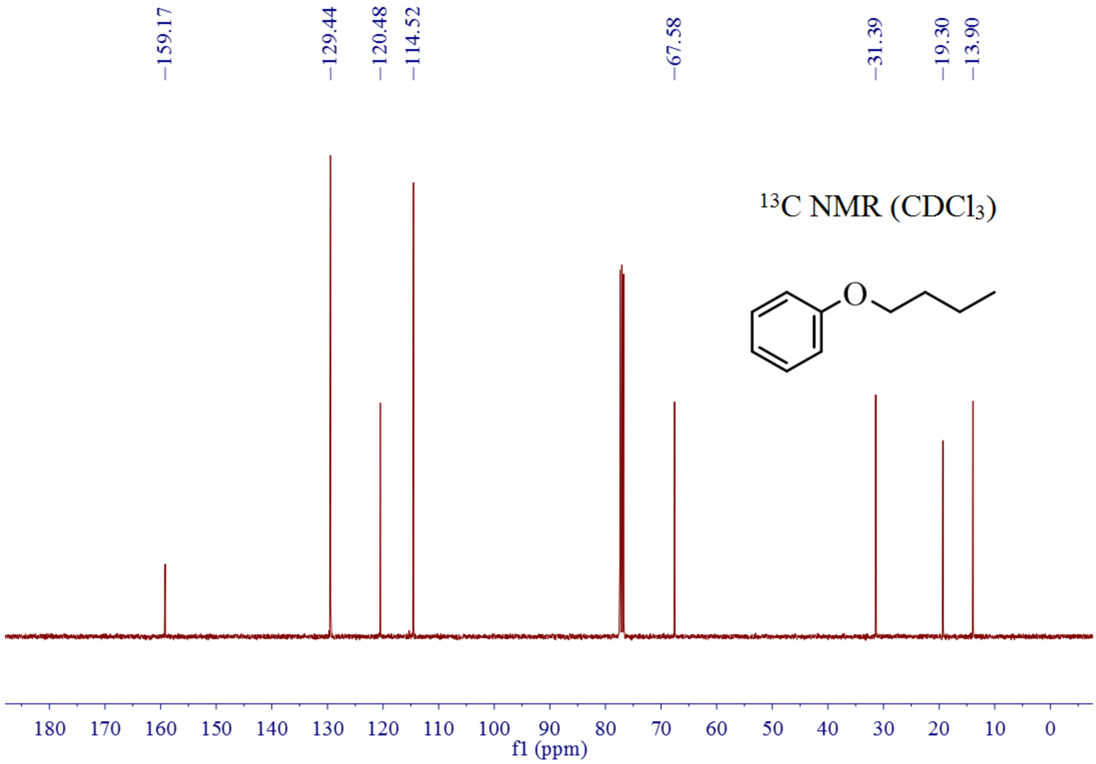
159.17
129.44
120.48
114.52
67.58
31.39
19.30
13.90
13C NMR (CDCl3)
O
f1 (ppm)

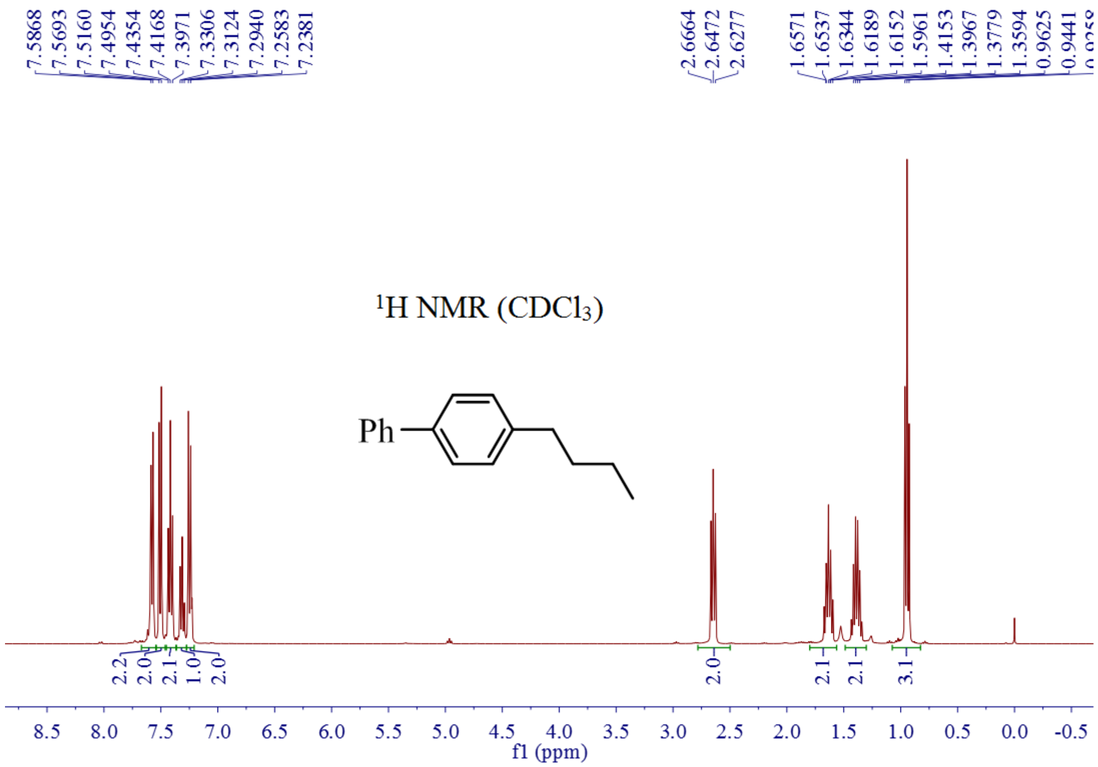
7.5868
7.5693
7.5160
7.4954
7.4354
7.4168
7.3971
7.3306
7.3124
7.2940
7.2583
7.2381
2.6664
2.6472
2.6277
1.6571
1.6537
1.6344
1.6189
1.6152
1.5961
1.4153
1.3967
1.3779
1.3594
0.9625
0.9441
¹H NMR (CDCl₃)
Ph
2.2
2.0
2.1
1.0
2.0
2.0
2.1
2.1
3.1
8.5 8.0 7.5 7.0 6.5 6.0 5.5 5.0 4.5 4.0 3.5 3.0 2.5 2.0 1.5 1.0 0.5 0.0 -0.5
f1 (ppm)

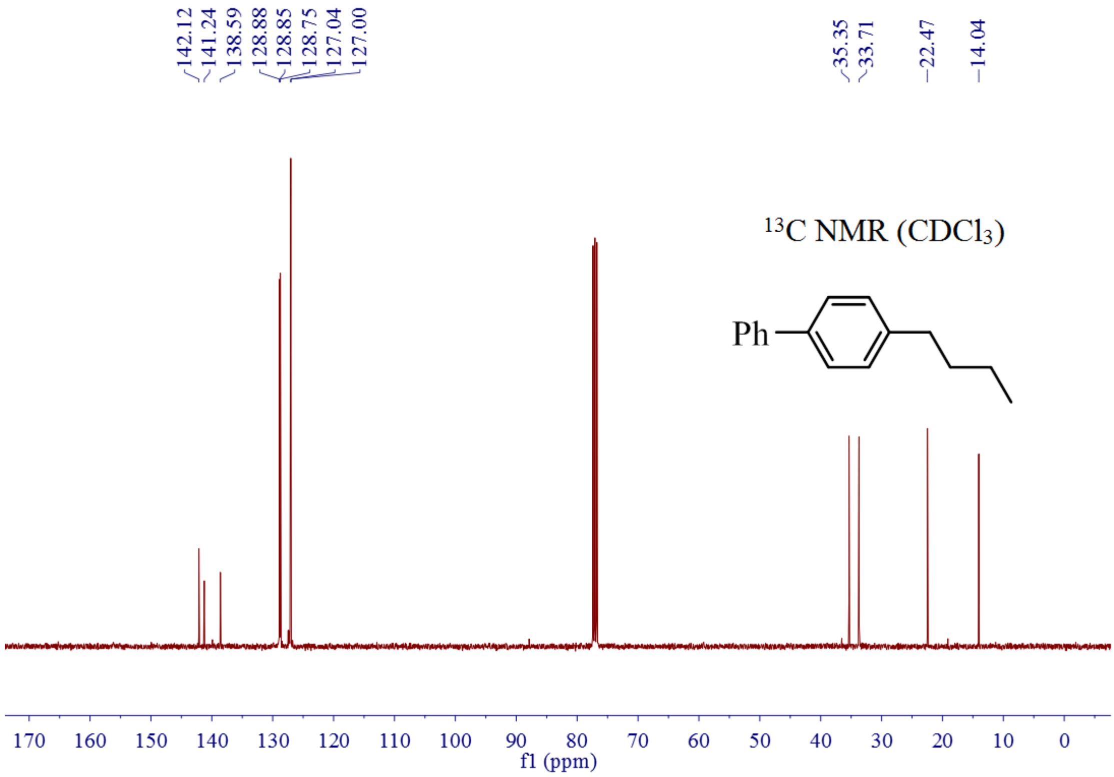
142.12
141.24
138.59
128.88
128.85
128.75
127.04
127.00
35.35
33.71
22.47
14.04
¹³C NMR (CDCl₃)
Ph
170 160 150 140 130 120 110 100 90 80 70 60 50 40 30 20 10 0
f1 (ppm)

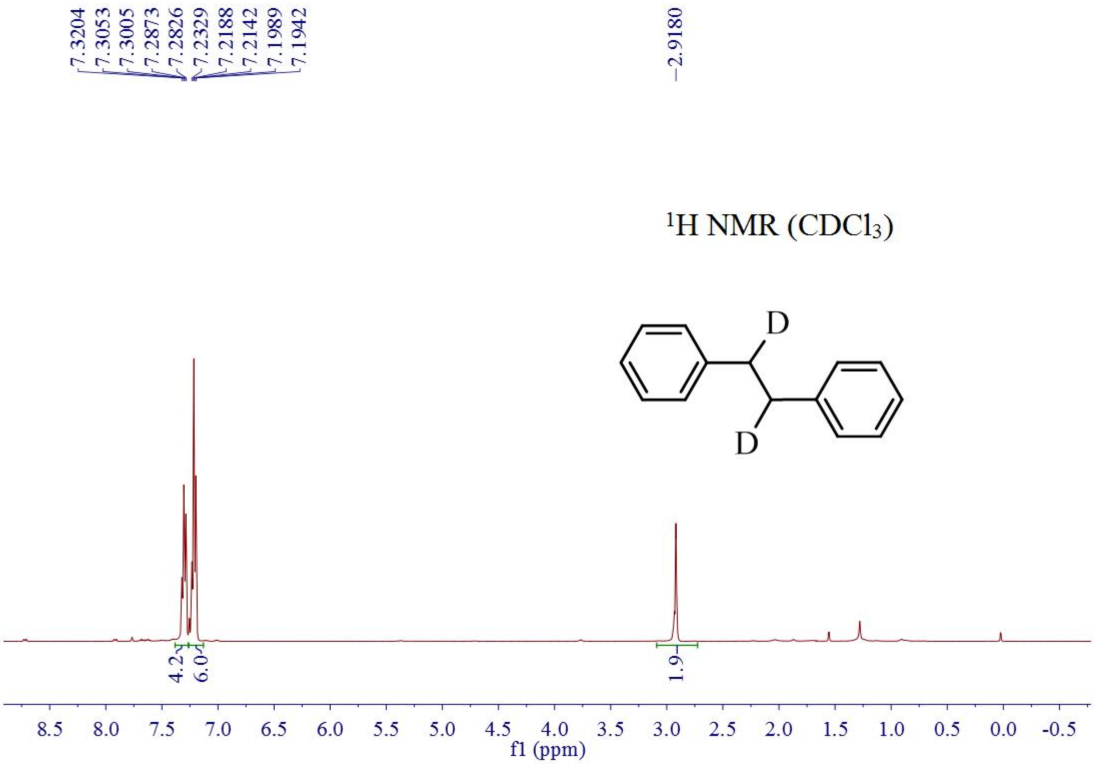

7.3204
7.3053
7.3005
7.2873
7.2826
7.2329
7.2188
7.2142
7.1989
7.1942
2.9180
^{1}H NMR ($CDCl_3$)
D
D
4.2
6.0
1.9
8.5 8.0 7.5 7.0 6.5 6.0 5.5 5.0 4.5 4.0 3.5 3.0 2.5 2.0 1.5 1.0 0.5 0.0 -0.5
f1 (ppm)

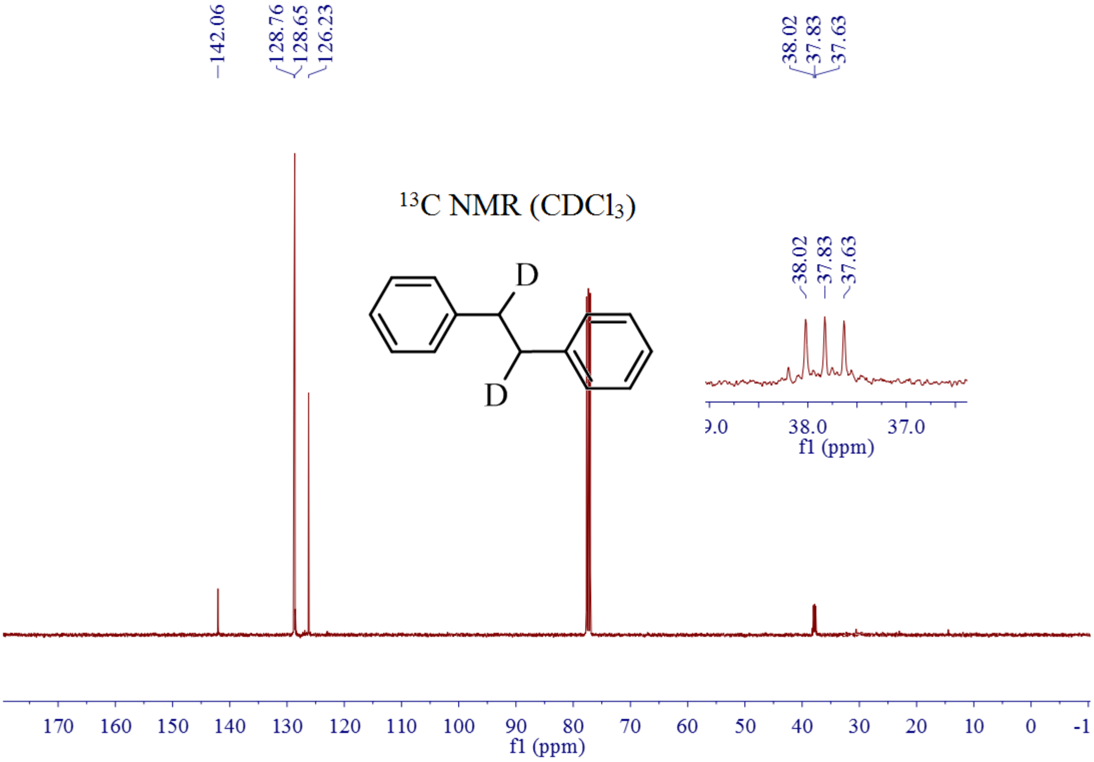

142.06
128.76
128.65
126.23
38.02
37.83
37.63
^{13}C NMR ($CDCl_3$)
D
D
38.02
37.83
37.63
38.0
37.0
f1 (ppm)
170 160 150 140 130 120 110 100 90 80 70 60 50 40 30 20 10 0 -1
f1 (ppm)
